# Empagliflozin and Ultrafiltration Volume in Patients Undergoing Peritoneal Dialysis

**DOI:** 10.1016/j.ekir.2025.09.049

**Published:** 2025-10-10

**Authors:** Yohei Doi, Maki Shinzawa, Tetsuya Arisato, Hideaki Oka, Yoshiyasu Ueda, Ayumi Matsumoto, Harumi Kitamura, Takayuki Hamano, Yumi Nakazono, Yoichi Nishiya, Nobuhiro Hashimoto, Miki Matsuo, Takuryu Sonoura, Chikako Monden, Taro Kamimura, Terumasa Hayashi, Fumiki Yoshihara, Yoshitaka Isaka, EMPOWERED Investigators

**Affiliations:** 1 Department of Nephrology, Graduate School of Medicine, The University of Osaka, Suita, Osaka, Japan; 2 Department of Cardiology, Pulmonology, Hypertension and Nephrology, Ehime University Graduate School of Medicine, Toon, Ehime, Japan; 3 Health and Counseling Center, The University of Osaka, Toyonaka, Osaka, Japan; 4 Division of Nephrology and Hypertension, National Cerebral and Cardiovascular Center, Suita, Osaka, Japan; 5 Division of Kidney Center, Matsuyama Red Cross Hospital, Matsuyama, Ehime, Japan; 6 Department of Kidney Disease and Hypertension, Osaka General Medical Center, Osaka, Osaka, Japan; 7 Department of Clinical Quality Management, The University of Osaka Hospital, Suita, Osaka, Japan; 8 Department of Nephrology, Nagoya City University Graduate School of Medical Sciences, Nagoya, Aichi, Japan; 9 Medicine Division, Nippon Boehringer Ingelheim Co., Ltd., Shinagawa-ku, Tokyo, Japan; 10 Department of Internal Medicine, Kisei Hospital, Osaka, Osaka, Japan

**Keywords:** peritoneal dialysis, SGLT2 inhibitor, ultrafiltration

## Abstract

**Introduction:**

Sodium-glucose cotransporter 2 (SGLT2) is expressed in the human peritoneum, and preclinical studies suggest that SGLT2 inhibitors may enhance ultrafiltration by reducing glucose absorption from peritoneal dialysis (PD) solutions. This study evaluated whether empagliflozin increases ultrafiltration volume (UFV) in patients on PD.

**Methods:**

In this multicenter, randomized (1:1), double-blind, placebo-controlled crossover trial, patients on PD received empagliflozin (10 mg/d) or placebo for 8 weeks, separated by a 4-week washout period. The primary outcome was the change in daily UFV from baseline to week 8 in each treatment period.

**Results:**

Of 40 randomized patients, 37 received treatment and were analyzed. The median age was 66 years, PD duration was 2.8 years, and 46% were female. At baseline, median daily UFV was 476 ml with 4500 ml of glucose-based dialysate. At week 8, change in UFV did not significantly differ between treatments (−38 ml; 95% confidence interval [CI]: −120 to 44; *P* = 0.36). No heterogeneity was observed in subgroups by diabetes or peritoneal membrane transport characteristics. Empagliflozin had no significant effect on UFV or dialysate glucose or sodium concentrations, assessed by peritoneal equilibration test (PET). Urinary glucose excretion increased by 2698 mg/d (95% CI: 343–5052), without changes in urine volume, sodium, or protein excretion. Adverse events occurred in 47% with empagliflozin and 29% with placebo; serious adverse events occurred in 11% and 6%, respectively.

**Conclusion:**

Empagliflozin did not significantly improve UFV in patients on PD, suggesting limited short-term clinical utility.

## Graphical abstract

**Figure:**
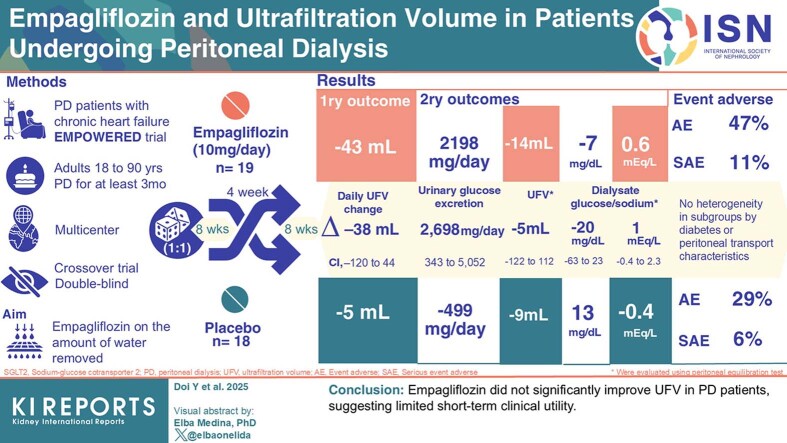

Volume overload is common in patients on PD and contributes to cardiovascular complications, technique failure, and mortality, making adequate ultrafiltration essential.^1^ Ultrafiltration in PD depends on the osmotic gradient generated by glucose-based dialysate; however, this gradient dissipates over time because of glucose absorption into the systemic circulation.^2^ Using higher-glucose dialysate to enhance ultrafiltration can accelerate peritoneal membrane damage and cause metabolic complications.^3^ These limitations highlight the need for novel therapeutic strategies to improve fluid removal without increasing glucose load.

SGLT2 is expressed in the human peritoneum,^4^, ^5^, ^6^ and a preclinical model suggests that its inhibition reduces peritoneal glucose absorption, preserving the osmotic gradient and enhancing ultrafiltration.^5^ SGLT2 inhibition may mitigate peritoneal fibrosis and angiogenesis—key drivers of long-term ultrafiltration failure.^6^^,^^7^ These findings have generated interest in SGLT2 inhibitors for PD, though human data remain limited. Some observational studies of patients on PD have reported increased UFV with SGLT2 inhibitors,^8^, ^9^, ^10^, ^11^ but the results have been inconsistent and potentially confounded.

To address this gap, we conducted a randomized, double-blind, placebo-controlled, crossover trial to determine whether empagliflozin, an SGLT2 inhibitor, could improve daily UFV in patients undergoing PD, and to assess its potential harm. We hypothesized that empagliflozin would enhance UFV by preserving the glucose-driven osmotic gradient through inhibition of peritoneal glucose absorption.

## Methods

### Study Design and Participants

The EMPOWERED trial was a multicenter, randomized, double-blind, placebo-controlled, crossover study evaluating the safety and efficacy of once-daily empagliflozin 10 mg in patients undergoing PD.^12^ A crossover design was chosen to minimize interindividual variability in UFV and enhance statistical power, anticipating a limited sample size and expected within-subject consistency. The study was conducted at 4 hospitals in Japan between December 2023 and November 2024. The study protocol has been previously published.^13^ The trial was registered in the Japan Registry of Clinical Trials (jRCTs051230081) and received approval from the Osaka University Clinical Research Review Committee (Approval No. S23004), as well as the ethics committees of all participating institutions. The study complied with the Declaration of Helsinki, and all participants provided written informed consent.

Eligible participants were adults aged 18 to 90 years who had been on PD for ≥ 3 months, were using ≥ 3 L/d of glucose-based PD solution, and had chronic heart failure per the approved indication of empagliflozin in Japan. Heart failure criteria included biomarker elevation, echocardiographic findings, or previous hospitalization (Supplementary Table S1). Key exclusions were SGLT2 inhibitor use within 3 months and peritonitis within the preceding 2 months.

### Randomization and Masking

Participants were randomly assigned (1:1) to receive either placebo followed by empagliflozin 10 mg/d, or vice versa, using a permuted block randomization method stratified by study site. Randomization was performed by a study statistician. The randomization table was embedded in the electronic data capture system and revealed to each site upon patient enrollment. Prelabeled study drug bottles were dispensed by blinded pharmacists at each site. All participants, investigators, and study staff remained blinded throughout the trial. The appearance and packaging of empagliflozin and placebo tablets were identical.

### Procedures

The study followed a 2-period crossover design, with each treatment period lasting 8 weeks, separated by a 4-week washout phase. Participants visited the clinic every 4 weeks for follow-up assessments, including physical examination, body weight, heart rate, and seated blood pressure measurements (averaged from 2 readings), with adverse events systematically collected at each visit. During each visit, 24-hour urine samples were collected (Supplementary Figure S1). At both the beginning and end of each treatment period, body composition was assessed using bioimpedance analysis, and a FAST PET was performed.^14^ Adherence was evaluated using patient interviews at each visit and by tablet counts at the end of each treatment period. Further methodological details on biomarker analyses and PET are provided in Supplementary Text 1.

### Outcomes

The primary outcome was the change in daily UFV from baseline to week 8 of each treatment period. Daily UFV was self-recorded by participants and calculated as the mean of 5 values from the most recent 7 days, excluding the highest and lowest values. UFV derived from non–glucose-based solutions, such as icodextrin, was excluded from the analysis. Secondary outcomes included changes in 24-hour urine parameters, PET-based metrics (UFV and dialysate sodium, glucose, interleukin-6, and carbohydrate antigen 125concentrations), N-terminal pro-B-type natriuretic peptide (NT-proBNP) levels, body weight, blood pressure, and body composition. To assess the impact on residual kidney function, urinary kidney injury molecule-1 concentrations were measured at the end of each treatment period.

### Sample Size Calculation

Our preliminary data indicated that 5 patients on PD treated with empagliflozin 10 mg/d experienced a mean increase in daily UFV of 90 ml. Notably, a UFV increase of this magnitude has been associated with an 18% reduction in mortality risk in previous studies.^15^ Therefore, we assumed that a 90 ml/d difference in UFV between treatment arms would represent a clinically meaningful effect. In the University of Osaka PD cohort, the SD of UFV changes was calculated as 114 ml. However, for conservative planning, we assumed a higher SD of 150 ml. Although within-subject variability is typically smaller than between-subject variability, we conservatively assumed a 1:1 ratio between them to avoid underestimating variability in the crossover design. Under this assumption, both the within-subject SD and the between-subject SD were set at approximately 106 ml. Using these estimates, we calculated that a sample size of 30 participants would provide 90% power to detect a 90 ml/d difference in UFV between treatment arms, with a 2-sided α level of 0.05. To account for potential dropouts, the target sample size was set at 36.

### Statistical Analysis

Descriptive statistics were used to summarize baseline characteristics and are presented as medians with interquartile ranges. The primary analysis followed a modified intention-to-treat approach, defined here as the full analysis set, which included all randomized participants who received ≥1 dose of study drug and had ≥1 postbaseline assessment. A mixed-effects model for repeated measures was applied to evaluate differences between empagliflozin and placebo, with treatment, period, and treatment-by-period interaction as fixed effects, and individual participants as random effects. An unstructured covariance matrix was specified to model within-subject correlations across treatment periods. For skewed variables, data were log-transformed before modeling. Geometric mean ratios and their 95% CIs were obtained by exponentiating the differences in least-squares means estimated from the mixed-effects model for repeated measures. A prespecified sensitivity analysis using the same model was conducted after excluding data from participants with suspected catheter dysfunction, defined as a drainage volume < 80% of the instilled volume or an instillation time ≥ 20 minutes. In addition, a *post hoc* sensitivity analysis evaluated the change in total daily UFV, including icodextrin-associated ultrafiltration. Prespecified subgroup analyses of the primary outcome were based on age, sex, diabetes status, use of icodextrin-containing PD solution, 24-hour urine volume, and glucose load from dialysate. Peritoneal membrane characteristics were considered, as assessed through the PET, including glucose concentration in dialysate effluent and the dialysate-to-serum creatinine ratio. Secondary outcomes for continuous variables were analyzed using the same mixed-effects model for repeated measures approach as the primary outcome, except for urinary kidney injury molecule-1, for which the absolute value at the end of each treatment period was used as the dependent variable. Adverse events were compared between groups using the safety analysis set, which included all participants who received ≥1 dose of the study drug. Multiplicity was not adjusted, and missing data were not imputed. All statistical analyses were conducted using SAS software (SAS Institute, Cary, NC).

## Results

### Baseline Characteristics

Between December 2023 and May 2024, 41 patients were screened, and 40 were randomized to receive empagliflozin 10 mg/d in period 1 followed by placebo in period 2 (*n* = 20) or the reverse sequence (*n* = 20; Supplementary Figure S2). In the empagliflozin-first group, 1 participant did not initiate treatment, and 3 discontinued during period 1 because of adverse events. In the placebo-first group, 2 did not initiate treatment, and 1 discontinued during washout; 2 further discontinued during period 2 because of adverse events. Ultimately, 37 participants (19 in the empagliflozin-first group, 18 in the placebo-first group) were included in efficacy analyses. Baseline characteristics are presented in Table 1. The median age was 66 years, with 46% female participants, and 27% had diabetes. The median PD duration was 2.8 years. Among 31 participants with baseline 24-hour urine data, the median urine volume was 492 ml, with 19% having < 200 ml/d. Regarding PD prescriptions, 81% were on icodextrin-based solutions. The median daily UFV was 476 ml (mean: 467 ml), and median daily glucose-based PD solution volume was 4500 ml (Supplementary Figure S3). Adherence to study medication, defined as ≥ 80% tablet intake confirmed by tablet counts, was high, with 100% adherence in period 1 and 94% in period 2 (31 of 33 participants).

**Table 1:** Baseline characteristics

Characteristic	Empagliflozin then placebo (*n* = 19)	Placebo then Empagliflozin (*n* = 18)	Overall (*N* = 37)
Age, yrs	65 (56–78)	72 (60–76)	66 (60–76)
Female sex, *n* (%)	10 (53)	7 (39)	17 (46)
Kidney disease^a^			
Chronic glomerulonephritis	4 (21)	2 (11)	6 (16)
Diabetic kidney disease	4 (21)	5 (28)	9 (24)
Nephrosclerosis	5 (26)	3 (17)	8 (22)
Others	7 (37)	8 (44)	15 (41)
Atrial fibrillation, *n* (%)	2 (11)	0 (0)	2 (5)
Diabetes mellitus, *n* (%)	5 (26)	5 (28)	10 (27)
NYHA class, *n* (%)			
Class I	18 (95)	15 (83)	33 (89)
Class II	1 (5)	3 (17)	4 (11)
PD modality, *n* (%)			
APD	9 (45)	6 (35)	15 (41)
CAPD	11 (55)	11 (65)	22 (59)
PD duration, yrs	2.6 (1.3–4.9)	2.8 (1.0–4.4)	2.8 (1.1–4.5)
Body weight, kg	60.4 (50.6–66.2)	57.1 (53.2–63.9)	57.9 (53.1–64.0)
Body mass index, kg/m^2^	22.3 (20.7–24.3)	22.6 (20.7–24.0)	22.4 (20.7–24.0)
SBP, mmHg	138 (124–144)	136 (108–144)	136 (123–144)
DBP, mmHg	82 (67–90)	86 (70–96)	74 (62–86)
LVMI, g/m^2^	92 (88–102)	84 (72–120)	91 (75–104)
LAVI, ml/m^2^	30 (24–45)	47 (41–65)	42 (30–48)
Serum albumin, g/dl	3.2 (2.7–3.7)	3.1 (2.7–3.5)	3.2 (2.7–3.6)
Serum creatinine, mg/dl	10.1 (8.2–13.1)	10.7 (6.6–12.6)	10.7 (7.3–12.6)
NT-proBNP, pg/ml	3105 (1950–9450)	4220 (1269–9700)	3570 (1505–9575)
24-h urine volume, ml	421 (243–620)	566 (274–800)	492 (243–786)
PD solution^a^			
– Icodextrin-based solution, *n* (%)	17 (90)	13 (72)	30 (81)
– Low-glucose concentration (1.35% or 1.36%), *n* (%)	15 (83)	15 (83)	30 (83)
– Intermediate-glucose concentration (2.27% or 2.5%), *n* (%)	10 (56)	6 (33)	16 (44)
– Total volume of glucose-based solution, ml/d	5538 (3100–6000)	4500 (3000–5394)	4500 (3050–6000)
Daily Ultrafiltration volume, ml/d			
Glucose-based solution only	561 (113–800)	435 (228–835)	476 (166–824)
Including icodextrin solution	919 (675–1170)	899 (463–1248)	919 (588–1200)
D/S creatinine ratio	0.73 (0.65–0.81)	0.68 (0.61–0.74)	0.70 (0.62–0.80)
Medication			
ACE inhibitors/ARB/ARNI	18 (95)	14 (78)	32 (86)
Loop diuretics	15 (79)	15 (83)	30 (81)
MRA	5 (26)	4 (22)	9 (24)
Beta-blockers	12 (63)	9 (50)	21 (57)

ACE, angiotensin–converting enzyme; APD, automated peritoneal dialysis; ARB, angiotensin receptor blockers; ARNI, angiotensin receptor–neprilysin inhibitor; CAPD, continuous ambulatory peritoneal dialysis; D/S concentration ratio, dialysate/serum concentration ratio; DBP, diastolic blood pressure; LAVI, left atrial volume index; LVMI, left ventricular mass index; MRA, mineralocorticoid receptor antagonist; NT-proBNP, N-terminal pro-B-type natriuretic peptide; Abbreviations: NYHA, New York Heart Association; PD, peritoneal dialysis; PET, peritoneal equilibration test; SBP, systolic blood pressure.

Data are presented as median (interquartile range) for continuous measures, and n (%) for categorical measures.

a Multiple answers allowed.

### Primary Outcome

**Figure 1:**
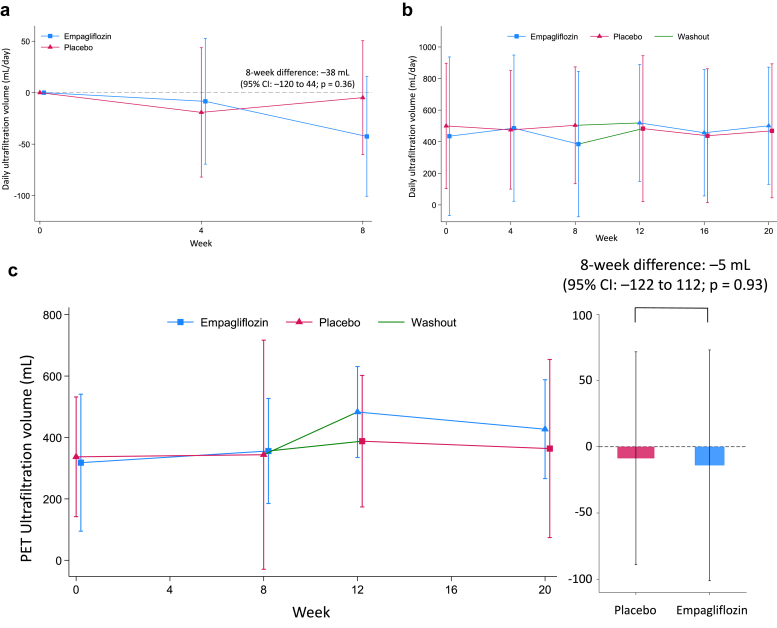

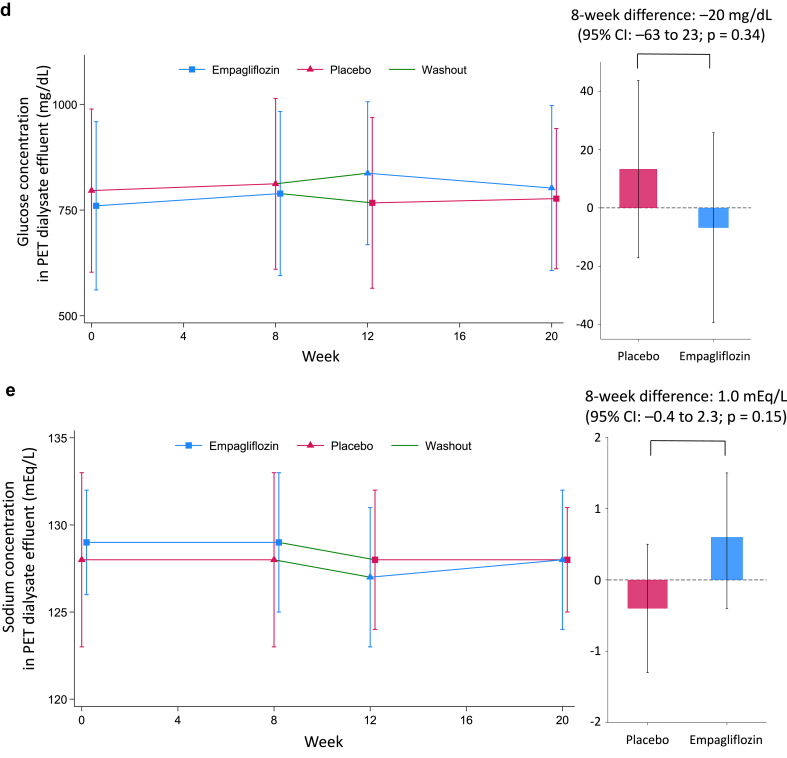
Changes in daily ultrafiltration volume and PET parameters. (a) Estimated mean daily ultrafiltration volume by treatment group (empagliflozin or placebo) at each time point, based on a linear mixed-effects model. Error bars indicate 95% CIs. (b) Daily ultrafiltration volume from glucose-based peritoneal dialysis solution is shown as mean ± SD at each study visit, stratified by treatment sequence (empagliflozin → placebo and placebo → empagliflozin). (c–e) Each parameter is presented as mean ± SD over time by treatment sequence (left panels), and as estimated 8-week treatment differences (empagliflozin minus placebo) with 95% CIs (right panels). (c) Ultrafiltration volume during PET. (d) Glucose concentration of PET dialysate effluent. (e) Sodium concentration of PET dialysate effluent. CI, confidence interval; PET, peritoneal equilibration test.

**Figure 2:**
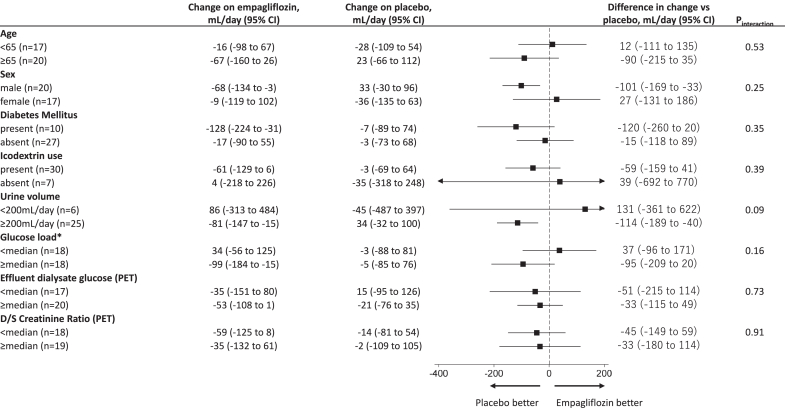
Subgroup analysis of changes in daily ultrafiltration volume with empagliflozin versus placebo. Forest plot displaying subgroup analyses of the difference in daily ultrafiltration volume (ml/d) between empagliflozin and placebo. Each subgroup shows the mean change in ultrafiltration from baseline to the end of each 8-week treatment period, along with the estimated treatment effect and corresponding 95% CIs. A positive value indicates greater ultrafiltration with empagliflozin. ∗The glucose load was defined as the total glucose amount from all glucose-based peritoneal dialysis solutions used at baseline, calculated by multiplying the glucose concentration and the daily volume for each solution and summing the results. CI, confidence interval; D/S, dialysate-to-serum concentration ratio; PET, peritoneal equilibration test.

At the start of placebo treatment, the median daily UFV was 435 ml (mean: 492 ml), changed by −5 ml (95% CI: −60 to 50) after 8 weeks. With empagliflozin, the baseline median UFV was 487 ml (mean: 476 ml), changing by −43 ml (95% CI: −101 to 16). The between-treatment difference in 8-week UFV change was −38 ml (95% CI: −120 to 44; *P* = 0.36; Table 2, Figure 1). A prespecified sensitivity analysis demonstrated a comparable, nonsignificant difference of −38 ml (95% CI: −129 to 54; *P* = 0.40). Similarly, the difference in daily UFV at week 4 did not reach statistical significance (11 ml; 95% CI: −82 to 104; *P* = 0.82; Figure 1). Moreover, a *post hoc* analysis incorporating total UFV, including icodextrin-associated ultrafiltration, revealed no significant treatment effect (−27 ml; 95% CI: −101 to 47; *P* = 0.46). Subgroup analyses showed consistent effects across prespecified categories (Figure 2). The daily volume of glucose-based PD solution, as well as the use of icodextrin, high-concentration glucose solutions, and loop diuretics, remained stable during the study (Supplementary Table S2, Supplementary Figure S4).

**Table 2:** Summary statistics for the primary outcome and secondary outcomes

Parameters	Treatment	Baseline	Week 8	Within-treatment change (95% CI)	Difference in change vs placebo (95% CI)	*P*-value
Primary outcome						
Daily ultrafiltration volume, ml	EMPA	487 (187–842)	393 (156–767)	−43 (−101 to 16)	–38 (−120 to 44)	0.36
	PBO	435 (155–812)	467 (153–777)	−5 (−60 to 50)		
Secondary outcomes						
4-h peritoneal equilibration test (PET) parameters						
Ultrafiltration volume, ml	EMPA	395 (250–555)	427 (278–520)	−14 (−101 to 73)	−5 (−122 to 112)	0.93
	PBO	410 (264–500)	400 (260–520)	−9 (−89 to 72)		
Effluent dialysate glucose, mg/dl	EMPA	816 (693–945)	801 (706–887)	−7 (−39 to 26)	−20 (−63 to 23)	0.34
	PBO	810 (698–924)	794 (733–963)	13 (−17 to 44)		
Effluent dialysate sodium, mEq/l	EMPA	128 (126–130)	129 (126–131)	0.6 (−0.4 to 1.5)	1.0 (−0.4 to 2.3)	0.15
	PBO	128 (126–131)	129 (125–131)	−0.4 (−1.3 to 0.5)		
D/S creatinine ratio	EMPA	0.68 (0.60–0.76)	0.70 (0.60–0.77)	0.01 (−0.02 to 0.03)	0.02 (−0.01 to 0.05)	0.10
	PBO	0.69 (0.62–0.79)	0.68 (0.61–0.75)	−0.02 (−0.04 to 0.00)		
Effluent dialysate IL-6, pg/ml	EMPA	15.6 (9.3–31.9)	17.0 (10.7–34.8)	1.06 (0.86–1.31)^a^	1.11 (0.85–1.45)^a^	0.42
	PBO	16.6 (11.1–31.2)	15.3 (9.7–25.6)	0.96 (0.79–1.16)^a^		
Effluent dialysate CA125, U/ml	EMPA	17.4 (14.2–27.6)	19.1 (13.7–26.6)	1.02 (0.94–1.11)^a^	0.96 (0.86–1.06)^a^	0.38
	PBO	18.6 (12.4–27.7)	19.2 (14.0–27.2)	1.07 (0.99–1.16)^a^		
24-h urine collection parameter						
Urine volume, ml	EMPA	440 (243–764)	430 (284–872)	121 (3–240)	125 (−48 to 299)	0.15
	PBO	402 (220–786)	416 (226–776)	−4 (−112–104)		
Urine glucose, mg	EMPA	199 (61–489)	761 (270–2363)	2198 (528–3868)	2698 (343–5052)	0.03
	PBO	247 (86–514)	199 (51–556)	−499 (−2116 to 1117)		
Urine sodium, mEq	EMPA	32 (15–48)	34 (18–68)	8.9 (0.0 to 17.9)	8.7 (−2.6 to 20.0)	0.13
	PBO	33 (16–48)	28 (14–56)	0.2 (−8.0 to 8.4)		
Urine protein, mg	EMPA	302 (169–521)	215 (88–518)	0.69 (0.47 to 1.02)^a^	0.60 (0.31 to 1.17)^a^	0.13
	PBO	354 (80–775)	402 (143–609)	1.16 (0.79–1.69)^a^		
Creatinine clearance, mL/min^b^	EMPA	1.3 (0.4–3.2)	0.8 (0.4–3.5)	1.03 (0.84–1.27)^a^	1.02 (0.71–1.48)^a^	0.90
	PBO	0.7 (0.4–2.7)	1.2 (0.4–3.2)	1.00 (0.83–1.22)^a^		
Urinary KIM-1, ng/ml	EMPA	NA	0.77 (0.63–1.26)	NA	0.85 (0.71–1.02)^a^	0.08
	PBO	NA	1.11 (0.51–1.32)	NA		
Hemodynamic and fluid balance related parameters						
NT-proBNP, pg/ml	EMPA	3200 (1540–8770)	3505 (1410–5050)	1.02 (0.84–1.23)^a^	0.79 (0.61–1.02)^a^	0.07
	PBO	3630 (1270–8690)	4580 (1900–12100)	1.29 (1.08–1.54)^a^		
Body weight, kg	EMPA	57.5 (51.8–64.2)	57.2 (52.9–64.6)	−0.6 (−1.1 to 0.0)	−0.6 (−1.4 to 0.2)	0.12
	PBO	58.1 (53.0–66.2)	58.8 (52.6–67.2)	0.0 (−0.5 to 0.6)		
Systolic blood pressure, mmHg	EMPA	131 (123–142)	131 (121–146)	0.7 (−5.8 to 7.1)	1.5 (−7.8 to 10.9)	0.74
	PBO	136 (126–150)	134 (122–145)	−0.9 (−7.1 to 5.4)		
Diastolic blood pressure, mmHg	EMPA	83 (72–94)	82 (68–92)	−2.0 (−6.0 to 1.9)	−4.2 (−10.1 to 1.7)	0.16
	PBO	82 (66–89)	82 (70–91)	2.2 (−1.7 to 6.0)		
Intracellular fluid volume, L^c^	EMPA	17.9 (13.1–20.3)	17.6 (15.6–20.3)	0.1 (−0.8 to 1.0)	0.1 (−1.1 to 1.4)	0.85
	PBO	18.3 (14.4–19.7)	18.8 (14.9–20.3)	0.0 (−0.9 to 0.9)		
Extracellular fluid volume, L^c^	EMPA	14.7 (12.3–18.2)	15.2 (12.3–18.2)	−0.3 (−0.9 to 0.2)	−0.5 (−1.1 to 0.2)	0.14
	PBO	14.6 (13.1–16.6)	14.4 (13.1–18.6)	0.2 (−0.4 to 0.7)		

Descriptive statistics are presented as median (interquartile range) to summarize the distribution of continuous variables. Treatment effects are estimated as the difference between empagliflozin and placebo using linear mixed-effects models, which included treatment, period, and treatment-by-period interaction as fixed effects, and individual participants as random effects. As these models estimate mean differences, the reported descriptive statistics are provided for illustrative purposes only and should not be interpreted as direct estimates of treatment effect.

CA125, carbohydrate antigen 125; CI, confidence interval; D/S concentration ratio, dialysate/serum concentration ratio; EMPA, empagliflozin; IL-6, interleukin-6; KIM-1, kidney injury molecule 1; NT-proBNP, N-terminal pro-B-type natriuretic peptide; PBO, placebo.

a Estimated values are presented as the geometric mean ratios.

b Calculated as the average of renal urea clearance and creatinine clearance based on 24-hour urine collection and corresponding serum values.

c Body composition was assessed using 2 distinct bioimpedance spectroscopy devices across study sites. Detailed methodology and device specifications, including changes in intracellular and extracellular fluid volumes assessed by bioimpedance devices, are provided in the Supplementary Table 4.

### Secondary Outcomes

#### PET Parameters

Empagliflozin treatment for 8 weeks did not affect PET-based UFV (mean difference −5 ml; 95% CI: −122 to 112; *P* = 0.93; Table 2, Figure 1). No significant changes were observed in dialysate glucose (−20 mg/dl; 95% CI: −63 to 23; *P* = 0.34) or sodium levels (1.0 mEq/l; 95% CI: −0.4 to 2.3; *P* = 0.15). Inflammatory and mesothelial biomarkers also remained unchanged, with interleukin-6 ratio of 1.11 (95% CI: 0.85–1.45; *P* = 0.42) and carbohydrate antigen 125 ratio of 0.96 (95% CI: 0.86–1.06; *P* = 0.38).

### 24-hour Urine Collection Parameter

Empagliflozin significantly increased urinary glucose excretion versus placebo (mean difference 2698 mg; 95% CI: 343–5,052; *P* = 0.03; Table 2, Supplementary Figure S5), but had no significant effect on urine volume (125 ml; 95% CI: −48 to 299; *P* = 0.15), sodium excretion (8.7 mEq; 95% CI: −2.6 to 20.0; *P* = 0.13), or creatinine clearance (ratio: 1.02; 95% CI: 0.71–1.48; *P* = 0.90). There was a nonsignificant trend toward lower urine protein excretion (ratio: 0.60; 95% CI: 0.31–1.17; *P* = 0.13) and urinary kidney injury molecule-1 (ratio: 0.85; 95% CI: 0.71–1.02; *P* = 0.08). *Post hoc* subgroup analysis by baseline urine volume (≤ 200 vs. > 200 ml) showed consistent effects across subgroups (Supplementary Table S3).

### Hemodynamic and Fluid Balance–related Parameters

Empagliflozin treatment showed a nonsignificant 21% reduction in NT-proBNP levels versus placebo (ratio: 0.79; 95% CI: 0.61–1.02; *P* = 0.07; Table 2). Body weight and extracellular fluid volume tended to be lower, with mean reductions of 0.6 kg (95% CI: −1.4 to 0.2; *P* = 0.12) and 0.5 L (95% CI: −1.1 to 0.2; *P* = 0.14), respectively. No significant differences were seen in blood pressure.

### Adverse Events

Overall, adverse events were reported in 17 of 36 participants (47%) during empagliflozin treatment and in 10 of 34 (29%) during placebo treatment (Table 3), with no specific event category accounting for the difference (Supplementary Table S5). Serious adverse events occurred in 4 patients (11%) on empagliflozin and 2 (6%) on placebo; peritonitis was the most common, with 3 cases overall. No deaths, diabetic ketoacidosis, hypoglycemia, amputations, genital infections, or urinary tract infections were reported. The reasons for treatment discontinuation are summarized in Table S6. Importantly, most adverse events that led to treatment discontinuation were judged to be unrelated to the study drug.

**Table 3:** Number of patients with adverse events by treatment periods

Adverse event	Placebo (*n* = 34)	Empagliflozin (*n* = 36)
Any adverse event	10 (29)	17 (47)
Any serious adverse event^a^	2 (6)	4 (11)
Peritonitis	1 (3)	2 (6)
Peritoneal catheter tunnel infections	1 (3)	0
Acute pancreatitis	1 (3)	0
Uterine cancer	0	1 (3)
Worsening heart failure	1 (3)	0
Death	0	0
Adverse event of special interest		
Amputations	0	0
Diabetic ketoacidosis	0	0
Hypoglycemia	0	0
Hypotension	1 (3)	0
Genital infection	0	0
Urinary tract infection	0	0

Data represent the number of patients (%) who experienced at least one adverse event of the specified type, including event types with zero occurrences. Adverse events observed during the washout period were categorized and recorded as Period 1 adverse events. The safety analysis set included all patients who received at least one dose of the trial drug. Adverse events were coded using the Medical Dictionary for Regulatory Activities (MedDRA), version 27.1.

a One patient experienced multiple serious adverse events, resulting in a total event count that exceeded the number of affected patients.

## Discussion

This randomized crossover trial is the first to evaluate the efficacy and safety of an SGLT2 inhibitor in the dialysis population. We found that 8 weeks of empagliflozin did not significantly increase daily UFV from glucose-based dialysate, as confirmed by PET measures of UFV and dialysate glucose concentration. Importantly, although efficacy on UFV was not demonstrated, we observed a numerically higher incidence of adverse events with empagliflozin treatment (47% vs. 29%), highlighting the need for careful safety evaluation of SGLT2 inhibitors in this population.

Previous data on SGLT2 inhibitors and UFV in PD have been limited and conflicting.^8^, ^9^, ^10^, ^11^ For example, a prospective observational study of 50 diabetic patients on PD reported increased UFV after 6 months of dapagliflozin.^11^ Another study found higher UFV among patients who continued SGLT2 inhibitors after starting PD than in those who discontinued.^8^ Conversely, a Spanish retrospective study (*n* = 16) and a single-arm trial (*n* = 20) found no significant changes in UFV or glucose absorption.^9^^,^^10^ Consistent with these latter findings, our randomized trial demonstrated no significant impact of empagliflozin on daily UFV, PET-based UFV, or dialysate glucose concentration.

Several factors may explain the lack of UFV increase by empagliflozin. First, systemic administration may not achieve therapeutic intraperitoneal concentration sufficiently to inhibit local glucose uptake. Second, SGLT2 may be less important for peritoneal glucose uptake than other transporters, such as SGLT1 and GLUT isoforms.^16^, ^17^, ^18^ Third, previous studies showing increased UFV used longer treatment durations (≥ 6 months),^8^^,^^11^ suggesting that membrane-related benefits might emerge over time. Supporting this, animal studies have demonstrated antifibrotic and antiangiogenic effects of SGLT2 inhibitors, which could preserve membrane integrity and sustain UF by preventing long-term structural damage.^6^^,^^7^^,^^19^ These findings underscore the need to investigate whether SGLT2 inhibitors exert structural effects on the peritoneal membrane and whether such effects, if any, can translate into improved fluid management and clinical outcomes in PD patients.

Residual kidney function is a key determinant of outcomes in patients on PD.^20^ SGLT2 inhibitors exhibit renoprotective effects down to estimated glomerular filtration rate levels of ∼20 ml/min per 1.73 m^2^^21^^,^^22^ and may help maintain residual kidney function in patients on PD. In our study, 8 weeks of empagliflozin had no significant effect on creatinine clearance—calculated as the mean of urinary urea and creatinine clearances—or urine volume. Although empagliflozin modestly increased urinary glucose excretion (∼2.7 g/d), this was substantially lower than levels observed in nondialysis patients,^23^ suggesting minimal osmotic diuresis and consistent urine output.^24^ In addition, the diuretic effect of glucosuria may be limited in the context of very low glomerular filtration rate. Moreover, even in nondialysis populations, glucose-induced diuresis is often mitigated within 24 to 48 hours by neuroendocrine compensation, including increased antidiuretic hormone and aquaporin-2 expression.^24^ Notably, empagliflozin reduced urinary kidney injury molecule-1 by 15% and proteinuria by 40%, though these changes were not statistically significant. These favorable trends, similar to effects seen in nondialysis patients,^25^^,^^26^ merit further investigation in larger, long-term trials.

Interestingly, empagliflozin treatment was associated with favorable trends, including reductions in NT-proBNP by 21%, body weight by 0.6 kg, and extracellular fluid volume by 0.5 L, despite no significant changes in UFV or urine volume. Similar findings were reported in a substudy of EMPA-KIDNEY, where empagliflozin 10 mg reduced extracellular water by 0.52 L (95% CI: −0.72 to −0.32) and body weight by 0.9 kg (95% CI: −1.4 to −0.3) over 2 months compared with placebo.^27^ Although the mechanisms underlying these changes remain uncertain, reductions in NT-proBNP may reflect effects beyond fluid removal, including direct myocardial actions via inhibition of the sodium–hydrogen exchanger,^28^ modulation of intracellular ion homeostasis,^29^ attenuation of oxidative stress and inflammation,^30^ or regulation of autophagy,^31^^,^^32^ as suggested by preclinical studies.

The tendency toward a higher incidence of adverse events during empagliflozin treatment compared with placebo (47% vs. 29%) is noteworthy. This numerical difference did not appear to be driven by any specific category of events. Given the limited sample size and the exploratory nature of the study, these findings should be interpreted with caution. Nonetheless, they underscore the importance of close monitoring of safety outcomes, particularly in vulnerable populations such as patients on PD.

The strength of our study lies in its rigorous randomized, double-blind, placebo-controlled, crossover design, which minimizes bias, enhances reliability, and reduces interindividual variability. However, several limitations merit consideration. First, although the sample size was prospectively calculated to detect a 90 ml/d difference in UFV with sufficient statistical power, the absence of a significant effect should not be interpreted as conclusive evidence of lack of efficacy, especially given the relatively small sample size. Second, the patient cohort reflects typical Japanese PD practice, characterized by infrequent use of high-concentration glucose solutions (e.g., 3.86%) and lower prescribed dialysate volumes compared with practices in other countries.^33^ Consequently, the cumulative glucose load was limited, potentially attenuating the observable effects of SGLT2 inhibitors. However, no favorable trends were evident even among patients with higher glucose exposure, suggesting that the intervention conferred no measurable benefit across both low and high glucose load contexts. Third, the biomarker criteria used for entry (BNP ≥ 40 pg/ml or NT-proBNP ≥ 400 pg/ml) represent relatively low thresholds for patients on dialysis and may have allowed inclusion of participants with less advanced disease, unlike conventional heart failure trials that typically include patients with more advanced symptoms. Lastly, the follow-up duration of 8 weeks per treatment period was relatively short, potentially restricting our ability to observe longer-term effects on peritoneal function and fluid balance.

In this randomized, placebo-controlled trial of patients on PD, empagliflozin did not improve UFV or reduce peritoneal glucose absorption. Although no enhancement in fluid removal was observed, favorable trends in biomarkers of fluid status, tubular injury, and proteinuria were noted. Given the limitations of sample size, glucose exposure, and follow-up duration, longer-term studies are warranted to assess potential delayed or indirect benefits—such as preservation of peritoneal membrane integrity, maintenance of residual kidney function, or cardiovascular protection—in this vulnerable population.

## Appendix

### List of the EMPOWERED Investigators

Masayoshi Kukida, Fumikazu Kondo, Satoru Shichijo, Yohei Morita, Ken-Ichi Miyoshi, and Osamu Yamaguchi, Department of Cardiology, Pulmonology, Hypertension and Nephrology, Ehime University Graduate School of Medicine, Toon, Ehime, Japan.

## Disclosure

YNa reports employment with Nippon Boehringer Ingelheim Co., Ltd. YNi reports employment with Nippon Boehringer Ingelheim Co., Ltd. YD reports honoraria with Mitsubishi Tanabe Pharma Corporation, and research funding from Nippon Boehringer Ingelheim Co., Ltd. and Eli Lilly Japan K.K. TA reports honoraria from AstraZeneca K.K. TH reports Speakers Bureau fees from AstraZeneca plc, Ono Pharmaceutical Co., Ltd., Nippon Boehringer Ingelheim Co., Ltd., Mitsubishi Tanabe Pharma Corporation, Astellas Pharma Inc., Taisho Pharmaceutical Co., Ltd., Kowa Co., Ltd.; research funding from Astellas Pharma Inc.; and provision of study materials from Kowa Co., Ltd. FY reports grants from AstraZeneca K.K., Ono Pharmaceutical Co., Ltd., and Kowa Co., Ltd.; and honoraria from AstraZeneca plc, Mitsubishi Tanabe Pharma Corporation, and Astellas Pharma Inc. YI reports Speakers Bureau fees from AstraZeneca plc, Mitsubishi Tanabe Pharma Corporation, Kissei Pharmaceutical Co., Ltd., Astellas Pharma Inc., and Nippon Boehringer Ingelheim Co., Ltd.; consultancy with Nippon Boehringer Ingelheim Co., Ltd.; and honoraria from Nippon Boehringer Ingelheim Co., Ltd. All the other authors declared no competing interests.

## Supplementary Material

Supplementary File (PDF) **Supplementary References. Table S1.** Inclusion and exclusion criteria. **Table S2.** Changes in concomitant use of diuretics, MRA, and PD solutions during empagliflozin and placebo treatment periods. **Table S3.** Effects of 8-week treatment on 24-hour urine parameters, stratified by baseline urine volume. **Table S4.** Changes in intracellular and extracellular fluid volumes measured by 2 different bioimpedance devices. **Table S5.** Adverse events by severity, system organ class, and preferred term. **Table S6.** Adverse events leading to discontinuation from trial. **Figure S1.** Study design of a randomized, placebo-controlled, crossover trial. **Figure S2.** Participant flow diagram. **Figure S3.** Baseline daily ultrafiltration volume by treatment sequence. **Figure S4.** Changes in daily glucose-based peritoneal dialysis solution volume over time. **Figure S5.** Changes in 24-hour urine parameters over time. Supplementary Text 1. Protocols for biomarker measurement, bioimpedance analysis, and peritoneal equilibration testing. CONSORT Checklist. Protocol.
