# Supplementary material for: Empagliflozin and Ultrafiltration Volume in Patients Undergoing Peritoneal Dialysis
Source: Kidney Int Rep. 2025 Oct 10;11(1):141–51. doi: 10.1016/j.ekir.2025.09.049 (PMC12799575; doi:10.1016/j.ekir.2025.09.049)
Supplement: Supplementary File (PDF) — Supplementary References. Table S1. Inclusion and exclusion criteria. Table S2. Changes in concomitant use of diuretics, MRA, and PD solutions during empagliflozin and placebo treatment periods. Table S3. Effects of 8-week treatment on 24-hour urine parameters, stratified by baseline urine volume. Table S4. Changes in intracellular and extracellular fluid volumes measured by 2 different bioimpedance devices. Table S5. Adverse events by severity, system organ class, and preferred term. Table S6. Adverse events leading to discontinuation from trial. Figure S1. Study design of a randomized, placebo-controlled, crossover trial. Figure S2. Participant flow diagram. Figure S3. Baseline daily ultrafiltration volume by treatment sequence. Figure S4. Changes in daily glucose-based peritoneal dialysis solution volume over time. Figure S5. Changes in 24-hour urine parameters over time. Supplementary Text 1. Protocols for biomarker measurement, bioimpedance analysis, and peritoneal equilibration testing. CONSORT Checklist. Protocol. [file mmc1.pdf]

Supplementary materials

Table of Contents:

Supplementary Table 1. Inclusion and exclusion criteria

Supplementary Table 2. Changes in Concomitant Use of Diuretics, MRA, and PD Solutions During Empagliflozin and Placebo Treatment Periods

Supplementary Table 3. Effects of 8-Week Treatment on 24-Hour Urine Parameters, Stratified by Baseline Urine Volume

Supplementary Table 4. Changes in Intracellular and Extracellular Fluid Volumes Measured by Two Different Bioimpedance Devices

Supplementary Table 5. Adverse Events by Severity, System Organ Class, and Preferred Term

Supplementary Table 6. Adverse Events leading to discontinuation from trial

Supplementary Figure 1. Study design of a randomized, placebo-controlled, crossover trial

Supplementary Figure 2. Participant flow diagram

Supplementary Figure 3. Baseline daily ultrafiltration volume by treatment sequence

Supplementary Figure 4. Changes in daily glucose-based peritoneal dialysis solution volume over time

Supplementary Figure 5. Changes in 24-hour urine parameters over time.

Supplement 1. Protocols for biomarker measurement, bioimpedance analysis, and peritoneal equilibration testing

Supplementary References

CONSORT 2025 checklist

Protocol

Supplementary Table 1. Inclusion and exclusion criteria

**Inclusion criteria**	(1) Age ≥18 and ≤90 years<br>(2) BNP ≥40 pg/mL, NT-proBNP ≥400 pg/mL, structural heart disease (left atrial enlargement and/or left ventricular hypertrophy), elevated filling pressures, or a history of hospitalization for heart failure*<br>(3) Standard medical therapy for heart failure (at least one of the following: loop diuretics, ACEIs, ARBs, ARNIs, beta-blockers, or MRAs)<br>(4) PD vintage ≥ 3 months<br>(5) Glucose-based PD solution use ≥3 L/day<br>(6) Voluntary participation with written informed consent
**Exclusion criteria**	(1) Treatment with SGLT2 inhibitors within 6 months before enrollment<br>(2) Individuals who are not expected to survive more than 1 year after enrollment<br>(3) On a hybrid therapy comprising peritoneal dialysis and hemodialysis<br>(4) Individuals who have or have had peritonitis within the past 2 months<br>(5) Women who are pregnant or nursing<br>(6) Active infections<br>(7) Individuals who participate in clinical studies (trials and research) involving other interventions<br>(8) Individuals disqualified from participation in the study by the investigator or sub-investigator for any other reasons

*NT–proBNP must be used to confirm eligibility for participants receiving ARNIs

Abbreviations: BNP, brain natriuretic peptide; NT–proBNP, N-terminal pro-brain natriuretic peptide; ACEIs, angiotensin-converting enzyme inhibitors; ARBs, angiotensin receptor blockers; ARNIs, angiotensin receptor neprilysin inhibitors; MRAs, mineralocorticoid receptor antagonists; PD, peritoneal dialysis; SGLT2, Sodium-Glucose Cotransporter 2

*Adapted from Doi Y, et al. Clin Exp Nephrol. 2024;28(7):629–635.

Supplementary Table 2. Changes in Concomitant Use of Diuretics, MRA, and PD Solutions During Empagliflozin and Placebo Treatment Periods

		**Empagliflozin**			**Placebo**		
		Reduced	Unchanged	Increased	Reduced	Unchanged	Increased
Drug*	Class						
− Furosemide	Loop diuretic	0	19	0	0	21	1
− Azosemide	Loop diuretic	0	13	0	1	13	0
− Trichlormethiazide	Thiazide-like diuretic	0	8	0	0	8	0
− Indapamide	Thiazide-like diuretic	0	4	0	0	4	0
− Tolvaptan	V2 receptor antagonist	0	6	0	0	6	0
− Spironolactone	Mineralocorticoid receptor antagonist	0	9	0	0	9	0
PD solution							
− Icodextrin†		1	22	2	1	22	0
− Intermediate-glucose concentration (2.27% or 2.5%)‡		2	12	2	3	16	0

No patients received high-glucose concentration (3.86% or 4.00%) solutions during the study.

*Dosage changes were assessed between baseline and week 8 of each treatment period.

†Data represent 30 patients who used icodextrin solutions at any point during the study. Changes were evaluated at baseline and at week 8 of each treatment period.

"Reduced" indicates either discontinuation or a decrease in the volume of icodextrin solution.

"Increased" indicates either initiation or an increase in the volume of icodextrin solution.

Data represent 20 patients who used intermediate-glucose concentration (2.27% or 2.5%) peritoneal dialysis solutions at any point during the study.

‡Changes were evaluated at baseline and at week 8 of each treatment period.

"Reduced" indicates either discontinuation or a decrease in the volume of intermediate-glucose solution.

"Increased" indicates either initiation or an increase in the volume of intermediate-glucose solution.

Abbreviations: MRA, mineralocorticoid receptor antagonist; PD, peritoneal dialysis.

Supplementary Table 3. Effects of 8-Week Treatment on 24-Hour Urine Parameters, Stratified by Baseline Urine Volume

Parameters	Changes on empagliflozin (95% CI)	Changes on placebo (95% CI)	Difference in change vs placebo (95% CI)	$P_{interaction}$
**Urine volume, mL**				
<200mL/day (n=6)	1 (-56 to 57)	-13 (-70 to 45)	13 (-101 to 128)	0.60
≥200mL/day (n=25)	154 (8 to 301)	-5 (-139 to 129)	159 (-58 to 376)	
**Urine glucose excretion, mg**				
<200mL/day (n=6)	58 (-5 to 121)	-19 (-96 to 58)	77 (-40 to 194)	0.56
≥200mL/day (n=25)	2,598 (585 to 4611)	-605 (-2734 to 1524)	3,203 (222 to 6185)	
**Urine sodium excretion, mEq**				
<200mL/day (n=6)	-0.9 (-5.6 to 3.8)	-1.0 (-5.9 to 3.9)	0.1 (-10.0 to 10.2)	0.54
≥200mL/day (n=25)	11.7 (0.6 to 22.7)	0.0 (-10.2 to 10.2)	11.7 (-2.1 to 25.5)	
**Urine protein excretion, mg***				
<200mL/day (n=6)	0.77 (0.29 to 2.10)	0.99 (0.30 to 3.33)	0.78 (0.13 to 4.74)	0.78
≥200mL/day (n=25)	0.66 (0.42 to 1.03)	1.17 (0.72 to 1.91)	0.56 (0.24 to 1.29)	

The treatment effect is indicated by the estimated difference in the outcome (estimate [95% CI]: empagliflozin minus placebo), derived from linear mixed-effects models. Models included treatment, period, and treatment-by-period interaction as fixed effects, with individuals as random effects.

*Estimated values are presented as the geometric mean ratios.

Abbreviations: CI, confidence

Supplementary Table 4. Changes in Intracellular and Extracellular Fluid Volumes Measured by Two Different Bioimpedance Devices

Parameters	Treatment	Baseline	Week 8	Change from baseline to Week 8	Difference in change vs placebo (95% CI)
**Intracellular fluid volume, L**					0.1 (-1.1 to 1.4)*
BCM (n=26)	Empagliflozin	16.7 (12.9–19.5)	16.7 (15.2–20.5)	0.2 (-1.5–2.2)	
	Placebo	17.4 (14.1–19.4)	17.5 (13.7–19.8)	-0.2 (-0.8–1.1)	
InBody (n=9)	Empagliflozin	20.1 (17.9–20.6)	19.6 (17.4–20.3)	0.1 (-0.9–0.2)	
	Placebo	19.5 (18.0–21.0)	20.1 (18.2–20.6)	0.2 (-0.4–0.4)	
**Extracellular fluid volume, L†**					-0.5 (-1.2 to 0.2)*
BCM (n=25)	Empagliflozin	15.8 (12.9–18.3)	16.3 (13.0–18.2)	-0.1 (-0.9–0.7)	
	Placebo	15.6 (13.9–17.8)	16.3 (13.2–19.2)	0.1 (-1.4–1.1)	
InBody (n=9)	Empagliflozin	13.5 (11.9–14.0)	13.6 (11.3–14.1)	0.1 (-0.8–0.2)	
	Placebo	13.4 (12.2–13.7)	13.8 (12.0–14.1)	0.2 (-0.4–0.3)	

Summary statistics are shown as median (interquartile range) for descriptive purposes. For inferential purposes, treatment effects were estimated as the difference in outcomes (empagliflozin–placebo) using linear mixed-effects models. The models included treatment, period, and their interaction as fixed effects, and individuals as random effects, and device type (BCM vs. InBody) as a fixed effect to adjust for measurement differences across sites.

*The fixed effect of device type (BCM vs. InBody) was not statistically significant in the analysis of intracellular fluid volume (p=0.65) or extracellular fluid volume (p=0.70).

†During the analysis, a patient receiving empagliflozin was found to have a 20.9 L reduction in extracellular fluid volume. Review of the medical record confirmed this to be a data entry error; the actual reduction was 2.8 L. As the data lock had already occurred, the erroneous data point was excluded from the analysis. If the outlier had been included, the estimated change would have been –1.1 L (95% CI, –2.5 to 0.3; p=0.11).

Abbreviations: CI, confidence

Body composition assessment

Two different bioimpedance devices were used across study sites to assess body composition in peritoneal dialysis patients: the Body Composition Monitor (BCM; Fresenius Medical Care) and InBody devices (InBody S10 and InBody 720; InBody Co., Ltd.). Specifically, BCM was used at Osaka University Hospital and Matsuyama Red Cross Hospital, while InBody devices were used at the National Cerebral and Cardiovascular Center and Osaka General Medical Center. The BCM uses bioimpedance spectroscopy (BIS) over a wide frequency range (5 kHz to 1 MHz) and applies a physiological model to separately estimate extracellular water (ECW) and intracellular water (ICW), validated specifically in dialysis populations[S1]. In contrast, InBody devices employ multi-frequency bioimpedance analysis (MF-BIA) at discrete frequencies and use empirical regression equations derived from healthy individuals[S2]. In addition, BCM measures patients in the supine position, while InBody measurements are taken in an upright or sitting posture, which may influence fluid distribution and thereby ECW and ICW values.

Supplementary Table 5. Adverse Events by Severity, System Organ Class, and Preferred Term

		Empagliflozin (n=36)						Placebo (n=34)					
		Mild		Moderate		Severe		Mild		Moderate		Severe	
System Organ Class	Preferred Term	Cases	n (%)	Cases	n (%)	Cases	n (%)	Cases	n (%)	Cases	n (%)	Cases	n (%)
All adverse events		20	13 (36.1)	1	1 (2.8)	3	3 (8.3)	12	8 (23.5)	3	2 (5.9)	0	0 (0.0)
Infections and infestations		7	5 (13.9)	0	0 (0.0)	2	2 (5.6)	2	2 (5.9)	2	2 (5.9)	0	0 (0.0)
	Peritonitis	0	0 (0.0)	0	0 (0.0)	2	2 (5.6)	0	0 (0.0)	1	1 (2.9)	0	0 (0.0)
	Device related infection	2	2 (5.6)	0	0 (0.0)	0	0 (0.0)	0	0 (0.0)	1	1 (2.9)	0	0 (0.0)
	COVID-19	1	1 (2.8)	0	0 (0.0)	0	0 (0.0)	1	1 (2.9)	0	0 (0.0)	0	0 (0.0)
	Gingivitis	1	1 (2.8)	0	0 (0.0)	0	0 (0.0)	0	0 (0.0)	0	0 (0.0)	0	0 (0.0)
	Herpes zoster	1	1 (2.8)	0	0 (0.0)	0	0 (0.0)	0	0 (0.0)	0	0 (0.0)	0	0 (0.0)
	Nasopharyngitis	0	0 (0.0)	0	0 (0.0)	0	0 (0.0)	1	1 (2.9)	0	0 (0.0)	0	0 (0.0)
	Paronychia	1	1 (2.8)	0	0 (0.0)	0	0 (0.0)	0	0 (0.0)	0	0 (0.0)	0	0 (0.0)
	Enteritis infectious	1	1 (2.8)	0	0 (0.0)	0	0 (0.0)	0	0 (0.0)	0	0 (0.0)	0	0 (0.0)
Neoplasms benign, malignant and unspecified (including cysts and polyps)		0	0 (0.0)	1	1 (2.8)	0	0 (0.0)	0	0 (0.0)	0	0 (0.0)	0	0 (0.0)
	Uterine cancer	0	0 (0.0)	1	1 (2.8)	0	0 (0.0)	0	0 (0.0)	0	0 (0.0)	0	0 (0.0)
Blood and lymphatic system disorders		3	3 (8.3)	0	0 (0.0)	0	0 (0.0)	2	2 (5.9)	0	0 (0.0)	0	0 (0.0)

	Anaemia	3	3 (8.3)	0	0 (0.0)	0	0 (0.0)	2	2 (5.9)	0	0 (0.0)	0	0 (0.0)
Metabolism and nutrition disorders		1	1 (2.8)	0	0 (0.0)	0	0 (0.0)	0	0 (0.0)	0	0 (0.0)	0	0 (0.0)
	Diabetes mellitus	1	1 (2.8)	0	0 (0.0)	0	0 (0.0)	0	0 (0.0)	0	0 (0.0)	0	0 (0.0)
Cardiac disorders		0	0 (0.0)	0	0 (0.0)	1	1 (2.8)	1	1 (2.9)	0	0 (0.0)	0	0 (0.0)
	Cardiac failure	0	0 (0.0)	0	0 (0.0)	1	1 (2.8)	1	1 (2.9)	0	0 (0.0)	0	0 (0.0)
Vascular disorders		0	0 (0.0)	0	0 (0.0)	0	0 (0.0)	1	1 (2.9)	0	0 (0.0)	0	0 (0.0)
	Hypotension	0	0 (0.0)	0	0 (0.0)	0	0 (0.0)	1	1 (2.9)	0	0 (0.0)	0	0 (0.0)
Respiratory, thoracic and mediastinal disorders		0	0 (0.0)	0	0 (0.0)	0	0 (0.0)	1	1 (2.9)	0	0 (0.0)	0	0 (0.0)
	Asthma	0	0 (0.0)	0	0 (0.0)	0	0 (0.0)	1	1 (2.9)	0	0 (0.0)	0	0 (0.0)
Gastrointestinal disorders		4	4 (11.1)	0	0 (0.0)	0	0 (0.0)	1	1 (2.9)	1	1 (2.9)	0	0 (0.0)
	Diarrhoea	1	1 (2.8)	0	0 (0.0)	0	0 (0.0)	1	1 (2.9)	0	0 (0.0)	0	0 (0.0)
	Abdominal pain	1	1 (2.8)	0	0 (0.0)	0	0 (0.0)	0	0 (0.0)	0	0 (0.0)	0	0 (0.0)
	Dyspepsia	1	1 (2.8)	0	0 (0.0)	0	0 (0.0)	0	0 (0.0)	0	0 (0.0)	0	0 (0.0)
	Pancreatitis acute	0	0 (0.0)	0	0 (0.0)	0	0 (0.0)	0	0 (0.0)	1	1 (2.9)	0	0 (0.0)
	Bloody peritoneal effluent	1	1 (2.8)	0	0 (0.0)	0	0 (0.0)	0	0 (0.0)	0	0 (0.0)	0	0 (0.0)
Skin and subcutaneous tissue disorders		2	2 (5.6)	0	0 (0.0)	0	0 (0.0)	1	1 (2.9)	0	0 (0.0)	0	0 (0.0)
	Dermal cyst	1	1 (2.8)	0	0 (0.0)	0	0 (0.0)	0	0 (0.0)	0	0 (0.0)	0	0 (0.0)

	Nail dystrophy	0	0 (0.0)	0	0 (0.0)	0	0 (0.0)	1	1 (2.9)	0	0 (0.0)	0	0 (0.0)
	Pruritus	1	1 (2.8)	0	0 (0.0)	0	0 (0.0)	0	0 (0.0)	0	0 (0.0)	0	0 (0.0)
Musculoskeletal and connective tissue disorders		1	1 (2.8)	0	0 (0.0)	0	0 (0.0)	1	1 (2.9)	0	0 (0.0)	0	0 (0.0)
	Back pain	1	1 (2.8)	0	0 (0.0)	0	0 (0.0)	0	0 (0.0)	0	0 (0.0)	0	0 (0.0)
	Osteoarthritis	0	0 (0.0)	0	0 (0.0)	0	0 (0.0)	1	1 (2.9)	0	0 (0.0)	0	0 (0.0)
General disorders and administration site conditions		1	1 (2.8)	0	0 (0.0)	0	0 (0.0)	1	1 (2.9)	0	0 (0.0)	0	0 (0.0)
	Oedema peripheral	1	1 (2.8)	0	0 (0.0)	0	0 (0.0)	1	1 (2.9)	0	0 (0.0)	0	0 (0.0)
Investigations		1	1 (2.8)	0	0 (0.0)	0	0 (0.0)	0	0 (0.0)	0	0 (0.0)	0	0 (0.0)
	Blood parathyroid hormone increased	1	1 (2.8)	0	0 (0.0)	0	0 (0.0)	0	0 (0.0)	0	0 (0.0)	0	0 (0.0)
Injury, poisoning and procedural complications		0	0 (0.0)	0	0 (0.0)	0	0 (0.0)	1	1 (2.9)	0	0 (0.0)	0	0 (0.0)
	Thermal burn	0	0 (0.0)	0	0 (0.0)	0	0 (0.0)	1	1 (2.9)	0	0 (0.0)	0	0 (0.0)

Supplementary Table 6. Adverse Events leading to discontinuation from trial

Period	Treatment	Reason for Discontinuation	Related Adverse Event (Preferred Term)	Related to Study Drug
Period 1	Empagliflozin	Adverse event	Dyspepsia	Yes
Period 1	Empagliflozin	Adverse event	Pruritus	Yes
Period 1	Empagliflozin	Adverse event (not related)	Uterine cancer	No
Period 2	Empagliflozin	Adverse event (not related)	Peritonitis	No
Period 2	Empagliflozin	Adverse event (not related)	Peritonitis	No
Washout	Placebo	Adverse event (not related)	Cardiac failure	No

Note: Relationship between each adverse event and the study drug was assessed by the site investigator according to clinical judgment.

Supplementary Figure 1. Study design of a randomized, placebo-controlled, crossover trial.

A total of 40 patients undergoing peritoneal dialysis were randomized in a 1:1 ratio to receive either empagliflozin or placebo during the first 8-week intervention period. This was followed by a 4-week washout phase, after which participants crossed over to the alternate treatment for a second 8-week intervention period. Study visits were conducted at weeks 0, 4, 8, 12, 16, and 20 for clinical and laboratory assessments.

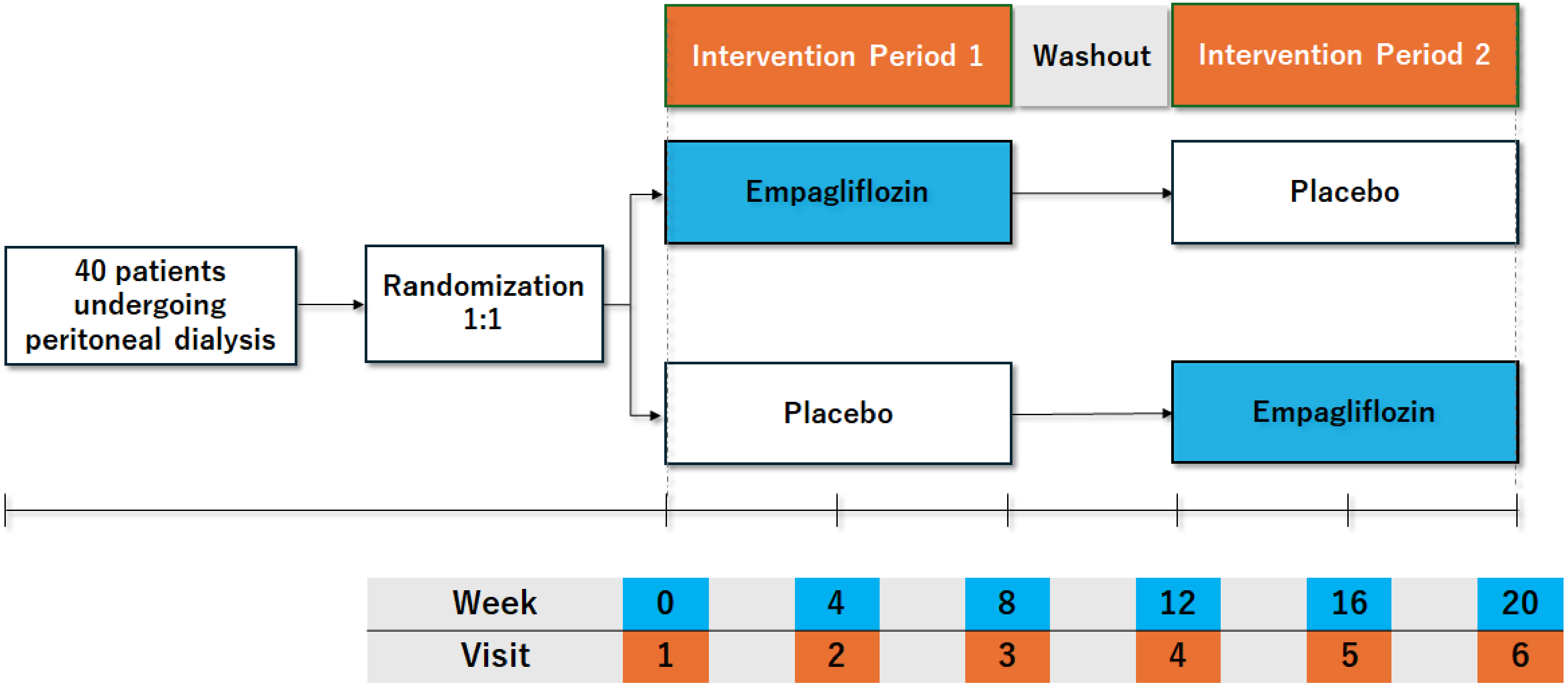

Supplementary Figure 2. Participant flow diagram

Of the 41 participants assessed for eligibility, 40 were randomized in a two-period crossover design. Twenty participants were allocated to empagliflozin in Period 1, of whom three discontinued due to adverse events. The remaining 16 crossed over to placebo in Period 2 and completed the study. In the placebo-first group (n=20), one participant discontinued during the washout period due to an adverse event, and 17 crossed over to empagliflozin in Period 2. Of these, two discontinued due to adverse events. A total of 16 participants in the empagliflozin-first sequence and 15 in the placebo-first sequence completed the study.

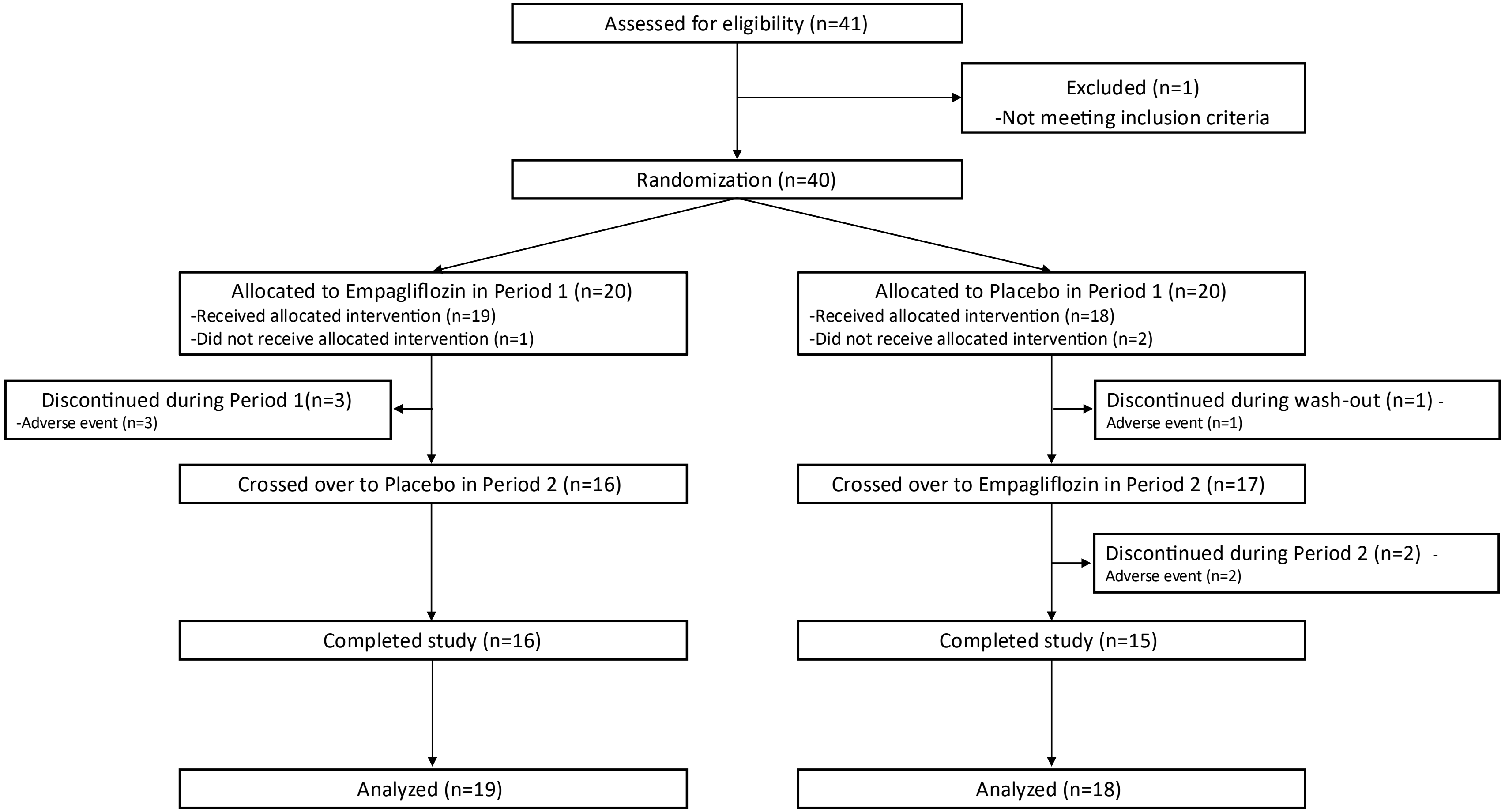
Assessed for eligibility (n=41)
Excluded (n=1)
-Not meeting inclusion criteria
Randomization (n=40)
Allocated to Empagliflozin in Period 1 (n=20)
-Received allocated intervention (n=19)
-Did not receive allocated intervention (n=1)
Allocated to Placebo in Period 1 (n=20)
-Received allocated intervention (n=18)
-Did not receive allocated intervention (n=2)
Discontinued during Period 1(n=3)
-Adverse event (n=3)
Discontinued during wash-out (n=1) -
Adverse event (n=1)
Crossed over to Placebo in Period 2 (n=16)
Crossed over to Empagliflozin in Period 2 (n=17)
Discontinued during Period 2 (n=2) -
Adverse event (n=2)
Completed study (n=16)
Completed study (n=15)
Analyzed (n=19)
Analyzed (n=18)

Supplementary Figure 3. Baseline daily ultrafiltration volume by treatment sequence.

Overlaid histograms showing the distribution of baseline daily ultrafiltration volume, stratified by treatment sequence (placebo → empagliflozin vs. empagliflozin → placebo).

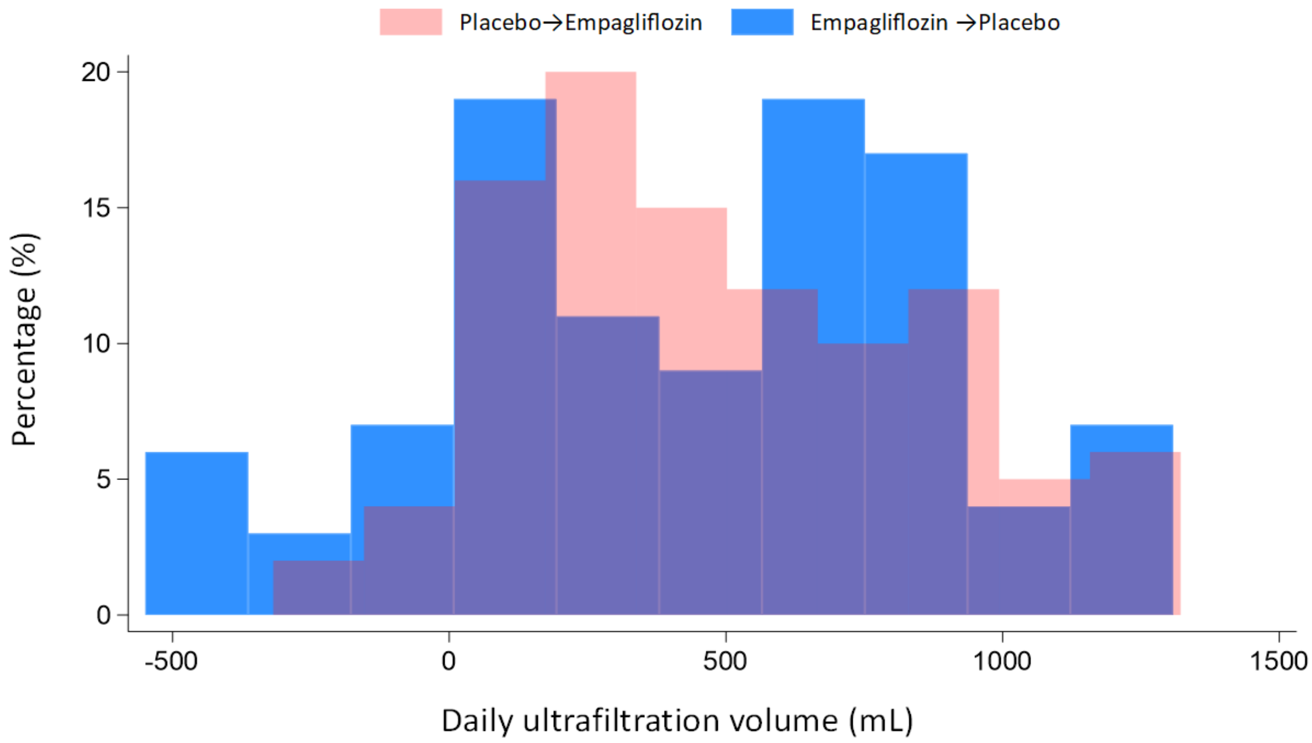

Supplementary Figure 4. Changes in daily glucose-based peritoneal dialysis solution volume over time

Values are presented as medians with interquartile ranges at each study visit, stratified by treatment sequence (placebo → empagliflozin vs. empagliflozin → placebo).

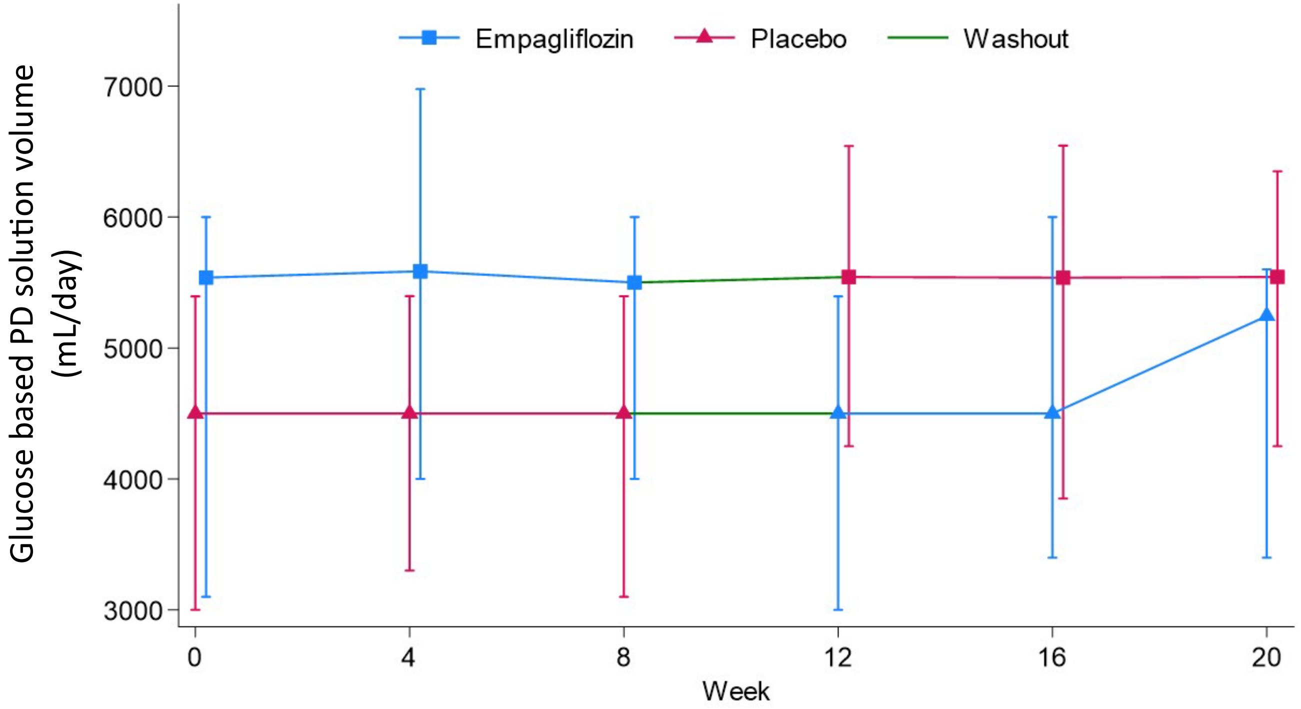

Supplementary Figure 5. Changes in 24-hour urine parameters over time.

Values are presented as medians with interquartile ranges at each study visit, stratified by treatment sequence (placebo → empagliflozin vs. empagliflozin → placebo). (A) Urine volume (B) Urine glucose excretion (C) Urine sodium excretion (D) Urine protein excretion.

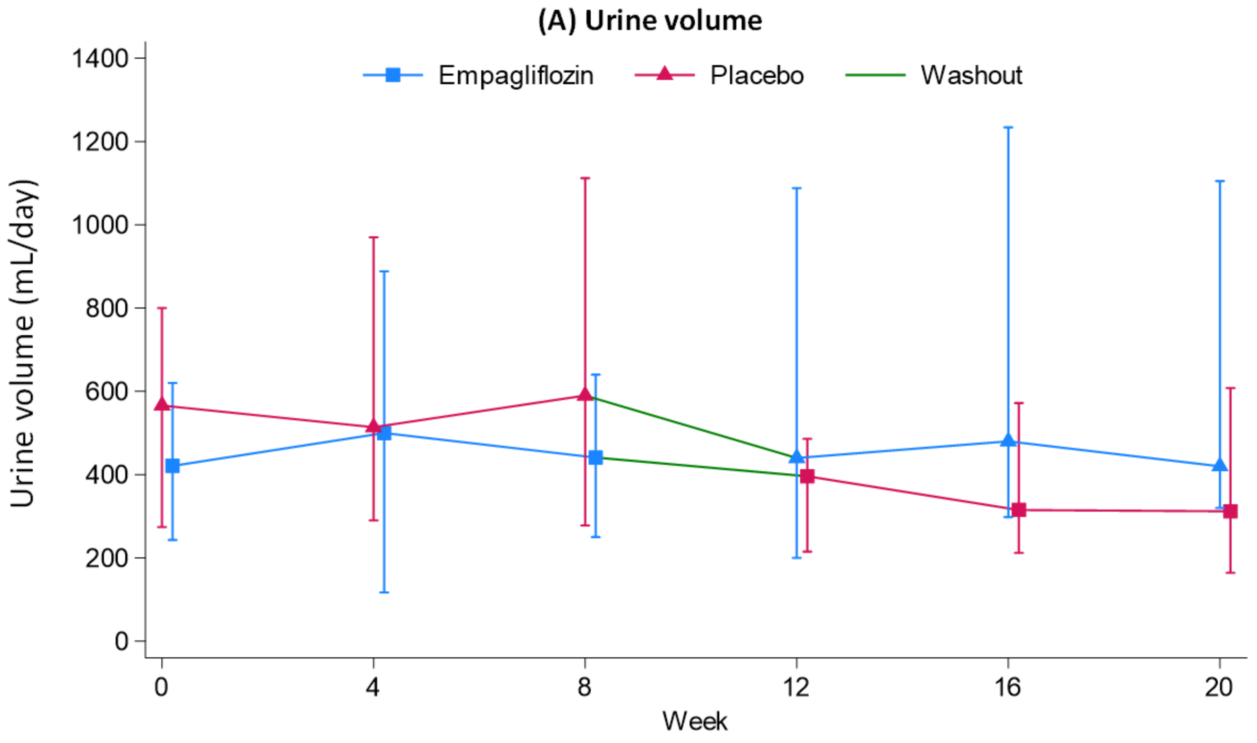

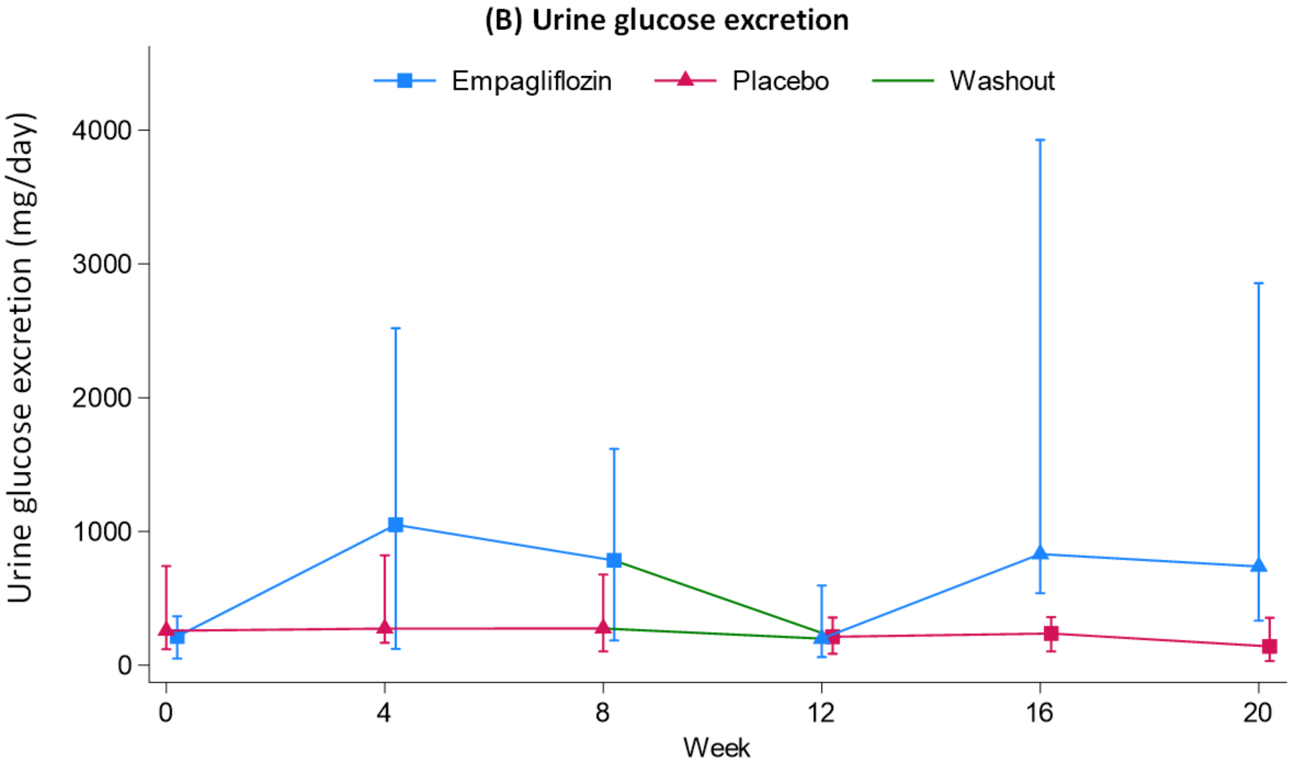
(B) Urine glucose excretion
Empagliflozin
Placebo
Washout
Urine glucose excretion (mg/day)
4000
3000
2000
1000
0
0
4
8
12
16
20
Week

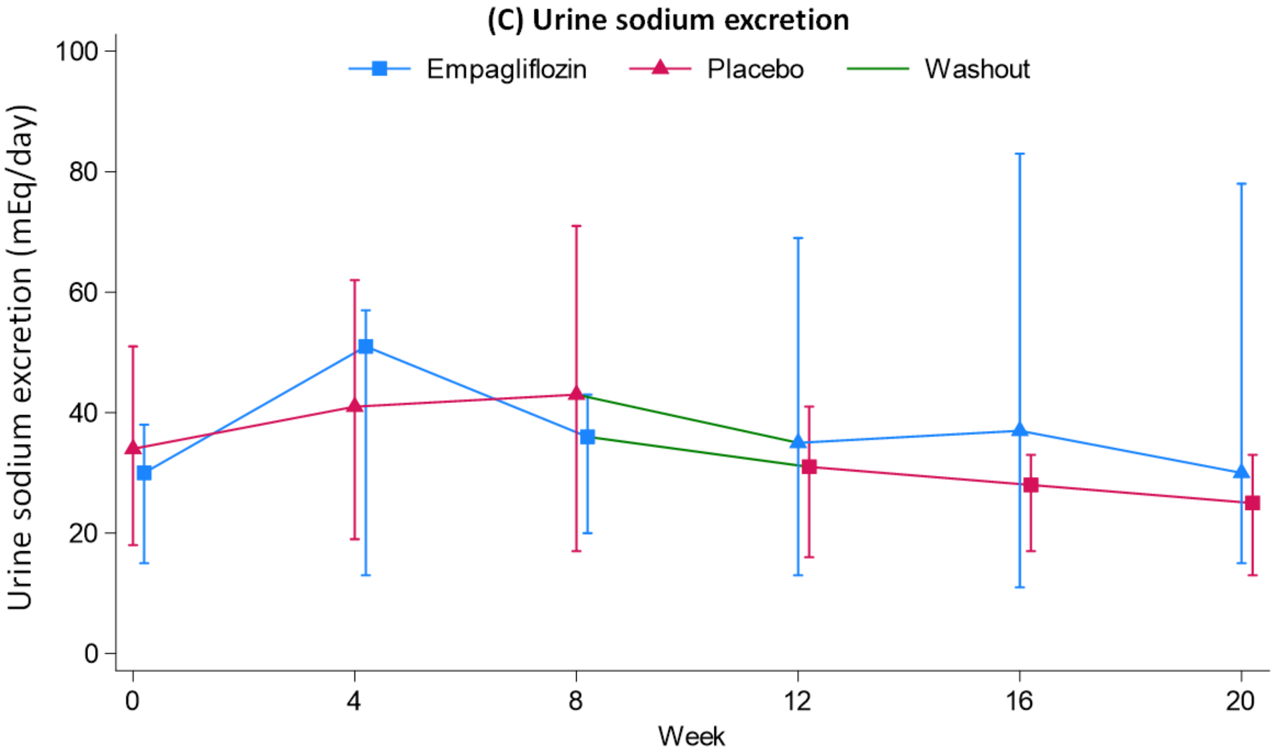

(C) Urine sodium excretion
Empagliflozin
Placebo
Washout
Urine sodium excretion (mEq/day)
100
80
60
40
20
0
0
4
8
12
16
20
Week

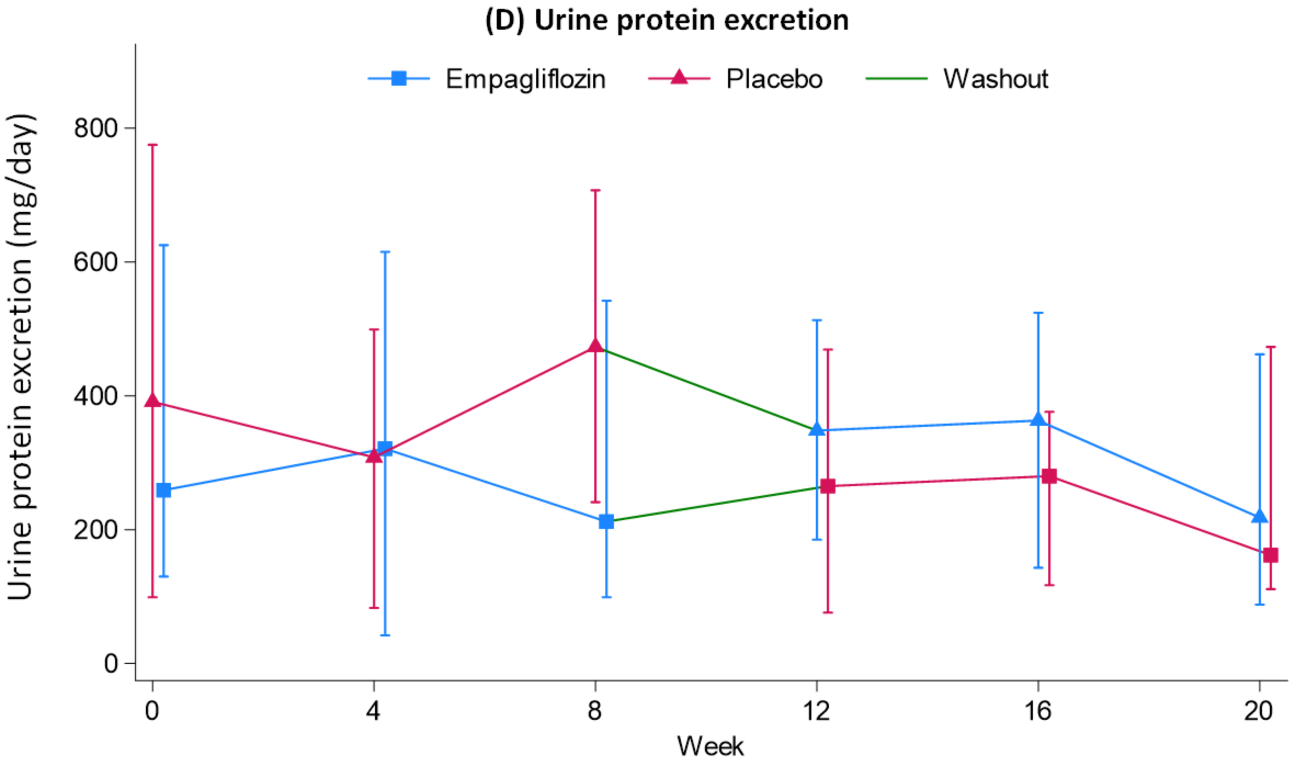

(D) Urine protein excretion
Empagliflozin
Placebo
Washout
Urine protein excretion (mg/day)
800
600
400
200
0
0
4
8
12
16
20
Week

Supplement 1. Protocols for Biomarker Measurement and Peritoneal Equilibration Testing

Biomarker Measurements

Chemical parameters were measured using standard automated techniques. Serum NT-proBNP levels were quantified using an electrochemiluminescence immunoassay (ECLIA) with the Elecsys proBNP II assay (Roche Diagnostics, Switzerland). CA125 levels in peritoneal dialysate effluent were measured by chemiluminescent enzyme immunoassay (CLEIA) using the Lumipulse Presto CA125 II reagent (Fujirebio Inc, Japan). IL-6 concentrations in dialysate were determined via CLEIA with dedicated IL-6 cartridges (Fujirebio Inc, Japan). Urinary KIM-1 levels were measured using a quantitative sandwich enzyme-linked immunosorbent assay (ELISA) with the Quantikine Human Urinary KIM-1 Immunoassay

Procedure for the Fast Peritoneal Equilibration Test (PET)*

(R&D Systems, USA).

1. Close all windows and doors, turn off fans, and avoid cleaning activities to minimize airborne contaminants.
2. Connect the patient to 2 L of warmed peritoneal dialysate (or an equivalent osmotic solution) with a glucose concentration of 2.27%.
3. Drain the peritoneal cavity completely for at least 20 minutes.
4. Perform a 15-second flush before filling.
5. Infuse the full 2 L of dialysate into the peritoneal cavity.
6. Record the exact completion time of infusion and begin a 4-hour dwell.
7. After 4 hours, connect the patient to a new dialysis bag.
8. Drain the effluent for at least 20 minutes to fully empty the peritoneal cavity.
9. Collect a blood sample at the 4-hour mark.
10. Perform another 15-second flush and continue with peritoneal dialysis exchange per local standard operating procedures.
11. Collect an effluent sample from the drainage bag as follows:
    a. Gently mix the effluent by shaking the bag.

b. Disinfect the medication port with a 2% chlorhexidine swab and allow it to air dry.

c. Using an aseptic non-touch technique, withdraw 10 mL of effluent with a sterile 10 mL syringe and needle.

12. Document the procedure in the nursing notes, including infused and drained volumes.

*Adapted from Morelle J, Stachowska-Pietka J, Öberg C, et al. ISPD recommendations for the evaluation of peritoneal membrane dysfunction in adults: Classification, measurement, interpretation and rationale for intervention. Perit Dial Int. 2021 Jul;41(4):352-372.

Supplementary References

S1. Devolder I, Verleysen A, Vijt D, Vanholder R, Van Biesen W. Body composition, hydration, and related parameters in hemodialysis versus peritoneal dialysis patients. Perit Dial Int. 2010 Mar-Apr;30(2):208-14. doi: 10.3747/pdi.2008.00284. Epub 2010 Jan 15. PMID: 20081049.

S2. Ling CHY, de Craen AJM, Slagboom PE, Gunn DA, Stokkel MPM, Westendorp RGJ, Maier AB. Accuracy of direct segmental multi-frequency bioimpedance analysis in the assessment of total body and segmental body composition in middle-aged adult population. Clin Nutr. 2011 Oct;30(5):610–5.

Section/topic	No	CONSORT 2025 checklist item description	Reported on page no.
**Title and abstract**			
Title and structured abstract	1a	Identification as a randomised trial	1
	1b	Structured summary of the trial design, methods, results, and conclusions	4-5
**Open science**			
Trial registration	2	Name of trial registry, identifying number (with URL) and date of registration	7, 21
Protocol and statistical analysis plan	3	Where the trial protocol and statistical analysis plan can be accessed	The full study protocol and statistical analysis plan are available in the Supplementary Appendix.
Data sharing	4	Where and how the individual de-identified participant data (including data dictionary), statistical code and any other materials can be accessed	24
Funding and conflicts of interest	5a	Sources of funding and other support (eg, supply of drugs), and role of funders in the design, conduct, analysis and reporting of the trial	22
	5b	Financial and other conflicts of interest of the manuscript authors	21-22
**Introduction**			
Background and rationale	6	Scientific background and rationale	6
Objectives	7	Specific objectives related to benefits and harms	6-7
**Methods**			
Patient and public involvement	8	Details of patient or public involvement in the design, conduct and reporting of the trial	Patients and the public were not involved in the design, conduct, or reporting of this study.
Trial design	9	Description of trial design including type of trial (eg, parallel group, crossover), allocation ratio, and framework (eg, superiority, equivalence, non-inferiority, exploratory)	7-8
Changes to trial protocol	10	Important changes to the trial after it commenced including any outcomes or analyses that were not prespecified, with reason	Not applicable
Trial setting	11	Settings (eg, community, hospital) and locations (eg, countries, sites) where the trial was conducted	7
Eligibility criteria	12a	Eligibility criteria for participants	7-8
	12b	If applicable, eligibility criteria for sites and for individuals delivering the interventions (eg, surgeons, physiotherapists)	Not applicable

Intervention and comparator	13	Intervention and comparator with sufficient details to allow replication. If relevant, where additional materials describing the intervention and comparator (eg, intervention manual) can be accessed	8
Outcomes	14	Prespecified primary and secondary outcomes, including the specific measurement variable (eg, systolic blood pressure), analysis metric (eg, change from baseline, final value, time to event), method of aggregation (eg, median, proportion), and time point for each outcome	9
Harms	15	How harms were defined and assessed (eg, systematically, non-systematically)	Harms were defined as any adverse events, collected systematically at each study visit via patient interviews and clinical assessments, and coded per MedDRA v27.1; severity and relatedness adjudicated per protocol (see Methods, and Table 3 footnote).
Sample size	16a	How sample size was determined, including all assumptions supporting the sample size calculation	9-10
	16b	Explanation of any interim analyses and stopping guidelines	Not applicable
Randomisation:			
Sequence generation	17a	Who generated the random allocation sequence and the method used	8
	17b	Type of randomisation and details of any restriction (eg, stratification, blocking and block size)	8
			**Reported on page no.**
Allocation concealment mechanism	18	Mechanism used to implement the random allocation sequence (eg, central computer/telephone; sequentially numbered, opaque, sealed containers), describing any steps to conceal the sequence until interventions were assigned	8
Implementation	19	Whether the personnel who enrolled and those who assigned participants to the interventions had access to the random allocation sequence	8
Blinding	20a	Who was blinded after assignment to interventions (eg, participants, care providers, outcome assessors, data analysts)	8
	20b	If blinded, how blinding was achieved and description of the similarity of interventions	8
Statistical methods	21a	Statistical methods used to compare groups for primary and secondary outcomes, including harms	10-12
	21b	Definition of who is included in each analysis (eg, all randomised participants), and in which group	10-11

	21c	How missing data were handled in the analysis	12
	21d	Methods for any additional analyses (eg, subgroup and sensitivity analyses), distinguishing prespecified from post hoc	11
**Results**			
Participant flow, including flow diagram	22a	For each group, the numbers of participants who were randomly assigned, received intended intervention, and were analysed for the primary outcome	12-13
	22b	For each group, losses and exclusions after randomisation, together with reasons	12-13, Supplementary Figure 2
Recruitment	23a	Dates defining the periods of recruitment and follow-up for outcomes of benefits and harms	7
	23b	If relevant, why the trial ended or was stopped	Not applicable
Intervention and comparator delivery	24a	Intervention and comparator as they were actually administered (eg, where appropriate, who delivered the intervention/comparator, how participants adhered, whether they were delivered as intended (fidelity))	8-9
	24b	Concomitant care received during the trial for each group	14, Supplementary Table 2
Baseline data	25	A table showing baseline demographic and clinical characteristics for each group	Table 1
Numbers analysed, outcomes and estimation	26	For each primary and secondary outcome, by group:<br>● the number of participants included in the analysis<br>● the number of participants with available data at the outcome time point<br>● result for each group, and the estimated effect size and its precision (such as 95% confidence interval)<br>● for binary outcomes, presentation of both absolute and relative effect size	13-15
Harms	27	All harms or unintended events in each group	15-16, Table 3, Supplementary Table 5, Supplementary Table 6
Ancillary analyses	28	Any other analyses performed, including subgroup and sensitivity analyses, distinguishing pre-specified from post hoc	13-15
**Discussion**			
Interpretation	29	Interpretation consistent with results, balancing benefits and harms, and considering other relevant evidence	16-19
Limitations	30	Trial limitations, addressing sources of potential bias, imprecision, generalisability, and, if relevant, multiplicity of analyses	19

Citation: Hopewell S, Chan AW, Collins GS, Hróbjartsson A, Moher D, Schulz KF, et al. CONSORT 2025 Statement: updated guideline for reporting randomised trials. BMJ. 2025; 388:e081123. https://dx.doi.org/10.1136/bmj-2024-081123
© 2025 Hopewell et al. This is an Open Access article distributed under the terms of the Creative Commons Attribution License (https://creativecommons.org/licenses/by/4.0/), which permits unrestricted use, distribution, and reproduction in any medium, provided the original work is properly cited.

*We strongly recommend reading this statement in conjunction with the CONSORT 2025 Explanation and Elaboration and/or the CONSORT 2025 Expanded Checklist for important clarifications on all the items. We also recommend reading relevant CONSORT extensions. See www.consort-spirit.org.

Specified Clinical Research Protocol

The effects of empagliflozin on ultrafiltration in patients with peritoneal dialysis: a randomized, double-blind, crossover trial (EMPOWERED trial)

Principal investigator Yohei Doi
Department of Nephrology, Osaka University Hospital
TEL: +81-6-6879-5111 FAX: +81-6-6879-5019

Protocol No.	OUN-0002
Date of preparation	July 24, 2024
Version	3.2

Protocol No.: OUN-0002

Version management

Version No.	Date of preparation and date of revision
1.0	April 11, 2023
1.1	May 23, 2023
2.0	July 27, 2023
3.0	December 14, 2023
3.1	April 18, 2024
3.2	July 24, 2024

Synopsis

1. Objective and Description of the Clinical Study

Objective of the study	The study aims to explore the effect of empagliflozin on the amount of water removed and its safety in patients with heart failure on peritoneal dialysis using a placebo as a comparator.
Expected duration of the study	The jRCT publication date to December 31, 2025
Planned number of study participants	36
Study design	A multicenter, randomized, double-blind, crossover study
Name of the target disease	Chronic heart failure treated with peritoneal dialysis
Inclusion criteria	(1) Individuals aged between 18 and 90 years at the time of providing informed consent<br>(2) Individuals with BNP ≥40 pg/mL, NT-proBNP ≥400 pg/mL, structural heart disease (left atrial enlargement and/or left ventricular hypertrophy), increased ventricular filling pressure, or a history of hospitalization due to heart failure. Reference values for NT-proBNP should be used for patients using ARNIs instead of those for BNP.<br>(3) Individuals who have received standard drug therapy for heart failure (at least one of the following: loop diuretics, ACEIs, ARBs, ARNIs, beta-blockers, or mineralocorticoid receptor antagonists [MRAs])<br>(4) Individuals who have undergone peritoneal dialysis for at least 3 months<br>(5) Individuals who use at least 3 L of glucose peritoneal dialysate solutions per day<br>(6) Individuals who can provide a written informed consent to participate in the study
Exclusion criteria	(1) Individuals who are using or have used SGLT2 inhibitors within the past 3 months<br>(2) Individuals who are not expected to live for 1 year or more after enrollment<br>(3) Individuals who receive hybrid therapy comprising peritoneal dialysis and hemodialysis<br>(4) Individuals who have or have had peritonitis within the past 2 months<br>(5) Women who are pregnant or nursing<br>(6) Individuals who have an uncontrollable infection

	(7) Individuals who participate in clinical studies (trials and research) involving other interventions<br>(8) Individuals disqualified from participation in the study by the investigator or subinvestigator for any other reasons
Protocol treatment	1. Preemptive empagliflozin group<br>Empagliflozin is orally administered at a dose of 10 mg once daily before or after breakfast for 8 weeks and after a washout period of 4 weeks; the placebo is orally administered once daily before or after breakfast for 8 weeks.<br><br>2. Preemptive placebo group<br>The placebo is orally administered once daily before or after breakfast for 8 weeks and after a washout period of 4 weeks; empagliflozin is orally administered at a dose of 10 mg once daily before or after breakfast for 8 weeks.
Discontinuation criteria	(1) When an adverse event occurred and the investigator or subinvestigator considers that continued participation in the study would impose an unacceptable risk on the study participant<br>(2) When the study participant requested study discontinuation<br>(3) When the prohibited concomitant drugs were used<br>(4) When the study participant is found to be ineligible for the study<br>(5) When the study participant completely stopped peritoneal dialysis due to full transition to hemodialysis or renal transplantation<br>(6) When the study participant developed peritonitis<br>(7) When it turned out that the study participant was unable to complete the necessary observations and tests for his/her personal reasons, including change of residence<br>(8) When the investigator or subinvestigator considers that the intervention should be discontinued
Primary endpoint	Change in the amount of water removed per day to the glucose peritoneal dialysate solutions at Week 8 (The amount of water removed per day is calculated as the mean of all results for 5 of 7 consecutive days, except for the day when the maximum and minimum values were recorded)
Secondary endpoints	(1) Changes in NT-proBNP and BNP

	(2) Changes in FAST PET-related factors: Amount of water removed, sodium, potassium, glucose, urea nitrogen, creatinine, uric acid, protein, IL-6, and CA-125 and drainage-to-serum creatinine ratio<br>(3) Changes in factors related to 24-hour urine collection: Urine volume, sodium, potassium, glucose, urea nitrogen, creatinine, uric acid, urine protein, and urea and creatinine clearance (the mean of urea and creatinine clearance)<br>(4) Urine KIM-1<br>(5) Changes in anemia-related factors: Hemoglobin, hematocrit, ferritin, and transferrin saturation<br>(6) Changes in body weight and blood pressure<br>(7) Changes in body composition: Intracellular and extracellular fluid volume, fluid overload<br>(8) Adverse events

2. Study Schematic (Schema)

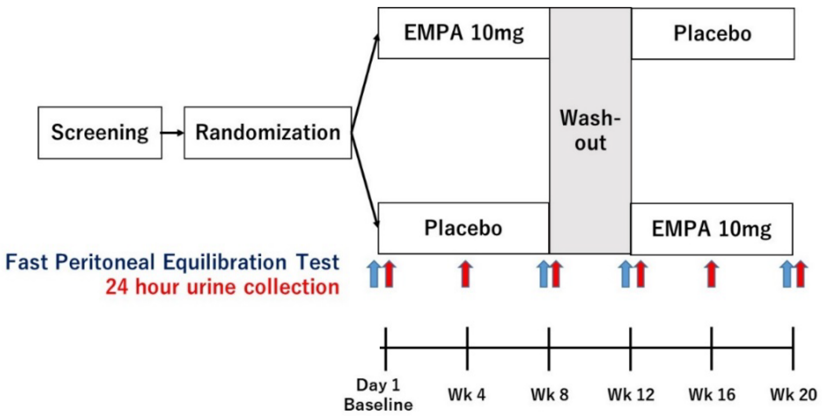

3. Observation, Test, and Evaluation Schedule

		Period 1			Washout 4 weeks	Period 2			
	At enrollment	At the start of the treatment	Week 4	Week 8		Week 12	Week 16	Week 20	At discon-tinuation
Visit	0	1	2	3		4	5	6	
Day		1 (day 1)	29	57		85 (day 1)	113	141	
Acceptable range (days)		-	±7	±14		Date of study visit at Week 8 + 28 (−7, +14)	Date of study visit at Week 12 + 28 (±7)	Date of study visit at Week 12 + 56 (±14)	
Informed consent	●								
Enrollment and assignment	●								
Study participants' demographics		●							
Comorbidities		●							
Concomitant drugs		←	—	—	—	—	—	—	→
Study drug administration		←	—	→		←	—	→	
Amount of water removed per day		←	—	→		←	—	→	●
NT-proBNP	○*	●		●		●		●	○
BNP	○*	●	●	●		●	●	●	○
Echocardiography	○**								
FAST PET		●		●		●		●	
24-hour urine collection***		●	●	●		●	●	●	
Hematology and blood biochemistry		●	●	●		●	●	●	○
Body weight and blood pressure		●	●	●		●	●	●	○
Body composition		●		●		●		●	
Adverse events		←	—	—	—	—	—	—	→

●, required; ○, if necessary

*; Data collected from routine medical practice 182 days before providing informed consent may be used for assessment.

**: Data collected from routine medical practice 365 days before providing informed consent may be used for assessment.

***: Urine KIM-1 is measured only on Visit 3 and Visit 6.

Table of Contents

1. DEFINITION OF ABBREVIATIONS AND TERMS 1
1.1. ABBREVIATIONS 1
1.2. DEFINITION OF TERMS 1
2. STUDY BACKGROUND 3
2.1. NAME OF THE TARGET DISEASE 3
2.2. CONCEPT OF THE TARGET DISEASE 3
2.3. EPIDEMIOLOGY OF THE TARGET DISEASE 4
2.4. STANDARD OF CARE 5
2.5. DRUGS TO BE EVALUATED IN THE STUDY 6
2.6. SIGNIFICANCE OF CONDUCTING THE STUDY 6
3. OBJECTIVE OF THE STUDY AND ENDPOINTS 8
3.1. OBJECTIVE OF THE STUDY 8
3.2. PRIMARY ENDPOINT 8
3.3. SECONDARY ENDPOINTS 8
4. STUDY DESIGN 10
4.1. STUDY DESIGN 10
4.2. PLANNED NUMBER OF STUDY PARTICIPANTS IN THE STUDY 10
4.3. EXPECTED DURATION OF THE STUDY 11
5. SELECTION OF STUDY PARTICIPANTS 12
5.1. INCLUSION CRITERIA 12
5.2. EXCLUSION CRITERIA 13
6. DRUGS TO BE STUDIED 14
6.1. SUMMARY OF THE DRUGS TO BE STUDIED 14
6.1.1. INVESTIGATIONAL PRODUCT 14
6.1.1. CONTROL DRUG 14
6.2. ASSURANCE OF QUALITY OF THE DRUGS TO BE STUDIED 15
7. METHOD OF APPLICATION OF DRUGS TO STUDY PARTICIPANTS (PROTOCOL TREATMENT) 16

7.1. METHOD OF APPLICATION OF DRUGS TO SUBJECTS 16
7.1.1. PREEMPTIVE EMPAGLIFLOZIN GROUP 16
7.1.2. PREEMPTIVE PLACEBO GROUP 16
7.1.3. RATIONALE FOR THE METHOD OF APPLICATION OF DRUGS TO SUBJECTS 16
7.2. CONCOMITANT DRUGS AND THERAPIES 16
7.3. PROHIBITED CONCOMITANT DRUGS 16
8. OBSERVATION, TEST, AND EVALUATION AT EACH TIME POINT 18
8.1. OBSERVATION, TEST, AND EVALUATION SCHEDULE 18
8.1.1. AT ENROLLMENT 18
8.1.2. AT THE START OF THE TREATMENT (VISIT 1) 18
8.1.3. WEEK 4 (VISIT 2) 18
8.1.4. WEEK 8 (VISIT 3) 19
8.1.5. WEEK 12 (VISIT 4) 19
8.1.6. WEEK 16 (VISIT 5) 19
8.1.7. WEEK 20 (VISIT 6) 20
8.1.8. AT DISCONTINUATION 20
8.2. STUDY CALENDAR 21
9. OBSERVATION, TEST, AND EVALUATION PROCEDURES 22
9.1. INFORMED CONSENT 22
9.2. ENROLLMENT AND ASSIGNMENT 22
9.2.1. ENROLLMENT AND ASSIGNMENT PROCEDURES 22
9.2.2. CONTACT INFORMATION ABOUT ENROLLMENT PROCEDURES 22
9.2.3. CREATION AND RETENTION OF A RANDOMIZATION SCHEDULE 22
9.2.4. EMERGENCY KEY CODE BREAKING 23
9.3. STUDY TREATMENT STATUS 23
9.4. STUDY PARTICIPANTS' DEMOGRAPHICS 23
9.5. COMORBIDITIES 23
9.6. CONCOMITANT DRUGS 23
9.7. AMOUNT OF WATER REMOVED PER DAY 24

9.8. BNP AND NT-PROBNP 24
9.9. FREQUENTLY AND SHORT-TIME PERITONEAL EQUILIBRATION TEST (FAST PET) 24
9.10. 24-HOUR URINE COLLECTION 25
9.11. HEMATOLOGY AND BLOOD BIOCHEMISTRY 25
9.12. BODY WEIGHT AND BLOOD PRESSURE 25
9.13. BODY COMPOSITION 25
9.14. CRITERIA FOR DISCONTINUATION AND TERMINATION IN EACH STUDY PARTICIPANT 25
9.14.1. DISCONTINUATION FOR EACH STUDY PARTICIPANT 25
9.14.2. DISCONTINUATION PROCEDURES FOR EACH STUDY PARTICIPANT 26
9.14.3. TERMINATION FOR EACH STUDY PARTICIPANT 26
10. ADVERSE EVENTS 27
10.1. DEFINITION OF ADVERSE EVENTS 27
10.2. SURVEY PERIOD FOR ADVERSE EVENTS 27
10.3. ASSESSMENT OF ADVERSE EVENTS 27
10.3.1. INVESTIGATION OF SUBJECTIVE SYMPTOMS AND OBJECTIVE FINDINGS 27
10.3.2. ABNORMAL CHANGES IN LABORATORY VALUES 27
10.4. EVALUATION OF ADVERSE EVENTS 27
10.4.1. NAME OF THE ADVERSE EVENT 28
10.4.2. DATE OF ONSET 28
10.4.3. SEVERITY 29
10.4.4. SERIOUSNESS 29
10.4.5. CAUSAL RELATIONSHIP WITH THE STUDY 30
10.4.6. CAUSAL RELATIONSHIP WITH THE STUDY DRUG 30
10.4.7. DATE OF OUTCOME 30
10.4.8. OUTCOME 30
10.5. MEASURES TAKEN FOR THE ADVERSE EVENTS 31
10.5.1. TREATMENT PROVIDED TO STUDY PARTICIPANTS 31
10.5.2. FOLLOW-UP STUDY OF ADVERSE EVENTS 31
10.5.3. REPORTING TO THE FINANCIAL CONTRIBUTOR 31

10.6. ADVERSE EVENTS EXPECTED TO OCCUR DURING THE STUDY 32
10.6.1. INFORMATION PROVIDED ON THE PACKAGE INSERT OF EMPAGLIFLOZIN 32
10.6.2. ADVERSE EVENTS IN PATIENTS WITH RENAL IMPAIRMENT 33
10.7. ADVERSE EVENT OF SPECIAL INTEREST (AESI) 34
11. DISEASES 38
11.1. DEFINITION OF DISEASES 38
11.2. PROCEDURES FOR REPORTING SERIOUS DISEASES TO THE CERTIFIED REVIEW BOARD 38
11.3. PROCEDURES FOR REPORTING SERIOUS DISEASES TO THE MINISTER OF HEALTH, LABOUR AND WELFARE 39
12. DATA MANAGEMENT 40
12.1. DATA MANAGEMENT PLAN 40
12.2. CENTRAL MONITORING 40
12.3. CASE REPORT FORMS 40
12.4. DATA DIRECTLY RECORDED ON THE CASE REPORT FORMS 40
13. STATISTICAL CONSIDERATION 41
13.1. DEFINITION OF AN ANALYSIS SET 41
13.2. DATA HANDLING 41
13.2.1. HANDLING OF MEASURED VALUES OUT OF THE PREDETERMINED ACCEPTABLE RANGE 41
13.2.2. HANDLING OF OUTLIERS 41
13.2.3. HANDLING OF MISSING VALUES 41
13.3. ANALYTICAL METHODS 42
13.3.1. STUDY PARTICIPANTS' DEMOGRAPHICS 42
13.3.2. STUDY TREATMENT COMPLIANCE 42
13.3.3. CONCOMITANT DRUGS AND PERITONEAL DIALYSIS 42
13.3.4. PRIMARY ENDPOINT 42
13.3.5. SECONDARY ENDPOINTS 43
13.3.6. ADVERSE EVENTS 44
13.4. INTERIM ANALYSIS AND CRITERIA FOR PREMATURE DISCONTINUATION 44
13.5. CHANGES TO THE STATISTICAL ANALYSIS PLAN 44

14. QUALITY CONTROL AND QUALITY ASSURANCE 45
14.1. QUALITY CONTROL POLICIES 45
14.2. QUALITY OBJECTIVES 45
14.3. MONITORING 45
14.4. AUDIT 45
14.5. RESPONSE TO INSPECTION BY THE REGULATORY AUTHORITIES, ETC. 46
14.6. NONCONFORMITY 46
14.6.1. DEFINITION OF NONCONFORMITY 46
14.6.2. CRITICAL NONCONFORMITY 46
14.6.3. NONCONFORMITY MANAGEMENT PROCEDURES 46
15. ETHICAL CONSIDERATION 47
15.1. VARIOUS RULES TO BE FOLLOWED 47
15.2. APPROVAL OF THE CERTIFIED REVIEW BOARD AND DIRECTOR OF THE STUDY SITE 47
15.3. STUDY COST BURDEN ON THE STUDY PARTICIPANTS 47
15.4. INFORMED CONSENT FORMS AND CONSENT OF STUDY PARTICIPANTS 47
15.4.1. PROCEDURES FOR OBTAINING INFORMED CONSENT 47
15.4.2. INFORMATION THAT SHOULD BE INCLUDED IN THE INFORMED CONSENT FORM 48
15.5. CONTACT INFORMATION FOR STUDY PARTICIPANTS 49
15.6. ANTICIPATED ADVANTAGES AND DISADVANTAGES FOR STUDY PARTICIPANTS 49
15.6.1. ANTICIPATED ADVANTAGES 49
15.6.2. ANTICIPATED DISADVANTAGES 49
15.6.3. OVERALL ASSESSMENT OF ADVANTAGES AND DISADVANTAGES AND MEASURES TO MINIMIZE THE DISADVANTAGES 50
15.7. CONFIDENTIALITY OF STUDY PARTICIPANTS (PROTECTION OF PERSONAL INFORMATION) 50
16. COMPENSATION FOR STUDY-RELATED INJURY 51
17. DISCONTINUATION OR TERMINATION OF THE ENTIRE STUDY 52
17.1. DISCONTINUATION CRITERIA 52
17.2. DISCONTINUATION PROCEDURE 52
17.3. TERMINATION CRITERIA 52

18. STUDY INFORMATION DISCLOSURE AND PUBLICATION OF RESULTS 53
18.1. STUDY REGISTRATION 53
18.2. PUBLICATION OF STUDY RESULTS 53
18.2.1. PRIMARY ENDPOINT REPORTS 53
18.2.2. CLINICAL STUDY REPORT 53
18.2.3. PRESENTATION AT SCIENTIFIC MEETINGS, ETC. 54
19. CHANGE CONTROL 55
19.1. CHANGES IN DOCUMENTS APPROVED BY THE CERTIFIED REVIEW BOARD 55
19.2. CHANGES IN THE STUDY PLAN 55
19.3. MINOR CHANGES IN THE STUDY PLAN 55
20. CONFLICT OF INTEREST 56
20.1. SOURCE OF MONEY FOR THE STUDY 56
20.2. CONFLICT OF INTEREST MANAGEMENT 56
21. PERIODIC REPORTS SUBMITTED TO THE CERTIFIED REVIEW BOARD AND MINISTER OF HEALTH, LABOUR AND WELFARE 57
21.1. PERIODIC REPORTS SUBMITTED TO THE CERTIFIED REVIEW BOARD 57
21.1.1. ITEMS TO BE INCLUDED IN PERIODIC REPORTS 57
21.1.2. TIMING OF PERIODIC REPORTS 57
21.2. PERIODIC REPORTS SUBMITTED TO THE MINISTER OF HEALTH, LABOUR AND WELFARE 57
21.2.1. ITEMS TO BE INCLUDED IN PERIODIC REPORTS 57
21.2.2. TIMING OF PERIODIC REPORTS 57
22. METHOD OF STORING AND DISPOSING OF DOCUMENTS, RECORDS, ETC. 58
22.1. STORAGE OF SOURCE DOCUMENTS 58
22.2. STORAGE OF RECORDS AND DOCUMENTS REQUIRED BY LAW 58
22.3. STORAGE OF SAMPLES 59
22.4. SECONDARY USE OF SAMPLES AND INFORMATION 59
22.5. DISPOSAL PROCEDURES AND METHOD 59
23. OWNERSHIP OF THE STUDY RESULTS 60
24. STUDY ADMINISTRATIVE STRUCTURE 61

24.1. PRINCIPAL INVESTIGATOR 61

24.2. INVESTIGATORS 61

24.3. PERSON RESPONSIBLE FOR RANDOMIZATION 61

24.4. PERSON RESPONSIBLE FOR STATISTICAL ANALYSIS 61

24.5. PERSON WHO SUMMARIZES THE CLINICAL STUDY EXCEPT THE PRINCIPAL INVESTIGATOR AND INVESTIGATORS 61

24.6. PERSON RESPONSIBLE FOR DATA MANAGEMENT 62

24.7. PERSON RESPONSIBLE FOR MONITORING 62

24.8. PERSON RESPONSIBLE FOR AUDIT 62

24.9. COORDINATING ADMINISTRATOR 62

24.10. LABORATORY RELATED TO THE CLINICAL STUDY 62

24.11. CONTRACT RESEARCH ORGANIZATION 63

25. REFERENCES 64

1. Definition of Abbreviations and Terms

1.1. Abbreviations

Abbreviation	Complete term
ACEI	angiotensin converting enzyme inhibitor
ARB	angiotensin receptor blocker
ARNI	angiotensin receptor-neprilysin inhibitor
BNP	brain natriuretic peptide
eGFR	estimated glomerular filtration rate
FAST PET	frequently and short time peritoneal equilibration test
HD	hemodialysis
jRCT	Japan Registry of Clinical Trials
LVEF	left ventricular ejection fraction
MMRM	mixed-effects model repeated measures
NT-proBNP	N-terminal pro-brain natriuretic peptide
PD	peritoneal dialysis
SGLT	sodium glucose co-transporter

1.2. Definition of Terms

(1) Investigator

An investigator conducts a clinical study as specified in the Clinical Trials Act and oversees clinical study-related activities in the study site.

(2) Principal investigator

A principal investigator represents investigators in several study sites in a multicenter study.

(3) Subinvestigator

A subinvestigator is a physician who shares study-related activities under the guidance of the investigator in the study site.

(4) Monitoring

Monitoring is defined as an investigation conducted by the person designated by the investigator or principal investigator to examine the progress of the study and determine whether it is conducted in accordance with the Clinical Trials Act, Enforcement Regulations of the Clinical Trials Act, and protocol to ensure the reliability for the clinical study and the

study is conducted properly from the viewpoint of study participants protection.

(5) Audit

Audit is defined as an investigation conducted by the person designated by the investigator or principal investigator to determine whether the study is conducted in accordance with the Clinical Trials Act, Enforcement Regulations of the Clinical Trials Act, and protocol to ensure the reliability for the clinical study and the data collected during the clinical study from the viewpoint of study participants protection.

(6) Research collaborator

A research collaborator is a pharmacist, a nurse, and other healthcare professionals who cooperate with the investigator or subinvestigator in performing study-related activities under their guidance in the study site.

2. Study Background

2.1. Name of the Target Disease

Chronic heart failure treated with peritoneal dialysis

2.2. Concept of the Target Disease

Heart failure is defined as a clinical syndrome consisting of dyspnea, malaise, swelling or decreased exercise capacity due to the loss of compensation for cardiac pumping function due to structural and/or functional abnormalities of the heart. [1] The American Heart Association (AHA)/American College of Cardiology (ACC) classifies heart failure into four stages (A, B, C, and D) (Table 1), [2] and advanced stages of heart failure are associated with reduced survival. [3]
As shown in Table 2, there is another classification based on the evaluation of left ventricular ejection fraction (LVEF).

Table 1 Classification of Stages of Heart Failure by ACC/AHA [2]

Stage A: At risk of heart failure	At risk of heart failure but without symptoms, structural heart disease, or abnormalities of cardiac biomarkers
Stage B: Preheart failure	No symptoms or signs of heart failure and evidence of one of the following:<br>• Structural heart disease<br>• Evidence for increased filling pressures<br>• Patients with risk factors and increased levels of BNPs or persistently elevated cardiac troponin
Stage C: Symptomatic heart failure	Structural heart disease with current or previous symptoms of heart failure
Stage D: Advanced heart failure	Marked heart failure symptoms that interfere with daily life and with recurrent hospitalizations despite attempts to optimize treatment

Table 2 Classification of Heart Failure by LVEF on Examination [1]

Phenotype	LVEF	Definition
Heart failure with reduced LVEF (HFrEF)	<40%	Left ventricular systolic dysfunction. In many clinical studies, patients with low LVEF despite standard medical treatment for heart failure are enrolled as patients with HFrEF.
Heart failure with preserved LVEF (HFpEF)	≥50%	Left ventricular diastolic dysfunction. Other diseases that may cause similar symptoms should be ruled out. No effective treatment has been established.
Heart failure with midrange LVEF (HFmrEF)	40%–<50%	Borderline heart failure. Clinical features and prognosis have not yet been fully characterized. Treatment should be selected on an individual condition.

As with HD and renal transplantation, PD is one of the renal replacement therapies provided to compensate for the loss of renal function, and a PD catheter placed in the peritoneal cavity is commonly used to infuse the hypertonic glucose solution (peritoneal dialysate solution), and waste products are removed through patient's own peritoneum via ultrafiltration (water removal) driven by an osmotic pressure gradient or diffusion.

2.3. Epidemiology of the Target Disease

The number of patients with heart failure has been increasing explosively worldwide, affecting over 60 million people, [4] and we face an ongoing situation called a heart failure pandemic. In Japan, heart failure also affects approximately 1.2 million people, and the number of patients with heart failure is estimated to be 1.3 million in 2030. [5] As the population ages, the prevalence of heart failure will grow, and in a representative large-scale observational study in Japan (Chronic Heart Failure Analysis and Registry in the Tohoku District 2 [CHART-2]), the elderly aged 65 years or older and late-stage elderly aged 75 years or older account for 68% and 34% of the enrolled subjects, respectively. [6] According to the summary report of monthly Vital Statistics (annual total; rough figures) (2021), heart failure is cited as a common cause of death in Japan (89,933 person-years, accounting for 6.2% of all deaths), and it is the most leading cause of death among patients with cardiovascular disease. In Japan, cardiovascular disease is categorized as a disease which is the second most common cause of death. (Malignant neoplasm ranks top in this category.) [7]

The number of dialysis patients with loss of renal function increases over time in Japan, and the total number of dialysis patients has reached 0.34 million by the end of FY2020; approximately 10,000 patients accounting for 3% of all such patients receive PD treatment. [8]

Because of the gradual aging of the dialysis population, dialysis patients had a mean age of 69.4 years according to the tabulation in FY2020. [8] In Japan, the cause of death in dialysis patients is heart failure (22.4%), followed by infection (21.5%) and malignancy (9.0%), [8] and heart failure is a critical disease in dialysis patients. Fluid overload, a major sign of heart failure, occurs more frequently in PD patients than in HD patients, [9] and at least 50% of PD patients present with fluid overload, which is associated with worsened outcomes. [10] In Japan, poor fluid control has been reported in 55% of patients who stopped PD, [11] and there are many patients who must transfer from home PD treatment to HD require frequent hospital visits due to poor fluid control.

2.4. Standard of Care

Drug therapy plays a major role in treating many patients with heart failure, and there are several therapeutic drugs shown to improve outcomes, especially for HFrEF. Besides conventional ACEIs/ARBs, beta-blockers, and mineralocorticoid receptor antagonists (MRAs), the replacement of SGLT2 inhibitors or ACEI/ARB with ARNIs was newly added as a standard therapeutic drug for HFrEF in the Guidelines for Diagnosis and Treatment of Acute and Chronic Heart Failure 2021. [1] Although no drugs have been proven to improve outcomes in patients with HFmrE/HFpEF until recent years, studies have demonstrated that SGLT2 inhibitors are effective in improving outcomes in not only patients with HFrEF but also those with HFmrE/HFpEF. [12] [13] In Japan, physicians were also allowed to prescribe empagliflozin, an SGLT2 inhibitor, for treating HFmrE/HFpEF in April 2022.

Dialysis therapy is called renal replacement therapy that uses proper ultrafiltration (water removal) and removes waste products (substances) instead of nonfunctioning native kidneys. During PD, peritoneal dialysate solutions are usually infused, retained, and drained several times in a day, and fluid overload/heart failure often occurs because of poor ultrafiltration (water removal). Patients with severe renal impairment are excluded from a large-scale clinical study evaluating the drug efficacy of SGLT2 inhibitors and ACEI/ARB/ARNI, beta-blockers, and MRA, all of which are standard therapeutic drugs for HFrEF, and no standard of care is available for fluid overload/heart failure in PD patients. [14] Measures such as restriction of salt and fluid intake, initiation and dose increase of diuretics, use of concentrated glucose peritoneal dialysate solutions, and concomitant use of icodextrin-containing peritoneal dialysates, are taken on an empirical basis; however, there are some problems, including poor efficacy, advanced damage to the peritoneal tissue due to the use of concentrated glucose peritoneal dialysate solutions, and expensive icodextrin-containing peritoneal dialysates or its limitation in Japan (once daily). [15]

2.5. Drugs to be Evaluated in the Study

Empagliflozin, an SGLT2 inhibitor, was approved for treating type 2 diabetes in December 2014 and chronic heart failure in November 2021.
For treating chronic heart failure, empagliflozin works by inhibiting the SGLT2 found in the proximal tubules in the kidneys. Moreover, through SGLT2 inhibition, it reduces renal reabsorption of not only glucose but also sodium and increases sodium delivery to the distal tubules, thereby potentially leading to changes in physiological function, such as increased tubuloglomerular feedback, reduced cardiac preload and afterload, and reduced sympathetic nervous activity. Additionally, empagliflozin has a direct effect on endothelial function, acts on metabolism by helping the body use ketone bodies as an alternate energy source for the heart, and reduces oxidative stress and inflammation, which may contribute to its effects on chronic heart failure.
In a placebo-controlled, double-blind comparative study, patients with chronic heart failure with reduced LVEF orally received add-on empagliflozin at a dose of 10 mg once daily and other therapeutic drugs for chronic heart failure. Additionally, when the median duration of treatment and follow-up was approximately 1.2 and 1.3 years, respectively, the incidence of cardiovascular death or (initial) hospitalization for heart failure was 19.4% and 24.7% in the empagliflozin 10-mg (n = 1863) and placebo (n = 1867) groups, respectively, with a hazard ratio (95% confidence interval) of 0.75 (0.65, 0.86). Adverse drug reactions occurred in 15.2% and 12.2% of patients in the empagliflozin 10-mg and placebo groups, respectively, and the most common adverse drug reaction was hypotension (10 mg, 2.3%; placebo, 1.8%).
In a similar study of patients with chronic heart failure with preserved LVEF, the incidence of cardiovascular death or (initial) hospitalization for heart failure was 13.8% and 17.1% in the empagliflozin 10-mg (n = 2997) and placebo (n = 2991) groups, respectively, with a hazard ratio (95% confidence interval) of 0.79 (0.69, 0.90), when the median duration of treatment and follow-up was approximately 1.9 and 1.2 years, respectively. Adverse drug reactions occurred in 16.5% and 13.8% of patients in the empagliflozin 10-mg and placebo groups, respectively, and the most common adverse drug reaction was urinary tract infection (10 mg, 3.1%; placebo, 2.4%).
No patients with eGFR <20 mL/min/1.73 m^2 or renal impairment requiring dialysis were enrolled in the clinical studies of patients with chronic heart failure. Thus, the package insert of empagliflozin states that the necessity of treatment should be carefully examined in patients with chronic heart failure with eGFR <20 mL/min/1.73 m^2. (During treatment, eGFR may decrease and renal impairment may be exacerbated.)

2.6. Significance of Conducting the Study

As mentioned above, the development of an effective treatment for fluid overload or heart

failure in PD patients may lead to improved outcomes in PD patients and continued PD treatment [16] associated with better health-related quality of life than HD. Among standard therapeutic drugs for heart failure, SGLT2 inhibitors attracted our attention. SGLT2 is expressed relatively specifically in the renal tubules, and the therapeutic efficacy of empagliflozin may be reduced as renal impairment progresses; however, advanced renal impairment is not associated with the reduced effect of empagliflozin in terms of improved outcomes, at least in patients with chronic heart failure with eGFR ≥20 mL/min/1.73 m^2. [17] Because SGLT2 is not expressed in cardiomyocytes, SGLT2 inhibitors acting on the kidneys are also expected to exert cardioprotective effects by reducing inflammation, oxidative stress, fibrosis, and sympathetic nervous system activation. [18] If this is true, SGLT2 inhibitors may also be effective in treating PD patients with loss of renal function. It has been recently reported that SGLT2 is expressed in the human peritoneum, and animal studies have shown that the administration of SGLT2 inhibitors results in an increased amount of water removed and inhibition of damage to the peritoneal tissue during PD. [19] [20] [21] These phenomena suggest that SGLT2 is involved in glucose transfer from peritoneal dialysate solutions to the body, which was previously thought to be diffusion primarily driven by a concentration gradient, and that SGLT2 inhibitors help alleviate glucotoxicity to the peritoneum. SGLT2 inhibitors are expected to improve outcomes in PD patients and help them continue to receive PD therapy.

Thus far, there have been only a limited number of reports on treatment with SGLT2 inhibitors in dialysis patients, and advanced renal impairment is not associated with increased frequency of adverse drug reactions, at least in patients with chronic heart failure with eGFR ≥20 mL/min/1.73 m^2. [17] In a large-scale study of predialysis CKD (DAPA-CKD trial: ClinicalTrials.gov Identifier NCT03036150), patients treated with SGLT2 inhibitors who initiated dialysis had a relative reduction in mortality of 21% when the subjects continued to receive SGLT2 inhibitors and placebo as the study drugs even after the initiation of dialysis. [22] In theory, urinary sugar decreases in PD patients because of the therapeutic efficacy of SGLT2 inhibitors, and hypoglycaemia, euglycaemic ketoacidosis, and genital infection, typical adverse drug reactions to SGLT2 inhibitors, are less likely to occur. [23] In addition to these backdrops, a recent overseas single-arm study of SGLT2 inhibitors in 50 PD patients with type 2 diabetes has reported an increased amount of water removed and increased urine volume compared with baseline after a 6-month intervention. [24] Approximately 250 PD patients are managed in the institution that made this report (the largest high-volume center if extrapolated to Japan); almost all PD patients with diabetes (approximately 70) have received SGLT2 inhibitors, and good results have been obtained, with no serious adverse drug reactions reported (personal communication with Prof. Abdullah Alhwiesh). An overseas RCT of SGLT2 inhibitors in subjects including PD patients (i.e., renal transplantation, predialysis CKD, and HD patients) is ongoing, using all-cause mortality, renal failure, and hospitalization for heat

failure as the primary composite endpoint, although the amount of water removed in PD does not appear to be the predetermined endpoint (ClinicalTrials.gov Identifier: NCT05374291). An open-label RCT in a small number of PD patients with type 2 diabetes is also ongoing in Japan using BNP and the amount of water removed as the primary and secondary endpoints, respectively (Protocol No.: jRCT1011210022). However, it is different from the study in that it is a double-blind study in patients with and without diabetes and uses a crossover design to enhance statistical power. The study is a multicenter, double-blind RCT that uses the amount of water removed, which is an important indicator for PD patients and related to outcomes, as the primary endpoint. [25] [26]

3. Objective of the Study and Endpoints

3.1. Objective of the Study

The study aims to explore the effect of empagliflozin on the amount of water removed and its safety in patients with heart failure on PD using a placebo as a comparator.

3.2. Primary Endpoint

Change in the amount of water removed per day to the glucose peritoneal dialysate solutions at Week 8 (The amount of water removed per day is calculated as the mean of all results for 5 of 7 consecutive days, except for the day when the maximum and minimum values were recorded)

[Rationale for the primary endpoint]
The amount of water removed, which has been reported to be associated with outcomes in PD patients, [25] [26] is selected as the primary endpoint, and the mean of all results for 5 days, except for the day when the maximum and minimum values are recorded, is to be calculated with variation in mind.

3.3. Secondary Endpoints

(1) Changes in NT-proBNP and BNP
(2) Changes in FAST PET-related factors: Amount of water removed, sodium, potassium, glucose, urea nitrogen, creatinine, uric acid, protein, IL-6, and CA-125 and drainage-to-serum creatinine ratio
(3) Changes in factors related to 24-hour urine collection: Urine volume, sodium, potassium, glucose, urea nitrogen, creatinine, uric acid, urine protein, and urea and creatinine clearance (the mean of urea and creatinine clearance)
(4) Urine KIM-1
(5) Changes in anemia-related factors: Hemoglobin, hematocrit, ferritin, and transferrin

saturation

(6) Changes in body weight and blood pressure
(7) Changes in body composition: Intracellular and extracellular fluid volume, Fluid overload
(8) Adverse events

[Rationale for the secondary endpoints]

(1) NT-proBNP and BNP are markers of heart failure and are selected to evaluate the condition of heart failure.
(2) This endpoint is selected to determine whether treatment with SGLT2 inhibitors results in the inhibition of glucose absorption in the peritoneal dialysate solutions and whether it affects the movement of sodium and other solutes. IL-6 and CA-125 are to be measured to evaluate the effects on peritoneal tissue fibrosis, inflammation, and mesothelial cells.
(3) Because treatment with SGLT2 inhibitors is associated with increased urine volume and changes in solute excretion in nondialysis patients, this endpoint is selected to determine whether a similar effect can be obtained in PD patients with loss of renal function.
(4) Because treatment with SGLT2 inhibitors is associated with decreased KIM-1, a renal tubular disorder biomarker, in nondialysis patients, this endpoint is selected to determine whether a similar effect can be obtained in PD patients with loss of renal function.
(5) This endpoint is selected because treatment with SGLT2 inhibitors has shown to improve anemia and iron utilization efficiency in nondialysis patients.
(6) This endpoint is selected because treatment with SGLT2 inhibitors has shown to reduce body weight and blood pressure in nondialysis patients.
(7) This endpoint is selected because treatment with SGLT2 inhibitors is associated with changes in body fluid composition in nondialysis patients.
(8) Adverse events are selected to evaluate the safety of empagliflozin.

4. Study Design

4.1. Study Design

This is a multicenter, randomized, double-blind, crossover study consisting of the following two groups:

(1) Preemptive empagliflozin group

(2) Preemptive placebo group

The study participants will be randomized using a permuted block method stratified by the study site.

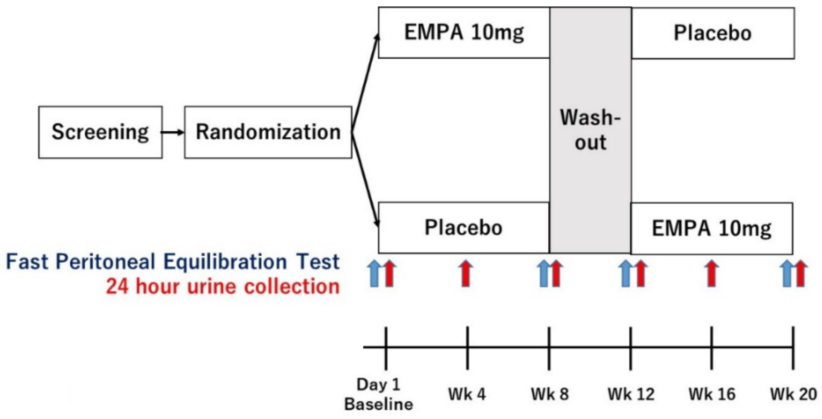

[Rationale for the study design]

The study participants will be randomly assigned to minimize the variation in patient's demographics between the two groups and enhance comparability. Because empagliflozin and the placebo may have a short shelf life, a permuted block method stratified by the study site will be used so that the drugs can be administered smoothly. A double-blind approach will be used because physicians and subjects having knowledge of the treatment group assignments for reasons such as drain time duration adjustment may affect the evaluation results. The target disease is a chronic condition in the study, and a crossover design will be used to increase the detection power.

The placebo will be selected as a comparator because the study is intended to determine whether the amount of water removed will increase when empagliflozin is used.

4.2. Planned Number of Study Participants in the Study

A total of 36 subjects to be enrolled as study participants

Preemptive empagliflozin group: 18 subjects

Preemptive placebo group: 18 subjects

[Rationale for the planned number of study participants]

In our preliminary data, five PD patients treated with empagliflozin 10 mg/day experienced a mean increase in daily ultrafiltration volume (UFV) of 90 mL after one month. This increased UFV of 90 mL/day was associated with an 18% reduction in the risk of death, suggesting a clinically significant change. [26] Assuming a conservative standard deviation of 150 mL for changes in UFV, based on data from a cohort of 46 PD patients in the Department of Nephrology at Osaka University (where the observed standard deviation was 114 mL), and a within-subject to between-subject standard deviation ratio of 1:1, we estimated that to detect a 90 mL/day increase in UFV with empagliflozin 10 mg, using an alpha error of 0.05 and a statistical power of 0.90, 15 subjects per group would be required (two-sample t-test). To allow for potential dropouts, a total of 18 subjects per group was planned.

4.3. Expected Duration of the Study

(1) Expected duration of the study

The jRCT publication date to December 31, 2025

(2) Expected duration of enrollment

The jRCT publication date to May 31, 2024

(3) Expected duration of observation

The of jRCT publication date to October 31, 2024

5. Selection of Study Participants

Patients who meet all the inclusion criteria and do not satisfy any exclusion criterion will be enrolled as study participants.

5.1. Inclusion Criteria

(1) Individuals aged between 18 and 90 years at the time of providing informed consent
(2) Individuals with BNP ≥40 pg/mL, NT-proBNP ≥400 pg/mL, structural heart disease (left atrial enlargement and/or left ventricular hypertrophy), increased ventricular filling pressure, or a history of hospitalization due to heart failure. Reference values for NT-proBNP should be used for patients using ARNIs instead of those for BNP.
(3) Individuals who have received standard drug therapy for heart failure (at least one of the following: loop diuretics, ACEIs, ARBs, ARNIs, beta-blockers, or MRAs)
(4) Individuals who have undergone peritoneal dialysis for at least 3 months
(5) Individuals who use at least 3 L of glucose peritoneal dialysate solutions per day
(6) Individuals who can provide a written informed consent to participate in the study

Definition of structural heart disease (left atrial enlargement and/or left ventricular hypertrophy) and ventricular filling pressure [13]

Left atrial enlargement	LA width ≥4.0 cm, or<br>LA length ≥5.0 cm, or<br>LA area ≥20 cm^2, or<br>LA volume (LAV) ≥55 mL, or<br>LA volume index (LAVI) ≥34 mL/m^2
Left ventricular hypertrophy	Interventricular septal thickness (IVST) or left ventricular posterior wall thickness (LVPWth) ≥1.1 cm, or<br>LV mass index (LVMI) ≥115 g/m^2 (men) and ≥95 g/m^2 (women)
Ventricular filling pressure	E/e′ (mean septal and lateral) ≥13, or<br>e′ (mean septal and lateral) <9 cm/s

[Rationale for the inclusion criteria]
Criteria (1), (2), (3), and (4) were established to select study participants suitable for the efficacy assessment of empagliflozin. Criterion (5) was established because an increased amount of water removed, which formed the basis for sample size determination, was calculated from individuals who used at least 3 L of glucose peritoneal dialysate solutions per day. Criterion (6) was established to conduct the study in study participants who provided an informed consent appropriately.

5.2. Exclusion Criteria

(1) Individuals who are using or have used SGLT2 inhibitors within the past 3 months
(2) Individuals who are not expected to live for 1 year or more after enrollment
(3) Individuals who receive hybrid therapy comprising peritoneal dialysis and hemodialysis
(4) Individuals who have or have had peritonitis within the past 2 months
(5) Women who are pregnant or nursing
(6) Individuals who have an uncontrollable infection
(7) Individuals who participate in clinical studies (trials and research) involving other interventions
(8) Individuals disqualified from participation in the study by the investigator or subinvestigator for any other reasons

[Rationale for the exclusion criteria]
Criteria (1) to (4) were established because these factors might affect the efficacy evaluation. Criteria (5) and (6) were established to ensure the safety of the study participants. Criterion (7) was established because simultaneous entry into a clinical study involving intervention might affect the endpoints of the study and safety evaluation. Criterion (8) was established so that the investigator or subinvestigator could exclude individuals who were considered ineligible for the study for reasons other than those mentioned above.

6. Drugs to be Studied

6.1. Summary of the Drugs to be Studied

In the study, the following study drugs (an investigational product and a control drug) will be used:

6.1.1. Investigational Product

Classification of drugs (unapproved, off-label, or approved) in the Law on Drugs, Medical Devices, etc.		□ Unapproved □ Off-label ■ Approved
Generic name (Enter the development code if unapproved in Japan and overseas.)		Empagliflozin 10 mg
Brand name (For an overseas product, enter the country as well.)		Jardiance Tablets 10 mg
Name of the marketing authorization holder		Nippon Boehringer Ingelheim Co., Ltd.
Approval No.		22600AMX01387000
Dosage form		Film-coated tablets
Storage method		Store at room temperature
Investigational product provider	Name	Boehringer Ingelheim Pharma GmbH & Co. KG
	Address	Birkendorfer Strasse 65 88397 Biberach an der Riss, Germany

6.1.1. Control Drug

Classification of drugs (unapproved, off-label, or approved) in the Law on Drugs, Medical Devices, etc.		■ Unapproved □ Off-label □ Approved
Generic name (Enter the development code if unapproved in Japan and overseas.)		Placebo tablets
Dosage form		Film-coated tablets<br>(Appearance etc. are the same as the test drug and cannot be identified)
Storage method		Store at room temperature
Investigational product provider	Name	Boehringer Ingelheim Pharma GmbH & Co. KG
	Address	Birkendorfer Strasse 65 88397 Biberach an der Riss, Germany

6.2. Assurance of Quality of the Drugs to be Studied

The principal investigator will be responsible for the following tasks:

1 Preparing or obtaining and retaining documents describing the ingredients, quantities, specifications, and study methods as well as properties and structure of the drugs manufacturing methods, packaging and labeling of the drugs method of use in clinical studies, and other necessary information.

2 Keeping and maintaining a record of the manufacturing date and number or code and other manufacturing information on the drugs.

3 Keeping and maintaining a record of the quantity of the obtained drugs and the date when they were obtained.

4 Verifying the information about the poor quality of the drugs used in clinical studies, if any, and reporting measures, such as the interruption of the studies, to the Certified Review Board. The principal investigator will also keep a record of it.

5 If the principal investigator considers it necessary to recall the drugs for reasons such as poor quality of the drugs used in clinical studies, the principal investigator will promptly report to the Certified Review Board and perform the following:

(a) Promptly inform the investigator of discontinuation and recall of the drugs.

(b) Prepare and retain a recall record describing the description of the recall, results of the investigation of the cause, and corrective action.

The investigator will be responsible for the following items:

1 Keeping and maintaining a record of the quantity of the used drugs and the date when they were used for each study participant.

2 Keeping and maintaining a record of the quantity of the discarded drugs and the date when they were discarded.

3 Upon receipt of the notification from the principal investigator, the investigator will promptly instruct the subinvestigator and other personnel to discontinue and recall the drugs.

7. Method of Application of Drugs to Study Participants (protocol treatment)

7.1. Method of Application of Drugs to Subjects

7.1.1. Preemptive Empagliflozin Group

Empagliflozin is orally administered at a dose of 10 mg once daily before or after breakfast for 8 weeks and after a washout period of 4 weeks; the placebo is orally administered once daily before or after breakfast for 8 weeks.

7.1.2. Preemptive Placebo Group

The placebo is orally administered once daily before or after breakfast for 8 weeks and after a washout period of 4 weeks; empagliflozin is orally administered at a dose of 10 mg once daily before or after breakfast for 8 weeks.

7.1.3. Rationale for the Method of Application of Drugs to Subjects

The study drugs will be administered according to the dosing regimen of empagliflozin approved for chronic heart failure. Because our preliminary examination revealed that it might take approximately 2 or 3 weeks to achieve an increase in the amount of water removed in some subjects, the duration of treatment was set at 8 weeks, allowing a margin for the duration. A washout period was set at 4 weeks in consideration of the period when the carryover effects would disappear in the preemptive empagliflozin group and frequency of hospital visits by study participants. The FDA's Guideline for Bioequivalence Studies of Generic Products recommends that the washout period should be at least 5 times the elimination half-life of the given drug in a crossover study. A 4-week washout period would suffice because the elimination half-life following multiple oral administration of empagliflozin 10 mg once daily for 28 days was 18 hours. The mean half-life of empagliflozin 50 mg following single administration in 8 patients with end-stage renal failure was 22 hours. [27]

7.2. Concomitant Drugs and Therapies

A standard of care for chronic heart disease will be provided. Peritoneal dialysis will be conducted according to procedures similar to those used before study participation, and changes in the peritoneal dialysis prescription are prohibited during the study as a general rule. Concomitant drugs or therapies will be administered to treat comorbidities as routine medical practice.

7.3. Prohibited Concomitant Drugs

The use of SGLT2 inhibitors other than empagliflozin is prohibited during protocol treatment.

[Rationale for the prohibited concomitant drugs]
Concomitant use of SGLT2 inhibitors other than empagliflozin was prohibited because of their potential effects on the efficacy evaluation in the study.

8. Observation, Test, and Evaluation at Each Time Point

The investigator or subinvestigator will collect data according to the observation, test, evaluation schedule outlined as follows.
In principle, the same investigator or subinvestigator will be responsible for the observation, test, and evaluation of study participants.

8.1. Observation, Test, and Evaluation Schedule

8.1.1. At Enrollment

After providing informed consent, the study participants will be determined according to the inclusion and exclusion criteria and enrolled if they are considered eligible for the study. For inclusion criterion (2), BNP and NT-proBNP data collected from routine medical practice within 182 days of providing informed consent may be used for assessment, and for echocardiography used to evaluate structural heart disease, data collected from routine medical practice within 365 days of providing informed consent may be used for assessment.

- Written informed consent
- BNP and NT-proBNP (if necessary)
- Echocardiography (if necessary)
- Enrollment and assignment

8.1.2. At the Start of the Treatment (Visit 1)

- Study participants' demographics
- Comorbidities
- Amount of water removed per day
- BNP and NT-proBNP
- FAST PET
- 24-hour urine collection
- Hematology and blood biochemistry
- Body weight and blood pressure
- Body composition
- Adverse events
- Concomitant drugs

8.1.3. Week 4 (Visit 2)

- Amount of water removed per day
- BNP
- 24-hour urine collection

- Hematology and blood biochemistry
- Body weight and blood pressure
- Adverse events
- Concomitant drugs

8.1.4. Week 8 (Visit 3)

- Amount of water removed per day
- BNP and NT-proBNP
- FAST PET
- 24-hour urine collection
- Hematology and blood biochemistry
- Body weight and blood pressure
- Body composition
- Adverse events
- Concomitant drugs

8.1.5. Week 12 (Visit 4)

- Amount of water removed per day
- BNP and NT-proBNP
- FAST PET
- 24-hour urine collection
- Hematology and blood biochemistry
- Body weight and blood pressure
- Body composition
- Adverse events
- Concomitant drugs

8.1.6. Week 16 (Visit 5)

- Amount of water removed per day
- BNP
- 24-hour urine collection
- Hematology and blood biochemistry
- Body weight and blood pressure
- Adverse events
- Concomitant drugs

8.1.7. Week 20 (Visit 6)

- Amount of water removed per day
- BNP and NT-proBNP
- FAST PET
- 24-hour urine collection
- Hematology and blood biochemistry
- Body weight and blood pressure
- Body composition
- Adverse events
- Concomitant drugs

8.1.8. At Discontinuation

- Amount of water removed per day
- Hematology and blood biochemistry (if necessary)
- BNP and NT-proBNP (if necessary)
- Body weight and blood pressure (if necessary)
- Adverse events

8.2. Study Calendar

		Period 1			Washout 4 weeks	Period 2			
	At enrollment	At the start of the treatment	Week 4	Week 8		Week 12	Week 16	Week 20	At discon-tinuation
Visit	0	1	2	3		4	5	6	
Day		1 (day1)	29	57		85 (day1)	113	141	
Acceptable range (days)		-	±7	±14		Date of study visit at Week 8 + 28 (−7, +14)	Date of study visit at Week 12 + 28 (±7)	Date of study visit at Week 12 + 56 (±14)	
Informed consent	●								
Enrollment and assignment	●								
Study participants' demographics		●							
Comorbidities		●							
Concomitant drugs		←	—	—	—	—	—	—	→
Study drug administration		←	—	→		←	—	→	
Amount of water removed per day		←	—	→		←	—	→	●
NT-proBNP	○*	●		●		●		●	○
BNP	○*	●	●	●		●	●	●	○
Echocardiography	○**								
FAST PET		●		●		●		●	
24-hour urine collection***		●	●	●		●	●	●	
Hematology and blood biochemistry		●	●	●		●	●	●	○
Body weight and blood pressure		●	●	●		●	●	●	○
Body composition		●		●		●		●	
Adverse events		←	—	—	—	—	—	—	→

●, required; ○, if necessary

*; Data collected from routine medical practice 182 days before providing informed consent may be used for assessment.

**: Data collected from routine medical practice 365 days before providing informed consent may be used for assessment.

***: Urine KIM-1 is measured only on Visit 3 and Visit 6.

9. Observation, Test, and Evaluation Procedures

9.1. Informed Consent

The investigator or subinvestigator will obtain informed consent from the study participants according to the steps described in Section 15.4.1.

9.2. Enrollment and Assignment

9.2.1. Enrollment and Assignment Procedures

The investigator or subinvestigator will enroll study participants in the study according to the following procedures:

(1) The investigator or subinvestigator will enter the necessary information about the patients who provided consent on the Web-based registration system for enrollment.
The research collaborator may also be able to enter the information on the Web-based registration system for the investigator or subinvestigator under the instruction of the investigator or subinvestigator.

(2) The patient's eligibility will be assessed on the Web-based registration system, and if considered eligible, the patient will be randomized and his/her treatment group will be determined.

(3) The investigator or subinvestigator will review the enrollment assessment and randomization results on the Web-based registration system.

(4) The investigator or subinvestigator will initiate treatment to which the study participant has been assigned since enrollment.

9.2.2. Contact Information About Enrollment Procedures

Help Desk, intellim Corporation
Office hours: 9:30–17:00 from Monday to Friday (except Saturday, Sunday, holidays, and New Year holidays)
*For the details of the study, including the inclusion and exclusion criteria, please contact the principal investigator.

9.2.3. Creation and Retention of a Randomization Schedule

The person responsible for randomization will develop randomization procedures and manage the randomization information about study participants.
The randomization information should be stored in a safe location, and the investigator and subinvestigator will not be allowed to have access to the information.
Key code breaking for the entire study will be performed after the study is completed and the data are fixed for analysis. Until then, they will remain blinded.

9.2.4. Emergency Key Code Breaking

(1) Emergency key code breaking is only permitted in the event of an emergency when a study participant develops a serious adverse event, and it becomes necessary to identify the treatment provided to the study participant so that he/she can receive appropriate medical treatment.

(2) When emergency key code breaking is needed, the investigator for the study participant may be able to break the randomization code on his/her own responsibility but must first consider safety. The investigator will log in to the EDC system and break the key code. The work description will be recorded and stored on the system.

9.3. Study Treatment Status

The first day of study drug administration and the last day of treatment will be reviewed, and the treatment compliance will be monitored and classified into either ≥80% or <80% in each period.

9.4. Study Participants' Demographics

The following survey parameters and endpoints will be examined:

- Study participant identification code
- Date of providing informed consent
- Date of birth
- Sex
- Height
- Primary renal disease (chronic glomerulonephritis, diabetic nephropathy, nephrosclerosis, and others)
- NYHA class
- First day of peritoneal dialysis

9.5. Comorbidities

The presence or absence of comorbid diabetes, coronary artery disease, and atrial fibrillation will be examined at the time of providing informed consent.

9.6. Concomitant Drugs

Concomitant drugs (drug name, route of administration, dose, duration of treatment, and reason for coadministration) used to treat chronic heart failure, anemia, diabetes, and uric acid will be investigated.

Major Category	Subcategory
Chronic Heart Failure-related Drugs (Antihypertensive Diuretics)	ACE Inhibitors
	ARB
	ARNI
	β-blockers
	Diuretics (Loop/Thiazide/Tolvaptan)
	Mineralocorticoid Receptor Antagonists
	CCB
Anemia-related Drugs	Iron Supplements
	Erythropoiesis-stimulating Agents
	HIF-PH Inhibitors
Diabetes-related Drugs	Insulin
	GLP-1 Receptor Agonists or GIP/GLP-1 Receptor Agonists
	DPP-4 Inhibitors
	Glinide Drugs
	α-Glucosidase Inhibitors
	Biguanide Drugs/Imeglimin
	Sulfonylurea Drugs
	Thiazolidinedione Drugs
Uric Acid-related Drugs	Febuxostat

9.7. Amount of Water Removed Per Day

Whenever a peritoneal dialysate solution bag is replaced, the study participant will determine the weight of the drainage to calculate the amount of water removed in each dialysis session (by subtracting the weight of the infused solution from that of the drainage). The amount of water removed per day will be calculated by adding the amount of water removed in each bag replacement. The study participant will record the amount of water removed per day on the notebook provided by the study site. The amount of icodextrin-containing peritoneal dialysates removed should be excluded.

The collection period for the amount of water removed will be the consecutive 7 days of peritoneal dialysis performed most recently before the visit.
Check the peritoneal dialysate solutions (name of the dialysate solution, volume of infusion, and duration of dialysate exposure).

9.8. BNP and NT-proBNP

Blood will be collected from the study participant during a hospital visit, and BNP will be measured according to ordinary procedures used by each study site. NT-proBNP will be measured by H.U. Frontier, Inc. (SRL)

9.9. Frequently and Short-time Peritoneal Equilibration Test (FAST PET)

After 2 L of peritoneal dialysate solutions (or equivalent osmotic dialysate) equivalent to 2.27% glucose concentration are retained for 4 hours, the dialysate solutions will be drained to measure the amount of water removed and concentrations of sodium, potassium, glucose, urea

nitrogen, creatinine, uric acid, protein, IL-6, and CA-125 in the drainage. The drainage-to-serum creatinine ratio will also be determined after the addition of serum creatinine. IL-6 and CA-125 will be measured by H.U. Frontier, Inc. (SRL), and other analytes will be determined according to ordinary procedures used by each study site.

9.10. 24-hour Urine Collection

The study participant will collect 24-hour urine samples using a 24-hour urine collector (Urinemate P) or his/her own container and bring them to an outpatient appointment. The collected 24-hour urine will be used to measure urine volume, sodium, potassium, glucose, urea nitrogen, creatinine, uric acid, urine protein, and urine KIM-1. Urea and creatinine clearance (the mean of urea and creatinine clearance), coupled with blood data, will be calculated. Urine KIM-1 will be measured by LSI Medience Corporation, and the other analytes will be determined according to ordinary procedures used by each study site.

9.11. Hematology and Blood Biochemistry

Blood will be collected from the study participant during a hospital visit and determined according to ordinary procedures used by each study site.

Required	Hemoglobin, hematocrit, ferritin, transferrin saturation, urea nitrogen, creatinine, uric acid, albumin, AST, ALT
If necessary	sodium, potassium, HbA1c, total bilirubin

9.12. Body Weight and Blood Pressure

Body weight and blood pressure will be measured using a scale and a sphygmomanometer installed in the outpatient department in each study site. Body weight should be measured after the peritoneal dialysate solutions are drained completely. Blood pressure will be measured twice in a sitting position, and its mean will be used.

9.13. Body Composition

Intracellular and extracellular fluid volume and fluid overload will be determined by bioimpedance methods installed in the outpatient department in each study site. Fluid overload should be monitored as much as possible.

9.14. Criteria for Discontinuation and Termination in Each Study Participant

9.14.1. Discontinuation for Each Study Participant

The investigator or subinvestigator will discontinue the study participant from participating in

the study when he/she was found to meet the following criteria after enrollment:

(1) When an adverse event occurred and the investigator or subinvestigator considers that continued participation in the study would impose an unacceptable risk on the study participant
(2) When the study participant requested study discontinuation
(3) When the prohibited concomitant drugs were used
(4) When the study participant is found to be ineligible for the study
(5) When the study participant completely stopped peritoneal dialysis due to full transition to hemodialysis or renal transplantation
(6) When the study participant developed peritonitis
(7) When it turned out that the study participant was unable to complete the necessary observations and tests for his/her personal reasons, including change of residence
(8) When the investigator or subinvestigator considers that the intervention should be discontinued

9.14.2. Discontinuation Procedures for Each Study Participant

When the study participant was found to meet the discontinuation criteria, the investigator or subinvestigator will immediately inform the participant accordingly and take measures such as alternate treatment as needed.
The investigator or subinvestigator will conduct the protocol-specified observation, test, and evaluation as much as possible and record the results, with the reason for discontinuation, on the case report form.

9.14.3. Termination for Each Study Participant

When all protocol-specified observations, tests, and evaluations are completed, the study will be terminated for the study participant.

10. Adverse Events

10.1. Definition of Adverse Events

An adverse event is any unfavorable and unintended sign (including an abnormal laboratory finding), symptom, or disease in a study participant administered a medicinal product, whether related or unrelated to the study. A symptom present before the start of the study and its worsening after administration will also be handled as an adverse event.

10.2. Survey Period for Adverse Events

The collection of adverse drug information will begin on the first day of study drug administration and continue until study drug discontinuation or the last day of study drug administration.

10.3. Assessment of Adverse Events

10.3.1. Investigation of Subjective Symptoms and Objective Findings

The investigator or subinvestigator will investigate objective symptoms and ask the study participant to explain whether he/she has experienced subjective symptoms.
When the study participant receives outpatient services, the investigator or subinvestigator will question him/her about the objective symptoms and the subjective symptoms that developed when he/she did not visit the study site.

10.3.2. Abnormal Changes in Laboratory Values

The criteria for normal laboratory values are based on the normal range (from upper to lower limits) in the study site. For test parameters for which no normal range is available, the investigator or subinvestigator will determine if they are normal or abnormal.
The investigator or subinvestigator will assess laboratory values for abnormal changes by comparing them with baseline values (or laboratory values measured before enrollment if no baseline values are available) or the laboratory values measured after study drug administration.

10.4. Evaluation of Adverse Events

For the adverse events observed during the survey period, the investigator or subinvestigator will record the following items on the case report form. When more than one adverse event was observed, each event should be recorded.

(1) Name of the adverse event

(2) Date of onset
(3) Severity
(4) Seriousness (nonserious or serious)
(5) Causal relationship with the study
(6) Causal relationship with the study drug
(7) Treatment
(8) Date of outcome
(9) Outcome

10.4.1. Name of the Adverse Event

For each event, its diagnosis should be provided. Accompanying signs (including abnormal laboratory and ECG findings) and symptoms of the diagnosis should not be provided as other adverse events. If the diagnosis is unknown, its sign or symptom may be provided as an adverse event as appropriate.

10.4.2. Date of Onset

The onset date of an adverse event will be determined according to the following criteria:

Adverse event	Date of onset
Signs, symptoms, or disease (diagnosis)	Enter the date when the study participant or investigator or subinvestigator was aware of the signs or symptoms of the adverse events for the first time.
Asymptomatic disease	Enter the date when a test was conducted for diagnostic purpose and diagnosis was confirmed.<br>Enter the date of diagnosis confirmed even when obsolete findings can be seen or the date of onset can be roughly estimated on the basis of laboratory findings.
Worsening of comorbidities	Enter the date when the study participant or investigator or subinvestigator was aware of the worsening of the disease or symptoms for the first time.
Abnormalities found via a test after the application of the drugs to be studied	Enter the test date when clinically significant laboratory abnormalities were observed.
Abnormalities that were found via test at the start of the application of the drugs to be studied and worsened in subsequent tests	Enter the test day when laboratory values, which apparently elevated, declined, increased, or decreased from a medical judgment, were observed.

10.4.3. Severity

The severity of an adverse event will be classified as follows:

Mild	Transient and easily tolerated by the subject
Moderate	Results in interruption of the subject's usual activities
Severe	Results in considerable interference with the subject's usual activities

10.4.4. Seriousness

Adverse events that meet any of the following conditions will be considered serious, regardless of their severity:

(1) result in death
(2) are life-threatening
(3) require inpatient hospitalization or prolongation of existing hospitalization
(4) result in disability
(5) may be incapacitating
(6) are serious next to the aforementioned conditions
(7) result in congenital disease or abnormality in the next generation
(8) are other medically significant events or reactions

However, serious adverse events do exclude hospitalizations unrelated to adverse events, such as hospitalization for treatment scheduled before the start of the study and that required for a test.

"Other medically significant condition" in (8) in this section refers to an event that may jeopardize the patient or study participant based on appropriate medical judgment and requires medical and surgical interventions to prevent serious adverse events defined in (1) to (7) from occurring.

The term "life-threatening" in this section refers to any reaction that places the patient at immediate risk of death from the aforementioned reaction as it occurred; i.e., it excludes a reaction that, had it occurred in a more severe form, might have caused death.

Important medical events that may not immediately result in death, be life-threatening, or require hospitalization may be considered a serious adverse reaction when, based on appropriate medical and scientific judgment, they may jeopardize the patient and may require intervention to prevent one of the outcomes listed in the aforementioned definitions (1) to (7). Examples of such medical events include allergic bronchospasm requiring intensive treatment in an emergency room or at home, blood dyscrasias or convulsions that do not result in inpatient hospitalization, or the development of drug dependency or abuse. Suspected cases of infection via pathogenic agents may also be considered a serious adverse drug reaction.

10.4.5. Causal Relationship with the Study

The causal relationship between the conduct of the study and adverse event will be classified according to the following table. When the adverse drug reaction was considered unrelated to the study, the reason for assessment will be recorded on the case report form.

Related	There is a clear temporal correlation (including clinical course after discontinuation) with study drug administration. Although other factors, such as primary disease, comorbidities, and concomitant drugs and therapies, may contribute to the adverse event, it can also be explained by the application of the drugs to be studied.
Unrelated	There is no clear temporal correlation with the application of the drugs to be studied, or the adverse event can be fully explained by other factors, such as disease, comorbidities, and concomitant drugs and therapies.

10.4.6. Causal Relationship with the Study Drug

The causal relationship between the study drug and adverse event will be classified and recorded in a similar way to that described in Section 10.4.5.

10.4.7. Date of Outcome

The date of outcome is defined as the day when the objective symptoms and the subjective symptoms are resolved, or the outcome of an adverse event was assessed. If the outcome is "death," the date of death will be regarded as the date of outcome.

10.4.8. Outcome

The outcome of an adverse event will be classified as follows:

Classification	Criteria
Recovered or resolved	• Successful resolution or recovery of symptoms or findings<br>• Normalization of laboratory values or their recovery to baseline levels
Recovering or Resolving	• Symptoms or findings are nearly resolved<br>• Laboratory values improved without being normalized or recovered to baseline levels<br>• The adverse event is not the immediate cause of death, and although recovering from it, the patient died

Classification	Criteria
Unrecovered or unresolved	• Symptoms, findings, or laboratory values remain unchanged<br>• The symptoms, findings, or laboratory values on the last day of observation are worse than those first reported<br>• Irreversible congenital anomalies<br>• The adverse event is not the immediate cause of death, and the patient died without being recovered from it
Recovered or resolved with sequelae	• Substantial disruption of the ability to conduct normal life functions
Fatal or death	• The adverse event was directly related to death<br>"Being directly related" means that the adverse event caused death or it apparently contributed to death.<br>• The outcome of the adverse event that is not considered (determined or estimated) the direct cause of death in the same patient should not be regarded as death
Unknown	• The clinical course on or after the date of onset could not be followed in the same way as the protocol for reasons such as patient transfer or change of residence

10.5. Measures Taken for the Adverse Events

10.5.1. Treatment Provided to Study Participants

In case an adverse event occurs, the investigator and subinvestigator will provide appropriate emergency treatment to ensure the safety of the study participant and resolve concerns and investigate the cause by requesting a specialist's diagnosis and treatment as needed.

10.5.2. Follow-up Study of Adverse Events

The adverse events that occurred during the study should be monitored as much as possible until they have resolved or do not need to be monitored clinically. The investigator or subinvestigator will determine the follow-up methods, such as inpatient or outpatient services and frequency of hospital visits and test parameters for outpatients, according to the type and degree of the adverse event.

10.5.3. Reporting to the Financial Contributor

In case an adverse event occurs, the investigator or subinvestigator will report it according to

the procedures specified by Nippon Boehringer Ingelheim Co., Ltd. providing funds. The report shall not contain any study participant's personally identifiable information.

10.6. Adverse Events Expected to Occur During the Study

10.6.1. Information Provided on the Package Insert of Empagliflozin

The following adverse drug reactions are described in the package insert of empagliflozin (revised in April 2022 [3rd version]). The investigator or subinvestigator should check the latest package insert when administering the study drug.
Patients with eGFR <20 mL/min/1.73 m^2 or renal impairment requiring dialysis are excluded from the clinical studies mentioned in the package insert.

(1) Clinically significant adverse drug reactions (incidence)
Hypoglycaemia (1.8%), dehydration (0.3%), ketoacidosis (incidence unknown), pyelonephritis (<0.1%), necrotizing fasciitis genital and perineal (Fournier's gangrene) (<0.1%), and sepsis (0.1%)

(2) Other adverse drug reactions

Incidence / Classification	0.1~5%	<0.1%
Infection	Urinary tract infection, cystitis, vulvovaginal candidiasis, and asymptomatic bacteriuria	Vulvovaginitis, vaginitis bacterial, and trichomoniasis
Reproductive system disorders	Balanoposthitis, pruritus genital, and balanitis	Vulvovaginal pruritus and discomfort
Metabolism and nutrition disorders	Hyperlipidaemia	Body fluid volume decreased
Blood and lymphatic system disorders		Haemoconcentration
Nervous system disorders	Dizziness	Taste abnormality
Gastrointestinal disorders	Constipation	Abdominal distension
Skin and subcutaneous tissue disorders	Pruritus	Rash, eczema, and urticaria
Renal and urinary disorders	Pollakiuria, polyuria, dysuria, and urine output increased	Micturition urgency
General disorders	Thirst and feeling hungry	
Investigations	Weight decreased	Urine and blood ketone body present

10.6.2. Adverse Events in Patients with Renal Impairment

As precautions for patients with chronic heart failure and severe renal impairment, the package insert (revised in April 2022 [3rd version]) of empagliflozin states that “the necessity of treatment should be carefully examined in patients with chronic heart failure with eGFR <20 mL/min/1.73 m^2 because eGFR may decrease during treatment and renal impairment may be exacerbated.”

Conversely, the occurrence of adverse events by baseline eGFR in a phase 3 global study of patients with HFrEF (those with eGFR <20 mL/min/1.73 m^2 or renal impairment requiring dialysis are excluded) is summarized in the following table, [28] and the incidence of renal impairment in the empagliflozin group was similar to that in the placebo group, irrespective of baseline eGFR.

Baseline eGFR		Placebo group	Empagliflozin group
≥90 mL/min/1.73 m^2		N = 220	N = 229
	All adverse events	156 (70.9)	155 (67.7)
	Serious adverse events	94 (42.7)	72 (31.4)
	Renal impairment	13 (5.9)	7 (3.1)
60 to <90 mL/min/1.73 m^2		N = 738	N = 740
	All adverse events	560 (75.9)	548 (74.1)
	Serious adverse events	343 (46.5)	278 (37.6)
	Renal impairment	62 (8.4)	54 (7.3)
45 to <60 mL/min/1.73 m^2		467	344
	All adverse events	361 (77.3)	344 (79.4)
	Serious adverse events	225 (48.2)	188 (43.4)
	Renal impairment	49 (10.5)	39 (9.0)
30 to <45 mL/min/1.73 m^2		N = 348	N = 345
	All adverse events	307 (88.2)	265 (76.8)
	Serious adverse events	183 (52.6)	159 (46.1)
	Renal impairment	52 (14.9)	50 (14.5)
<30 mL/min/1.73 m^2		N = 89	N = 115
	All adverse events	78 (87.6)	108 (93.9)
	Serious adverse events	50 (56.2)	75 (65.2)
	Renal impairment	16 (18.0)	25 (21.7)

Number of subjects with adverse events (incidence, %)

In the single-arm study in 50 PD patients with type 2 diabetes treated with SGLT2 inhibitors conducted by King Fahd Hospital of the University, Imam Abdulrahman Bin Faisal University, almost all PD patients with diabetes (approximately 70) received SGLT2 inhibitors and had favorable outcomes without serious adverse drug reactions (personal communication with Prof. Abdullah Alhwiesh). In our study, 11 PD patients received empagliflozin 10 mg during routine medical practice and 2 of them discontinued treatment due to skin eruption and itching; however, no serious adverse events were reported.

10.7. Adverse Event of Special Interest (AESI)

An AESI is a preidentified adverse event of concern with prospective safety monitoring and evaluation in the project level of the study, and AESIs include potential adverse events that can be predicted from experience with similar drugs. AESIs must be reported to the Pharmacovigilance Department of Nippon Boehringer Ingelheim Co., Ltd. within the same deadline as that for serious adverse events.

The study participants with AESIs should be followed up properly, whatever the source of clinical data (such as central and in-hospital laboratory). When the study participants receive concomitant therapy, the investigator or subinvestigator must determine what concomitant therapy should be discontinued during the study. The discontinued concomitant therapy may be resumed at the discretion of the investigator or subinvestigator.

The following events will be considered an AESI:

a. Liver or drug-induced liver injury

A change in any of the following hepatic parameters is defined as serious drug-induced liver injury requiring follow-up:

1. an elevation in AST or ALT levels to at least 3 times the upper limit of the normal range and total bilirubin ≥2 times the upper limit of the normal range in blood samples measured on the same day or within 30 days of study visit
2. an elevation in AST or ALT levels to at least 3 times the upper limit of the normal range and INR ≥1.5 times the upper limit of the normal range in blood samples measured on the same day or within 30 days of study visit
3. an elevation in AST or ALT levels to at least 3 times the upper limit of the normal range and new onset or worsening of fatigue, feeling queasy, vomiting, right upper quadrant pain or tenderness, fever, rash, or eosinophilia (≥5%)
4. an elevation in AST or ALT levels to at least 5 times the upper limit of the normal range

The aforementioned findings correspond to a liver injury alert and patients with laboratory abnormalities will be followed up according to the DILI checklist provided by Nippon

Boehringer Ingelheim Co., Ltd.

When the study participants experienced clinical symptoms of liver injury (such as jaundice, unexplained encephalopathy and coagulopathy, and right upper quadrant pain) without laboratory results (ALT, AST, and total bilirubin), the investigator or subinvestigator will perform extra blood tests as needed and evaluate the aforementioned test parameters. If they meet the criteria for liver injury, procedures in the DILI checklist should be followed. Furthermore, when the study participants with baseline AST and ALT levels within the normal range had an elevation in AST or ALT levels to at least 3 times and <5 times the upper limit of the normal range, they should be reexamined within 72 hours. The DILI checklist is unnecessary except when a liver injury alert of 1, 2, 3, or 4 was detected because of the reexamination.

The study drug must be discontinued immediately when the following events occurred:

- A liver injury alert is 1, 2, or 3.
- A liver injury alert is 4 and lasts for at least 2 weeks.
- AST or ALT levels increase by at least 8 times the upper limit of the normal range.

If the causal relationship between the DILI event and study drug cannot be ruled out after reviewing the DILI checklist, the study drug should be discontinued without rechallenge. When the event was evaluated using the DILI checklist and another cause was identified (e.g., acute viral hepatitis), treatment with the study drug may be resumed as needed.

The study participants with abnormal aminotransaminase levels at baseline (ALT or AST levels ≥1.5 times the upper limit of the normal range) will be evaluated using the DILI checklist for any of the following parameters:

- AST or ALT levels ≥2 times the baseline value or ≥300 U/L (whichever occurs first) and total bilirubin ≥2 times the baseline value;
- AST or ALT levels ≥2 times the baseline value or ≥300 U/L (whichever occurs first) and INR ≥1.2 times the baseline value;
- AST or ALT levels ≥2 times the baseline value or ≥300 U/L (whichever occurs first) and new onset or worsening of signs and symptoms of fatigue, feeling queasy, vomiting, right upper quadrant pain or tenderness, fever, rash, or eosinophilia (≥5%); and
- AST or ALT levels ≥3 times the baseline value.

The study participants with abnormal AST and ALT levels at baseline should immediately discontinue the study drug if any of the following events occurred:

- A liver injury alert of 1, 2, or 3;
- AST or ALT levels ≥5 times the baseline value or ≥500 U/L (whichever occurs first); and

- An increase in AST or ALT levels from baseline and new onset or worsening of signs and symptoms of fatigue, feeling queasy, vomiting, right upper quadrant pain or tenderness, fever, rash, or eosinophilia (≥5%).

If the causal relationship between the DILI event and study drug cannot be ruled out after reviewing the DILI checklist, the study drug should be discontinued permanently without rechallenge.

b. Metabolic acidosis, ketoacidosis, and diabetic acidosis (DKA)

Patients with metabolic acidosis, ketoacidosis, or diabetic ketoacidosis should be subject to detailed investigation based on medical judgment and the clinical course until diagnosis and/or recovery.

Diabetic ketoacidosis is defined using the diagnostic criteria listed in the following table and those specified by the American Diabetes Association (ADA).

The investigator or subinvestigator must pay attention to the following: All the diagnostic criteria indicated in the following table are not always included in those for diabetic ketoacidosis, and clinical judgment should be used. Patients with diabetic ketoacidosis may present with different clinical symptoms due to the mechanism of action of empagliflozin, and diabetic ketoacidosis may occur with lower plasma glucose levels than those shown in the following table.

	Diabetic ketoacidosis (DKA)		
	Mild	Moderate	Severe
Plasma glucose (mg/dL)	>250	>250	>250
Arterial pH	7.25–7.30	7.00–7.24	<7.00
Serum bicarbonate ion (mEq/L)	15–18	10 to <15	<10
Urinary ketones*	Positive	Positive	Positive
Serum ketones*	Positive	Positive	Positive
Effective serum osmolality (mOsm/kg)**	Variable	Variable	Variable
Anion gap**	>10	>12	>12
Changes in hypesthesia or mental obtundation	Awake	Awake or lethargy	Stupor or coma

*Nitroprusside reaction

**Osmotic pressure formula: 2 × Na (mEq/L) + glucose (mg/dL)/18

***Anion gap formula: $(Na^{+}) - (Cl^{-} + HCO_3^{-})$ (mEq/L)

c. Events associated with leg amputation

The definition of an event associated with leg amputation includes amputation (including bone amputation), exarticulation (including joint amputation), and autoamputation (spontaneous separation of necrosis from the lower limb). It excludes wiping of wounds without leg amputation (removal of healed wound or necrotic tissue), stump surgery (such

as stump correction, abscess drainage, and plastic surgery of wound), and other surgeries (e.g., nail resection or removal). Leg amputation, exarticulation, and autoamputation will be reported as an individual event. A serious adverse event report should include the date of surgery, degree of leg amputation or exarticulation, condition leading to the surgery, and risk factors for leg amputation in study participants, if any.

11. Diseases

11.1. Definition of Diseases

Diseases are defined as any disease, disorder, or death or infection with suspected causal relationship with the study and include laboratory abnormalities and various symptoms.

11.2. Procedures for Reporting Serious Diseases to the Certified Review Board

In the event of the following diseases, (1.–3.) that should be reported, the study doctor should promptly report the information to the investigator. Upon receipt of the report described as follows, the investigator will report it to the director of the study site within the specified period and notify the principal investigator. The principal investigator will report it to the Certified Review Board and inform other investigators accordingly. The investigators who received the information should promptly report it to the director of the study site.

In the event of the following diseases, (1.–3.) that should be reported, the study doctor should promptly report the information to the investigator. The investigator will report it to the director of the study site within the specified period and then to the Certified Review Board.

1. The following unexpected events with suspected causal relationship with the study: 7 days
   a. Death
   b. Diseases that may result in death
2. The following events: 15 days
   a. The following events with suspected causal relationship with the study (except those listed in 1):
      (1) Death
      (2) Diseases that may result in death
   b. The following unexpected events with suspected causal relationship with the study:
      (1) Diseases that require inpatient hospitalization or prolongation of existing hospitalization
      (2) Disability
      (3) Diseases that may be incapacitating
      (4) Diseases that are serious next to the events mentioned in (1) to (3); death; and diseases that may result in death
      (5) Congenital disease or abnormality in the next generation
3. Events with suspected causality to the conduct of the study (except those mentioned in 2.): When a periodic report is submitted to the Certified Review Board under the provisions of Article 17, Paragraph 1 of the Act

●Diseases to be reported to the Certified Review Board

Diseases	Unexpected	Expected
a. Death	7 days	15 days
b. Diseases that may result in death	7 days	15 days
c. Diseases that require inpatient hospitalization or prolongation of existing hospitalization	15 days	Periodic report
d. Disability	15 days	Periodic report
e. Diseases that may be incapacitating	15 days	Periodic report
f. Diseases that are serious next to the events mentioned in (c) to (e); death; and diseases that may result in death	15 days	Periodic report
g. Congenital disease or abnormality in the next generation	15 days	Periodic report
h. Other diseases	Periodic report	Periodic report

For changes in reports on diseases such as changes in outcome, the investigator or subinvestigator will inform the director of the study site and principal investigator and the principal investigator will report to the Certified Review Board.

11.3. Procedures for Reporting Serious Diseases to the Minister of Health, Labour and Welfare

Whenever the principal investigator becomes aware of the events listed in 1 and 2a in Section 11.2, he/she should inform the Minister of Health, Labour and Welfare via the screen for reporting diseases on the jRCT within the period specified in the table in the same section.

12. Data Management

12.1. Data Management Plan

A data manager will develop a data set for data cleaning, query creation, and statistical analysis based on the case report forms. For missing values, uncompleted data, and abnormal values indicative of clerical errors, the data manager will set a program for issuing queries to make an inquiry. The data manager will check an answer from the investigator or subinvestigator or review the modified data.

12.2. Central Monitoring

Central monitoring will be performed according to the monitoring procedures.

12.3. Case Report Forms

The investigator or subinvestigator will prepare a case report form for all the study participants who provided informed consent. The EDC system will be used as the case report form.
Before using the EDC system, the investigator, subinvestigator, and research collaborator will receive training.
The investigator or subinvestigator will enter data directly on the EDC system when creating the case report forms.
Changes or revisions of the case report forms will be stored as audit trails documenting the information before and after the change or revision, person who changed or revised the case report form, date of change or revision, and its reason.
The investigator will ensure that the prepared case report forms are accurate and complete and sign (put an electronic signature on) the relevant section of the case report forms. The investigator will take full responsibility for the accuracy and reliability of all data recorded on the case report forms.

12.4. Data Directly Recorded on the Case Report Forms

Data directly recorded on the case report forms will be used as source documents for the following:

- causal relationship between the study and the adverse event, and its seriousness and outcome;
- comments from the investigator or subinvestigator;
- reason for discontinuation in each study participant; and
- other data not recorded on the medical records.

13. Statistical Consideration

13.1. Definition of an Analysis Set

Two different analysis sets, full and safety (FAS and SAS, respectively), will be used in the study.

FAS consists of the study participants who received at least one dose of the study drug and underwent observation, test, and evaluation at least once.

SAS consists of the study participants who received at least one dose of the study drug.

13.2. Data Handling

Data will be handled according to the following procedures. After consultation with the principal investigator and data manager, the person responsible for statistical analysis will determine how to handle data on all study participants in terms of statistical analysis before data lock.

13.2.1. Handling of Measured Values Out of the Predetermined Acceptable Range

For the data that deviated from the acceptable range for endpoint measurement, the person responsible for statistical analysis will consult with the principal investigator and data manager and decide on data adoption and rejection. If multiple data are available within the applicable range, an absolute value of the difference between the scheduled evaluation and relevant days will be calculated and the minimum absolute value will be adopted as data for the time point of evaluation. If the data have the same absolute value, they will be evaluated individually (by endpoint). This section does not apply to follow-up study data.

13.2.2. Handling of Outliers

Handling of outliers will be evaluated and determined before analysis. The application of an appropriate variable transformation or the statistical method not greatly affected by outliers should be considered for some variables.

13.2.3. Handling of Missing Values

No missing data will be imputed.

13.3. Analytical Methods

13.3.1. Study Participants' Demographics

Analytical parameters: Study participants' demographics
Analysis sets: FAS and SAS
Analysis method: Descriptive statistics will be used to summarize demographics and other baseline characteristics.
For continuous variables, the number of study participants, mean, standard deviation, minimum, median, maximum, and interquartile range will be calculated. For categorical variables, the number of study participants, frequency, and percentage will be calculated.

13.3.2. Study Treatment Compliance

Analytical parameter: Study treatment compliance
Analysis sets: FAS and SAS
Analysis method: The duration of study treatment will be summarized. Treatment compliance will be classified into the following: ≥80%, <80%, or other.

13.3.3. Concomitant Drugs and Peritoneal Dialysis

Analytical parameter: Concomitant drugs, peritoneal dialysis
Analysis sets: FAS and SAS
Analysis method: Concomitant drugs and peritoneal dialysis will be summarized.

13.3.4. Primary Endpoint

Analytical parameter: Change from baseline in the amount of water removed per day to the glucose peritoneal dialysate solutions at Week 8
Analysis set: FAS
Analysis method: Mixed-effect model repeated measures (MMRM)
MMRM will be used to estimate the therapeutic effect on changes from baseline in the amount of water removed at Week 8. The model contains treatment, period, and treatment–period interaction as fixed effects and individual as random effects. REML will be used for repeated estimation. An unstructured marginal covariance structure will be specified, and if the model does not converge, the following methods should be tried: (1) use the value obtained using Fisher's scoring method as an initial value in REML; (2) specify the covariance structure in order of Toeplitz, AR (1), and CS. Calculate the standard errors of estimates using sandwich variance estimators, and determine the degrees of freedom using Kenward–Roger's method.
For sensitivity analyses, conduct a similar analysis by excluding the data collected during catheter dysfunction (collection of drainage <80% of the infused amount of peritoneal dialysate

solutions, taking ≥20 minutes to infuse the solutions).

For secondary analysis, MMRM with changes in the mean amount of water removed at Week 4 and Weeks 4 and 8 as objective variables will be used. The model will use the same fixed and random effects as those used in the primary analysis.

A subgroup analysis will be performed using the following factors:

Age (<65 years/≥65 years), sex, presence or absence of diabetes, use of icodextrin-containing peritoneal dialysates, 24-hour urine volume (<200 mL/≥ 200 mL), the glucose load from the peritoneal dialysate (concentration × dose, median), the glucose concentration (median for each of patients using peritoneal dialysis from Baxter International Inc. and TERUMO CORPORATION) in the drainage in FAST PET, and the drainage-to-serum creatinine ratio (median) in FAST PET

13.3.5. Secondary Endpoints

Analytical parameters: Changes in NT-proBNP and BNP
Analysis set: FAS
Analysis method: MMRM
Calculation will be performed in a similar way to that used in the primary endpoint. The model contains the logarithms of NT-proBNP and BNP.

Analytical parameters: Changes in FAST PET-related factors (amount of water removed, sodium, potassium, glucose, urea nitrogen, creatinine, uric acid, protein, IL-6, CA-125, and drainage-to-serum creatinine ratio)
Analysis set: FAS
Analysis method: MMRM
Calculation will be performed in a similar way to that used in the primary endpoint. The model contains the analytical parameters after appropriate variable transformation as needed.

Analytical parameters: Changes in factors related to 24-hour urine collection (urine volume, sodium, potassium, glucose, urea nitrogen, creatinine, uric acid, urine protein, urea and creatinine clearance [the mean of urea and creatinine clearance])
Analysis set: FAS
Analysis method: MMRM
Calculation will be performed in a similar way to that used in the primary endpoint. The model contains the analytical parameters after appropriate variable transformation as needed.

Analytical parameters: Urine KIM-1
Analysis set: FAS
Analysis method: MMRM

After logarithmization, the value 8 weeks after administration is used as the objective variable, and fixed effects, random effects, etc. are calculated in the same way as the primary endpoint.

Analytical parameters: Hemoglobin, hematocrit, ferritin, and transferrin saturation
Analysis set: FAS
Analysis method: MMRM
Calculation will be performed in a similar way to that used in the primary endpoint. The model contains the logarithms of ferritin and transferrin saturation.

Analytical parameters: Body weight and systolic and diastolic blood pressure
Analysis set: FAS
Analysis method: MMRM
Calculation will be performed in a similar way to that used in the primary endpoint.

Analytical parameters: Intracellular and extracellular fluid volume, and fluid overload
Analysis set: FAS
Analysis method: MMRM
Calculation will be performed in a similar way to that used in the primary endpoint.

13.3.6. Adverse Events

Analytical parameters: Adverse events
Analysis set: SAS
Analysis method: A list of adverse events by study participant (event name, date of onset, date of outcome, duration, outcome, severity, seriousness, and causal relationship with the medicine) will be provided by assigned treatment.
Adverse events by event and the number of subjects with adverse events, number of episodes of adverse events, and incidence by severity will be tabulated by assigned treatment. For tabulation by severity, the highest grade of each adverse event will be used. The duration of adverse events is defined as “date of outcome − date of onset + 1” (unit, day).
The adverse events whose causal relationship with the drug cannot be ruled out and serious adverse events will be tabulated similarly.

13.4. Interim Analysis and Criteria for Premature Discontinuation

No interim analysis will be performed in the study.

13.5. Changes to the Statistical Analysis Plan

For changes or addition of analyses after the start of the study, the principal investigator will

examine their validity and effects on the study, consult with the person responsible for statistical analysis to amend the protocol, and explain the circumstances of the changed analysis plan in its clinical study report of the study.

14. Quality Control and Quality Assurance

14.1. Quality Control Policies

The study will be conducted in compliance with the Clinical Trials Act and Enforcement Regulations of the Clinical Trials Act. Therefore, quality control policies will be established to meet the requirements set out in these laws and regulations.

14.2. Quality Objectives

The documents required by the Clinical Trials Act should be prepared and stored properly, and the requirements for protection of study participants should be met. Allowing for a dropout (including deviation) of 20%, the study will enroll enough study participants to analyze the primary endpoint.

14.3. Monitoring

To examine the progress of the study and determine whether it is conducted in accordance with the laws and regulations and protocol to ensure the reliability of the study and the study is conducted properly from the viewpoint of study participant protection, the principal investigator will appoint a person responsible for monitoring and instruct the monitor to conduct monitoring activities after preparing the monitoring procedures.
The person responsible for monitoring will conduct monitoring activities according to the monitoring procedures and keep a record of the monitoring results (a monitoring report).
The investigator or director of the study site will ensure that the person responsible for monitoring is granted access to source documents.

14.4. Audit

To determine whether the study is conducted in accordance with the laws and regulations and protocol to ensure the reliability of the study and the data collected during the study from the viewpoint of study participant protection, the principal investigator will appoint an auditor and instruct the auditor to conduct an audit after preparing the audit procedures.
The principal investigator should not instruct the person involved in the conduct of the study or monitoring activities to conduct an audit.
The auditor will conduct an audit according to the audit procedures.
The investigator or director of the study site will ensure that the auditor is granted access to

source documents.

14.5. Response to Inspection by the Regulatory Authorities, etc.

Upon request of the Certified Review Board or its designee and the Minister of Health, Labour and Welfare or its designee, the investigator or director of the study site will accept the regulatory authority inspection and ensure that they are granted access to source documents and other necessary materials.

14.6. Nonconformity

14.6.1. Definition of Nonconformity

Nonconformity is defined as noncompliance with the Enforcement Regulations of the Clinical Trials Act, protocol, procedures, and other documents and study data falsification, fabrication, etc. Nonconformity should be managed according to the following steps.

14.6.2. Critical Nonconformity

Critical nonconformity is defined as nonconformity affecting the human rights and safety of study participants and progress and reliability of the study (i.e., noncompliance with the inclusion and exclusion or discontinuation criteria and prohibited concomitant therapies). It excludes protocol deviations occurring for unavoidable medical reasons, such as steps performed to help study participants avoid emergency.
The principal investigator must seek comments from the Certified Review Board when becoming aware of these incidents or events that were considered critical nonconformity.

14.6.3. Nonconformity Management Procedures

The investigator will record all nonconformities discovered during the study.
The subinvestigator will promptly report the discovered nonconformity to the investigator.
Upon receipt of the nonconformity report, the investigator will promptly report to the director of the study site and notify the principal investigator.
The principal investigator will promptly provide information about the reported nonconformity to other investigators and make a periodic report on the occurrence of nonconformity and subsequent action to the Certified Review Board.

15. Ethical Consideration

15.1. Various Rules to be Followed

The study will be conducted in compliance with the ethical principles of the Declaration of Helsinki and in accordance with the Clinical Trials Act, Enforcement Regulations of the Clinical Trials Act, and other relevant notifications. The investigator and subinvestigator will administer protocol treatment in compliance with the protocol.

15.2. Approval of the Certified Review Board and Director of the Study Site

The appropriateness of conducting this study will be reviewed and approved by the Certified Review Board, then it will be reviewed by the director of the study site. After published on jRCT, the study will be conducted.

15.3. Study Cost Burden on the Study Participants

(1) Boehringer Ingelheim Pharma GmbH & Co. KG will provide the study drugs for free, and no financial burden will be imposed on the study participants.
(2) Because the measurement of IL-6 and CA-125 levels in drained peritoneal dialysate solutions, serum NT-proBNP, and urine KIM-1 will be covered by research funds for the study, no financial burden will be imposed on the study participants.
(3) Study participants will receive a gift card worth 10,000 yen at the start of the drug administration as a reward for their participation in the study.

15.4. Informed Consent Forms and Consent of Study Participants

15.4.1. Procedures for Obtaining Informed Consent

Before obtaining informed consent from the study participant, the investigator will prepare an informed consent form and ask the Certified Review Board to approve it.
On the basis of the informed consent form approved by the Certified Review Board for the study, the investigator or subinvestigator will provide an explanation to the study participant and allow him/her plenty of time to consider the study. After ensuring that the study participant fully understands the nature of the study, the investigator or subinvestigator will ask him/her to participate in the study.
When the study participant agrees to participate in the study, the investigator or subinvestigator will ask him/her to sign a prescribed consent form.
The investigator or subinvestigator will ensure that the name of the physician who explained the consent procedures, date of explanation, and date of informed consent are recorded on the consent form.
The study participants, based on their own free will, may withdraw their consent at any time

even after they have agreed to participate in the study. For consent withdrawal, the study participants will sign a prescribed consent withdrawal form as much as possible and submit it to the investigator or subinvestigator.
Copies of the consent and consent withdrawal forms will be handed to the study participant. The original consent and withdrawal consent forms should be properly stored as specified by the study site.
When the informed consent form was revised on the basis of the information that may influence the study participant's willingness to participate in the study after his/her consent has been obtained, the investigator or subinvestigator should provide an explanation to the study participant to obtain another informed consent from him/her using the informed consent form (revised version) reviewed and approved by the Certified Review Board.

15.4.2. Information that Should be Included in the Informed Consent Form

The informed consent form should include the following information:

(1) the name of the study and a statement that the director of the study site has approved the conduct of the study and that the study plan has been submitted to the Minister of Health, Labour and Welfare,
(2) the name of the study site and name and job title of the investigator (including the names of the collaborating study center and investigator),
(3) the purpose and significance of the study,
(4) overview of the drugs, etc.,
(5) the method (including the purpose of collecting samples and information from the study participants) and duration of the study;
(6) the reason why the patient is chosen as a study participant;
(7) the burden imposed on the study participant and reasonably expected risks and benefits;
(8) that the study participant may withdraw from the study at any time after providing his/her informed consent to participate in or continue their participation in the study;
(9) that the study participant may refuse to provide informed consent or withdraw his/her informed consent to participation or continued participation in the study without any penalty;
(10) method of the study information disclosure;
(11) that the study participant is allowed to obtain or have access to the protocol and documents about study procedures, unless he/she may violate the confidentiality of personal information on other study participants and destroy the originality of the study, at the request of the participant, and method of information collection and access;
(12) handling of personal information, etc. (including the method of de-identification as needed);

(13) method of sample and information storage and disposal;
(14) notice of conflict of interest in the conduct of the study by the study site, including a source of money for the study as well as a notice of conflict of interest in the conduct of the study by the investigator and other personnel, including personal profit;
(15) response to complaints and inquiries, including consultations by the study participants and concerned parties;
(16) any financial burden on the study participant or rewards;
(17) alternative treatments and their description as well as their reasonably expected benefits and inconveniences;
(18) compensation for injury caused by the study and its details;
(19) topics to be reviewed by the Certified Review Board responsible for reviewing and making comments on the specified clinical research and other information on the Certified Review Board related to the specified clinical research;
(20) the terms of the contract stipulated in Article 32 of the Clinical Trials Act; and
(21) other information necessary for conducting the specified clinical research.

15.5. Contact Information for Study Participants

The investigator or subinvestigator will answer questions from the study participants and concerned parties.

15.6. Anticipated Advantages and Disadvantages for Study Participants

15.6.1. Anticipated Advantages

The amount of water removed may increase in both groups during empagliflozin treatment. Because the study participants receive the standard of care for chronic heart failure during the placebo treatment and washout period, they may gain benefits equivalent to those offered during routine medical practice.
If the study demonstrates the efficacy and safety of empagliflozin in patients with chronic heart failure on PD, the drug may serve as a new treatment option for such patients.

15.6.2. Anticipated Disadvantages

The amount of blood collected may be greater than those during routine medical practice (approximately a few cubic centimeter) and frequent implementation of FAST PET or 24-hour urine collection may impose more physical and mental burdens on the study participants and prolong their hospital stay.
Treatment with empagliflozin may cause the adverse events described in Section 10.6.
The study participants will record the amount of water removed in the notebook, which is also

performed under routine medical practice, and they would not have an additional burden placed on them due to the study.

15.6.3. Overall Assessment of Advantages and Disadvantages and Measures to Minimize the Disadvantages

Empagliflozin is generally considered well tolerated, and the large-scale study has not reported any increase in the occurrence of adverse drug reactions associated with renal impairment. [19] In fact, an overseas report on SGLT2 inhibitors administered to PD patients [24] and our other study have not shown any serious adverse drug reactions, and its disadvantages are less likely to outweigh its advantages. To minimize the disadvantages, the study participants will be monitored on an outpatient basis every month. Before participating in the study, the study participants will be fully informed of the disadvantages, and their willingness to participate in the study will be confirmed.

15.7. Confidentiality of Study Participants (Protection of Personal Information)

When raw data and consent forms are handled during the conduct of the study, due confidentiality consideration should be provided to the study participants. Study participant identification codes will be assigned to the patients during enrollment. The study participant identification codes are composed of figures and symbols irrelevant to personally identifiable information, such as an initial and chart ID, and they will be used for creating study-related documents, including case report forms, to prevent individuals from being identified. The investigator will keep documents, such as a correspondence table, under lock and key to prevent personal information, including the name of the study participant, from being disclosed to the public. When the study results are published, they should not contain the information that can be used to identify the study participants.

16. Compensation for Study-related Injury

If the study participant has any study-related injury, the investigator, subinvestigator, and study site will provide medical care and take other necessary measures so that the study participant can immediately receive an appropriate diagnosis and treatment and necessary measures may be provided. The treatment to be provided will be covered by health insurance.

To compensate for study-related injuries to the study participants, the principal investigator will take out the clinical study insurance covering the liability to pay compensation described as follows and provide compensation according to the terms of payment for the clinical study insurance (compensation insurance).

(1) Compensation money for the study participant's death or sequelae

(2) Medical expenses and medical allowances necessary to treat study-related injuries to the study participants. However, such treatments will be covered by health insurance, and the study participants will receive medical allowances as compensation for the amount of copayments for their treatments and cost burden other than the cost of care.

The investigator or subinvestigator will provide a statement that the study participants may receive compensation in the event of study-related injuries in the informed consent form, prepare a document outlining the compensation, and provide it to the study participants when providing an explanation to obtain their informed consent.

17. Discontinuation or Termination of the Entire Study

17.1. Discontinuation Criteria

The principal investigator will discontinue or interrupt the study as needed in any of the following criterion:

(1) When new important information becomes available that may affect adversely the safety of the study participants or the conduct of the study (For example, expected adverse events [diseases] or nonconformity occurs much more frequently than expected.)

(2) When it is considered extremely difficult to achieve the target number of study participants (For example, study participants are enrolled at a markedly slower rate than expected.)

(3) When the Certified Review Board commented that the study should be discontinued

(4) If a case arises where the study needs to be discontinued or interrupted

17.2. Discontinuation Procedure

When the discontinuation criteria were found to be met and discontinuation of the entire study was determined, the principal investigator will promptly notify the investigator accordingly. After informing the study participants of study discontinuation, the investigator or subinvestigator will conduct the protocol-specified observation, test, and evaluation as much as possible and perform medical procedures, such as alternate treatment, as needed.
The principal investigator will submit a notice of discontinuation to the Certified Review Board within 10 days of the date when discontinuation of the entire study was determined and provide a notice to the Minister of Health, Labour and Welfare.

17.3. Termination Criteria

The date of study termination is defined as the day of publication when the principal investigator recorded the summary of the clinical study report on jRCT.

18. Study Information Disclosure and Publication of Results

18.1. Study Registration

The study will be registered with jRCT beforehand. The study information will be updated as appropriate in line with changes in the protocol and the progress of the study.

18.2. Publication of Study Results

18.2.1. Primary Endpoint Reports

The principal investigator will prepare a primary endpoint report within 1 year of the end date of data collection for the primary endpoint as a general rule, seek opinions from the Certified Review Board, and submit it to the director of the study site. The primary endpoint report will be recorded on jRCT for publication within 1 month of the day when the Certified Review Board offered an opinion.
The principal investigator will promptly report its publication to the director of the study site and inform other investigators accordingly. The investigators will promptly report the information to the director of the study site.
Because the primary endpoint report is scheduled to be prepared simultaneously with the clinical study report in the study, the creation of the primary endpoint report will be considered complete if the clinical study report has been prepared.

18.2.2. Clinical Study Report

The principal investigator will prepare a clinical study report and its summary within 1 year of the end date of data collection for all endpoints as a general rule.
The clinical study report should contain at least all the following items:
(1) background information about the study participants (such as age and sex);
(2) information about the progress of the study depending on its design (such as changes in the number of study participants);
(3) summary of the occurrence of diseases; and
(4) data analysis and results of the primary and secondary endpoints.

The principal investigator will seek an opinion from the Certified Review Board about the clinical study report and its summary and submit them to the director of the study site. The summary of the clinical study report, protocol, and statistical analysis report (if prepared) will be recorded on jRCT for publication within 1 month of the day when the Certified Review Board offered an opinion.
The principal investigator will promptly report its publication to the director of the study site and inform other investigators accordingly. The investigators will promptly report the

information to the director of the study site.

18.2.3. Presentation at Scientific Meetings, etc.

The outcome of the study will be promptly presented at scientific meetings and published in journals. The necessary measures should be taken to protect the human rights and interests of the study participants and concerned parties before presentation and publication. Researchers who present the study results at scientific meetings and publish them as authors will be determined according to the degree of their contribution to the study.

19. Change Control

19.1. Changes in Documents Approved by the Certified Review Board

A change application should be submitted to the Certified Review Board when the documents approved by the Certified Review Board are revised. When the Certified Review Board offered an opinion, it should be promptly reported to the director of the study site.

19.2. Changes in the Study Plan

For changes (except minor ones specified by the Ordinance of Ministry of Health, Labour and Welfare) in the study plan, the principal investigator will seek an opinion from the Certified Review Board described in this protocol and submit the changed study plan and notification based on Form 2 in advance.

19.3. Minor Changes in the Study Plan

For minor changes in the study plan, the principal investigator will notify the Certified Review Board described in this protocol of the changes and provide a notice to the Minister of Health, Labour and Welfare within 10 days of the changes in accordance with Article 42 of the Enforcement Regulations of the Clinical Trials Act.

20. Conflict of Interest

20.1. Source of Money for the Study

Nippon Boehringer Ingelheim Co., Ltd. will provide funds, and Boehringer Ingelheim Pharma GmbH & Co. KG will provide drugs, and services (such as drug assignment and packaging) for the study. The study will be conducted from a medical viewpoint and does not benefit or accommodate the company.

20.2. Conflict of Interest Management

After the facts regarding the study are confirmed by each study site, a conflict of interest management plan will be prepared and an appropriate control will be implemented by seeking an opinion from the Certified Review Board.
The conflict of interest status for the study is as shown in Attachment 1.

21. Periodic Reports Submitted to the Certified Review Board and Minister of Health, Labour and Welfare

For implementing the specified clinical research, the investigator will report the following items to the director of the study site and then the Certified Review Board and Minister of Health, Labour and Welfare.

21.1. Periodic Reports Submitted to the Certified Review Board

21.1.1. Items to be Included in Periodic Reports

(1) the number of study participants who took part in the study
(2) the occurrence of diseases related to the study and subsequent clinical courses;
(3) the occurrence of nonconformity to this Ministerial Ordinance related to the study or protocol and subsequent action;
(4) evaluation of the safety and scientific appropriateness of the study; and
(5) information about the involvement of the manufacturers of the drugs, etc., and others in the study.

21.1.2. Timing of Periodic Reports

In principle, a periodic report will be submitted to the Certified Review Board every year, counted from the day when the study plan was submitted to the Minister of Health, Labour and Welfare, within 2 months of the termination of the period.

21.2. Periodic Reports Submitted to the Minister of Health, Labour and Welfare

21.2.1. Items to be Included in Periodic Reports

(1) the name of the Certified Review Board described in the protocol
(2) the appropriateness of continuing the study determined by the Certified Review Board, and
(3) the number of study participants who participated in the study.

21.2.2. Timing of Periodic Reports

A periodic report will be submitted to the Minister of Health, Labour and Welfare within 1 month of the day when the Certified Review Board offered an opinion.

22. Method of Storing and Disposing of Documents, Records, etc.

22.1. Storage of Source Documents

Source documents refer to original records and data on clinical findings obtained from the application of drugs, etc., to the study participants and their treatment, observations, and other activities.
The investigator or director of the study site will store the following documents, including source documents and those specific to the study, for an inspection or audit by the Certified Review Board and regulatory authorities or their designee.
These documents include a list of study participant identification codes, medical records, original consent forms signed and dated, and electronic copies of electronic case report forms, including audit trails.
The investigator or director of the study site will store essential documents that must be retained until 5 years have elapsed since the discontinuation or termination of the study.
For correction of the aforementioned documents, records, etc., the investigator should record the name of the person who corrected them and date of correction and store them with the corrected records.

22.2. Storage of Records and Documents Required by Law

The investigator will properly store the records, documents, etc., on the study deliberations to prevent them from being leaked, mixed, stolen, or lost until 5 years have elapsed since the discontinuation or termination of the study. The following documents will be stored for the study:

(1) Documents on the identification of the study participants
(2) Documents on the treatment of the study participants and test
(3) Documents on the study participation
(4) Documents on the administration of the drugs, etc., to be studied to the study participants
(5) Documents received by the Certified Review Board over the review of and feedback on the study
(6) The protocol, study plan, and documents on the explanations provided to the study participants and their informed consent
(7) The clinical study report and other documents (or copies) prepared by the investigator as stipulated in the Enforcement Regulations of the Clinical Trials Act
(8) Documents on monitoring and audit
(9) Source documents, etc., other than the documents listed in (1) to (4) above
(10) The contract for conducting the study

(11) Documents on the summary of the drugs, etc., used in the study
(12) Documents on the acquisition of the drugs, etc., to be studied (quantity and date)
(13) Documents on the disposal of the drugs, etc., to be studied (if destroyed)
(14) Other documents necessary for conducting the study

For correction of these records, the investigator should record the name of the person who corrected them and date of correction and store them with the corrected records.

22.3. Storage of Samples

Samples refer to biological samples, such as blood, collected according to the protocol. The investigator or director of the study site will properly store the samples according to the protocol or procedures for storing samples, etc. (The samples will be kept under lock and key at the Department of Nephrology of Osaka University Hospital.)

22.4. Secondary Use of Samples and Information

Samples and information obtained from the study may be used for a different study purpose (secondary use). This should be provided on the informed consent form, and informed consent should be obtained from the study participants after an explanation. A new protocol must be prepared, and approval should be obtained from the Independent Ethics Committee to be consulted on the study before secondary use.

22.5. Disposal Procedures and Method

The investigator should take the necessary measures to de-identify personal data when disposing of the samples and information collected from the study participants during the study and records and documents on the study deliberations.

The director of the study site will store samples, information, records, documents, etc., to be retained until the investigator informs the director that their retention is no longer necessary.

23. Ownership of the Study Results

Intellectual property rights arising from the study, such as a patent right, remain the property of Osaka University.

The ownership of the study results will be specified in the contract between Nippon Boehringer Ingelheim Co., Ltd., the financial contributor, and the institution to which the principal investigator belongs. After the end of the study, a clinical study completion report will be submitted to Nippon Boehringer Ingelheim Co., Ltd., the financial contributor. When publishing the information obtained from the study, the principal investigator shall gain consent from Nippon Boehringer Ingelheim Co., Ltd. under the contract.

24. Study Administrative Structure

24.1. Principal Investigator

Name	Yohei Doi
Affiliation	Department of Nephrology, Osaka University Hospital
Job title	Specially-Appointed Assistant Professor
Address	2-15 Yamadaoka, Suita, Osaka
Telephone No.	+81-6-6879-5111

24.2. Investigators

See Attachment 2 (list of study sites and investigators).

24.3. Person Responsible for Randomization

Name	Chitose Yoshino
Affiliation	intellim Corporation, Clinical Development Division, Oncology Development 1, Group 1
Job title	Group Manager
Address	ORIX Ueno 1-chome Building, 1-1-10 Ueno, Taito-ku, Tokyo
Telephone No.	+81-3-5688-7230

24.4. Person Responsible for Statistical Analysis

Name	Takahide Kudo
Affiliation	intellim Corporation, Data Science Division, Bio-Statistics Group
Job title	Manager
Address	Mainichi intecio , 3-4-5 Umeda, Kita-ku, Osaka, Osaka
Telephone No.	+81-3-5688-7230

24.5. Person Who Summarizes the Clinical Study Except the Principal Investigator and Investigators

Name	Yoshitaka Isaka
Affiliation	Department of Nephrology, Osaka University Hospital
Job title	Professor
Address	2-15 Yamadaoka, Suita, Osaka
Telephone No.	+81-6-6879-5111

24.6. Person Responsible for Data Management

Name	Satoshi Kanda
Affiliation	intellim Corporation, Data Science Division, Data Management Group
Job title	Group Manager
Address	ORIX Ueno 1-chome Building, 1-1-10 Ueno, Taito-ku, Tokyo
Telephone No.	+81-3-5688-7230

24.7. Person Responsible for Monitoring

Name	Hideyuki Kiriki
Affiliation	intellim Corporation, Clinical Development Division
Job title	Manager
Address	ORIX Ueno 1-chome Building, 1-1-10 Ueno, Taito-ku, Tokyo
Telephone No.	+81-3-5688-7230

24.8. Person Responsible for Audit

Name	Keisuke Tuda
Affiliation	Audit Office, Department of Medical Innovation, Osaka University Hospital
Job title	Specially-Appointed Researcher Fellow (full-time)
Address	2-2 Yamadaoka, Suita, Osaka
Telephone No.	+81-6-6210-8289

24.9. Coordinating Administrator

Name	Kento Asano
Affiliation	Center of Medical Translational and Clinical Research, Department of Medical Innovation, Osaka University Hospital
Job title	Vice Director of the Center
Address	2-2 Yamadaoka, Suita, Osaka
Telephone No.	+81-6-6210-8290

24.10. Laboratory Related to the Clinical Study

Laboratory	H.U. Frontier, Inc. (SRL)
Address	Akasaka inter-city AIR, 1-8-1 Nishi-Akasaka, Minato-ku, Tokyo
Laboratory	LSI Medience Corporation

Address	1-2-3 Shibaura, Minato-ku, Tokyo

24.11. Contract Research Organization

Contract research organization	intellim Corporation
Address	ORIX Ueno 1-chome Building, 1-1-10 Ueno, Taito-ku, Tokyo
Outsourced duties	Monitoring, data management, and statistical analysis
Method of audit	Follow procedures for each duty, and implement the duties.

25. References

[1] Tsutsui H, et al., "JCS/JHFS 2021 Guideline Focused Update on Diagnosis and Treatment of Acute and Chronic Heart Failure," Circ J. 2021;85:2252-2291.

[2] Heidenreich PA, et al., "2022 AHA/ACC/HFSA Guideline for the Management of Heart Failure: Executive Summary: A Report of the American College of Cardiology/American Heart Association Joint Committee on Clinical Practice Guidelines," J Am Coll Cardiol. 2022;79:1757-1780.

[3] Heidenreich PA, et al., "2022 AHA/ACC/HFSA Guideline for the Management of Heart Failure: A Report of the American College of Cardiology/American Heart Association Joint Committee on Clinical Practice Guidelines," J Am Coll Cardiol. 2022;79:e263-e421.

[4] GBD 2017 Disease and Injury Incidence and Prevalence Collaborators, "Global, regional, and national incidence, prevalence, and years lived with disability for 354 diseases and injuries for 195 countries and territories, 1990-2017: a systematic analysis for the Global Burden of Disease Study 2017," Lancet. 2018;392:1789-1858.

[5] Okura Y, et al., "Impending epidemic: future projection of heart failure in Japan to the year 2055," Circ J. 2008;72:489-491.

[6] Shiba N, et al., "Trend of westernization of etiology and clinical characteristics of heart failure patients in Japan--first report from the CHART-2 study," Circ J. 2011;75:823-833.

[7] Ministry of Health, Labour and Welfare, "Monthly report summary of Vital Statistics (annual total; rough figures) (2021)," [Online]. Available: https://www.mhlw.go.jp/toukei/saikin/hw/jinkou/geppo/nengai21/index.html.

[8] Hanafusa N, et al., "Annual Dialysis Data Report, JSDT Renal Data Registry (as of December 31, 2020)," J. Jpn. Soc. Dial. Ther. 2021;54:611-657.

[9] Enia G, et al., "Long-term CAPD patients are volume expanded and display more severe left ventricular hypertrophy than haemodialysis patients," Nephrol Dial Transplant. 2001;16:1459-1464.

[10] Biesen WV, et al., "Evolution over time of volume status and PD-related practice patterns in an incident peritoneal dialysis cohort," Clin J Am Soc Nephrol. 2019;14:882–893.

[11] Kawaguchi Y, et al., "Searching for the reasons for drop-out from peritoneal dialysis: a nationwide survey in Japan," Perit Dial Int. 2003;23 Suppl 2:S175-177.

[12] Packer M, et al., "Cardiovascular and renal outcomes with empagliflozin in heart failure.," N Engl J Med 2020;383:1413-24.

[13] Anker SD, et al., "Empagliflozin in heart failure with a preserved ejection fraction," N Engl J Med. 2021;385:1451-1461.

[14] Banerjee D, et al., "Personalizing heart failure management in chronic kidney disease patients,"

Nephrol Dial Transplant. 2022;37:2055–2062.

[15] Ito Y, et al., "Peritoneal Dialysis Guidelines 2019 Part 1 (Position paper of the Japanese Society for Dialysis Therapy)," Ren Replace Ther. 2021;7:40.

[16] Chuasuwan A, et al., "Comparisons of quality of life between patients underwent peritoneal dialysis and hemodialysis: a systematic review and meta-analysis," Health Qual Life Outcomes. 2020;18:191.

[17] Zannad F, et al., "Cardiac and kidney benefits of empagliflozin in heart failure across the spectrum of kidney function: Insights from EMPEROR-reduced," Circulation. 2021;143:310–321.

[18] Zelniker TA, et al., "Mechanisms of cardiorenal effects of sodium-glucose cotransporter 2 inhibitors: JACC state-of-the-art review," J Am Coll Cardiol. 2020;75:422–434.

[19] Zhou Y, et al., "SGLT-2 inhibitors reduce glucose absorption from peritoneal dialysis solution by suppressing the activity of SGLT-2," Biomed Pharmacother. 2019;109:1327–1338.

[20] Balzer MS, et al., "SGLT2 inhibition by intraperitoneal dapagliflozin mitigates peritoneal fibrosis and ultrafiltration failure in a mouse model of chronic peritoneal exposure to high-glucose dialysate," Biomolecules. 2020;10:1573.

[21] Shentu Y, et al., "Empagliflozin, a sodium glucose cotransporter-2 inhibitor, ameliorates peritoneal fibrosis via suppressing TGF-β/Smad signaling," Int Immunopharmacol. 2021;93:107374.

[22] "ClinicalTrials.gov Identifier: NCT05374291," [Online]. Available: https://clinicaltrials.gov/ct2/show/NCT05374291.

[23] Borkum M, et al., "The rationale for the need to study sodium-glucose co-transport 2 inhibitor usage in peritoneal dialysis patients," Perit Dial Int. 2022. doi: 10.1177/08968608221096556.

[24] Alhwiesh AK, et al., "The use of SGLT2 inhibitors in peritoneal dialysis patients: a shade of light on dapagliflozin," Arch Nephrol Urol. 2022;5:1-8.

[25] Ateş K, et al., "Effect of fluid and sodium removal on mortality in peritoneal dialysis patients," Kidney Int. 2001;60:767–776.

[26] Lin X, et al., "Daily peritoneal ultrafiltration predicts patient and technique survival in anuric peritoneal dialysis patients," Nephrol Dial Transplant. 2010;25:2322–2327.

[27] Macha S, et al., "Pharmacokinetics, pharmacodynamics and safety of empagliflozin, a sodium glucose cotransporter 2 (SGLT2) inhibitor, in subjects with renal impairment," Diabetes Obes Metab. 2014;16:215-222.

[28] Nippon Boehringer Ingelheim Co ., Ltd., "Documents on Jardiance Tablets 10 mg," [Online]. Available: https://www.pmda.go.jp/drugs/2021/P20211122002/index.html.

Attachment 1 (conflict of interest)

Study title: The effects of empagliflozin on ultrafiltration in patients with peritoneal dialysis: a randomized, double-blind, crossover trial (EMPOWERED trial)

Principal investigator: Yohei Doi, Department of Nephrology, Osaka University Hospital

COI with the manufacturers of the drugs, etc., to be studied that the investigator and subinvestigator in the study site should disclose: Not applicable

Attachment 2 (list of study sites and investigators)

	Study site		Investigator	
	Name of the medical institution and affiliation	Address and telephone no.	Name	Job title
1	Department of Nephrology, Osaka University Hospital	2-15 Yamadaoka, Suita, Osaka<br>+81-6-6879-5111	Yohei Doi	Specially Appointed Assistant Professor
2	Division of Hypertension and Nephrology, National Cerebral and Cardiovascular Center	6-1 Kishibe-Shinmachi, Suita, Osaka<br>+81-6-6170-1070	Fumiki Yoshihara	Director
3	Department of Kidney Disease and Hypertension, Osaka General Medical Center	3-1-56 Bandaihigashi, Sumiyoshi-ku, Osaka-shi, Osaka<br>+81-6-6692-1201	Yoshiyasu Udeda	Director
4	Department of Nephrology, Matsuyama Red Cross Hospital	1 Bunkyo-cho, Matsuyama-shi, Ehime<br>+81-89-924-1111	Taro Kamimura	Director

First version (prepared on May 23, 2023)
